# Supplementary figures and images for: RNA helicase SKIV2L limits antiviral defense and autoinflammation elicited by the OAS-RNase L pathway (part 1 of 2)
Source: EMBO J. 2024 Aug 7;43(18):3876–94. doi: 10.1038/s44318-024-00187-1 (PMC11405415; doi:10.1038/s44318-024-00187-1)

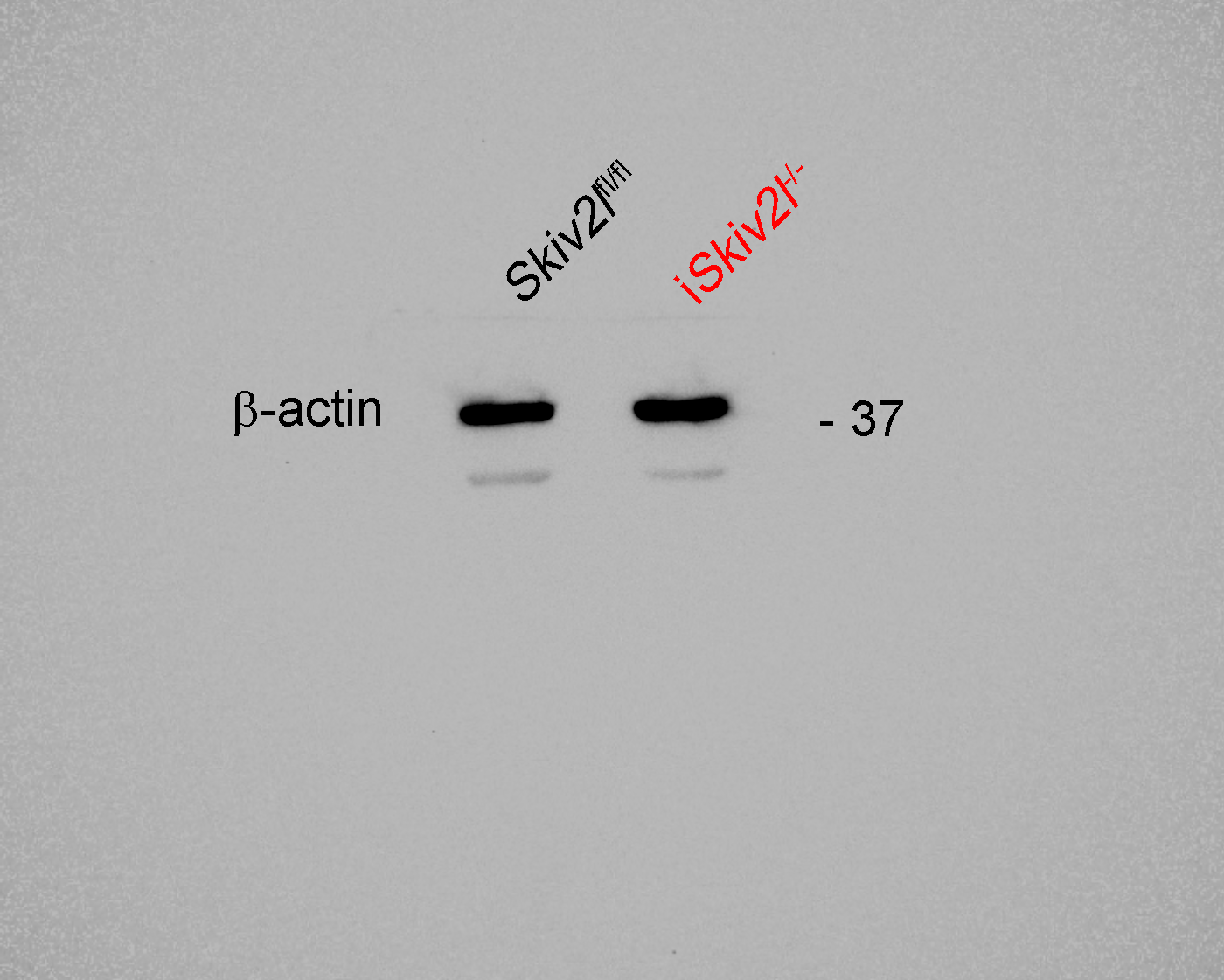

Supplement: Supplementary file 2 — Source data Fig. 1 [file 44318_2024_187_MOESM2_ESM.zip › Figure1/1H/Actin.tif]

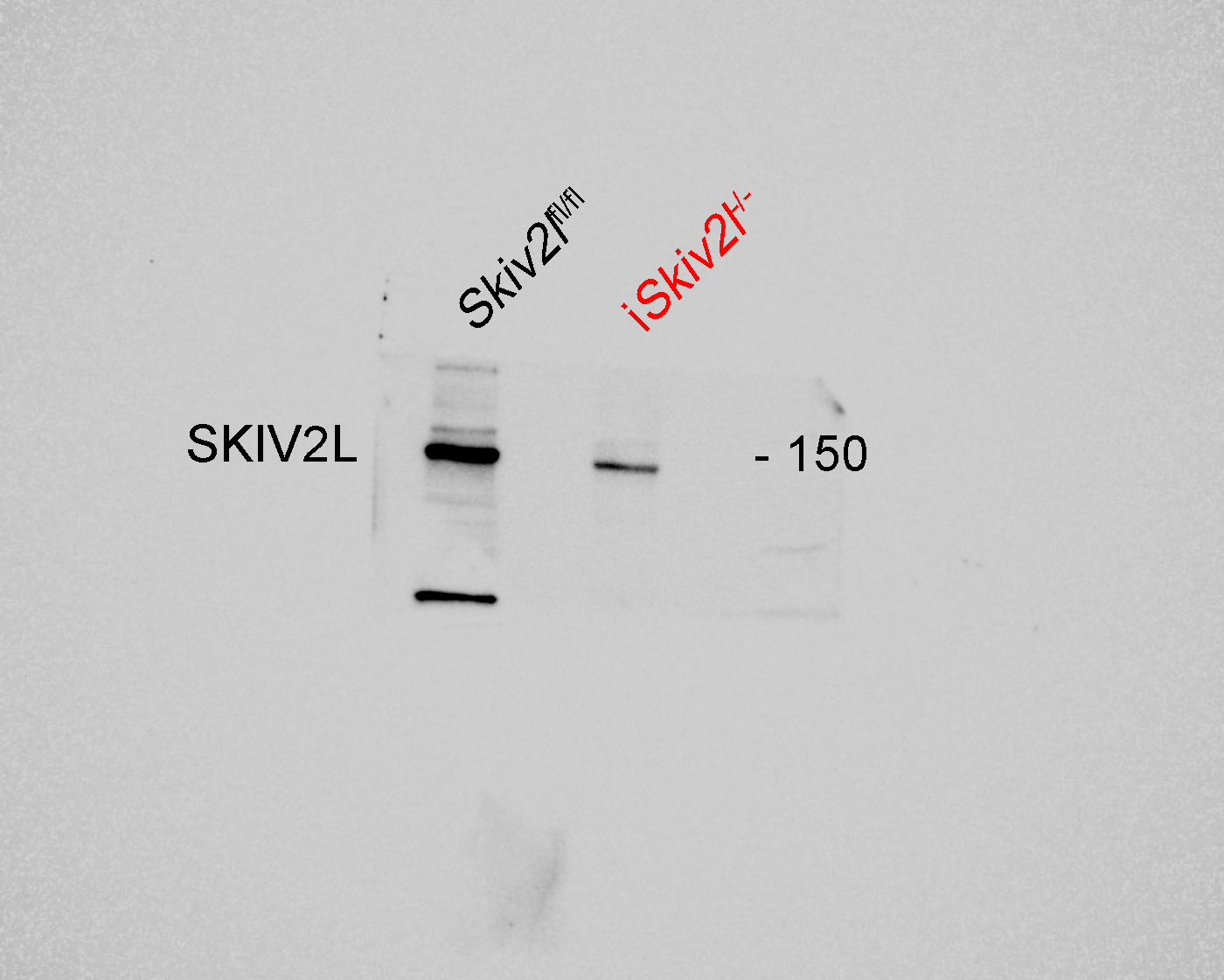

Supplement: Supplementary file 2 — Source data Fig. 1 [file 44318_2024_187_MOESM2_ESM.zip › Figure1/1H/SKIV2L.tif]

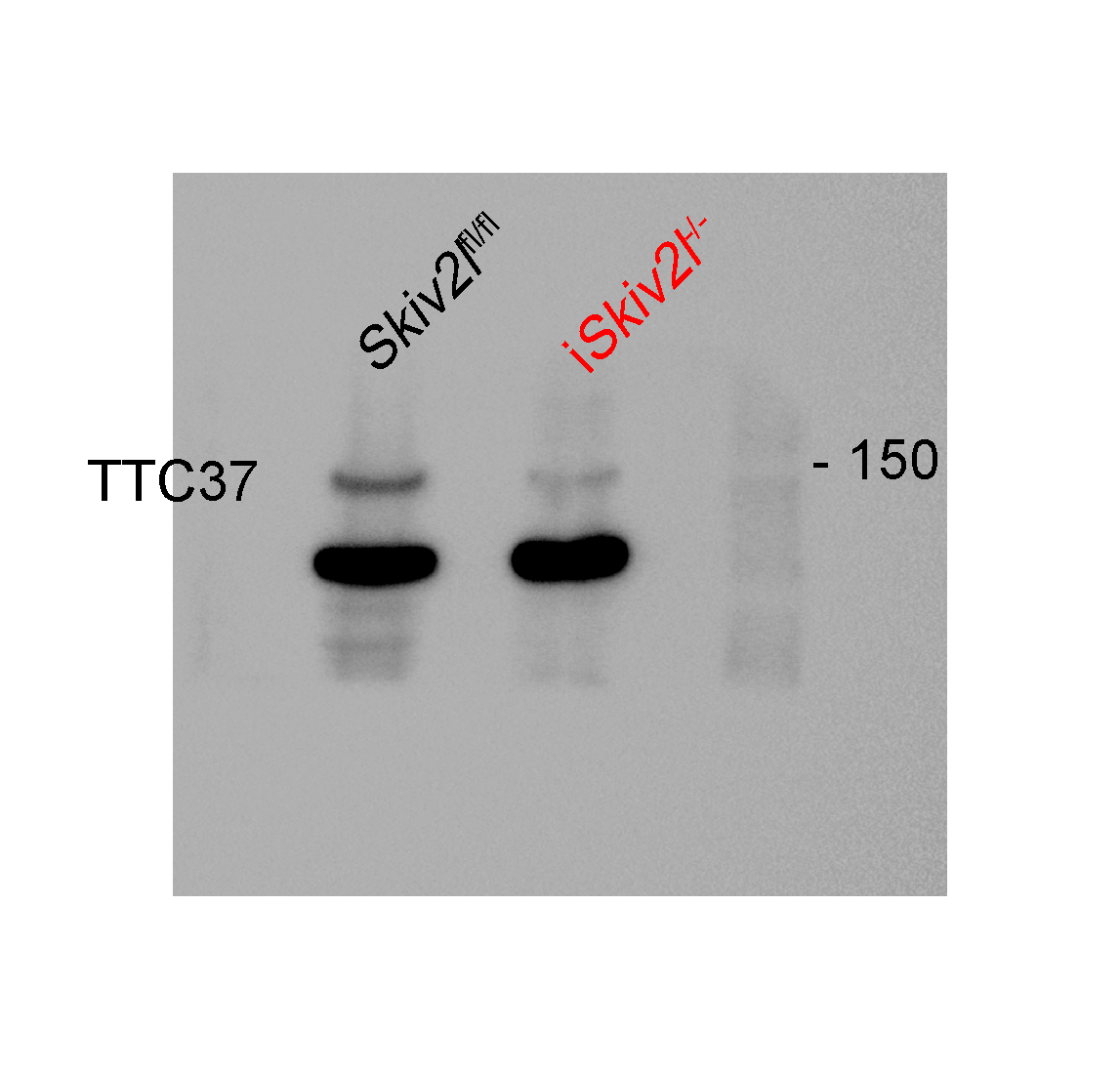

Supplement: Supplementary file 2 — Source data Fig. 1 [file 44318_2024_187_MOESM2_ESM.zip › Figure1/1H/TTC37.tif]

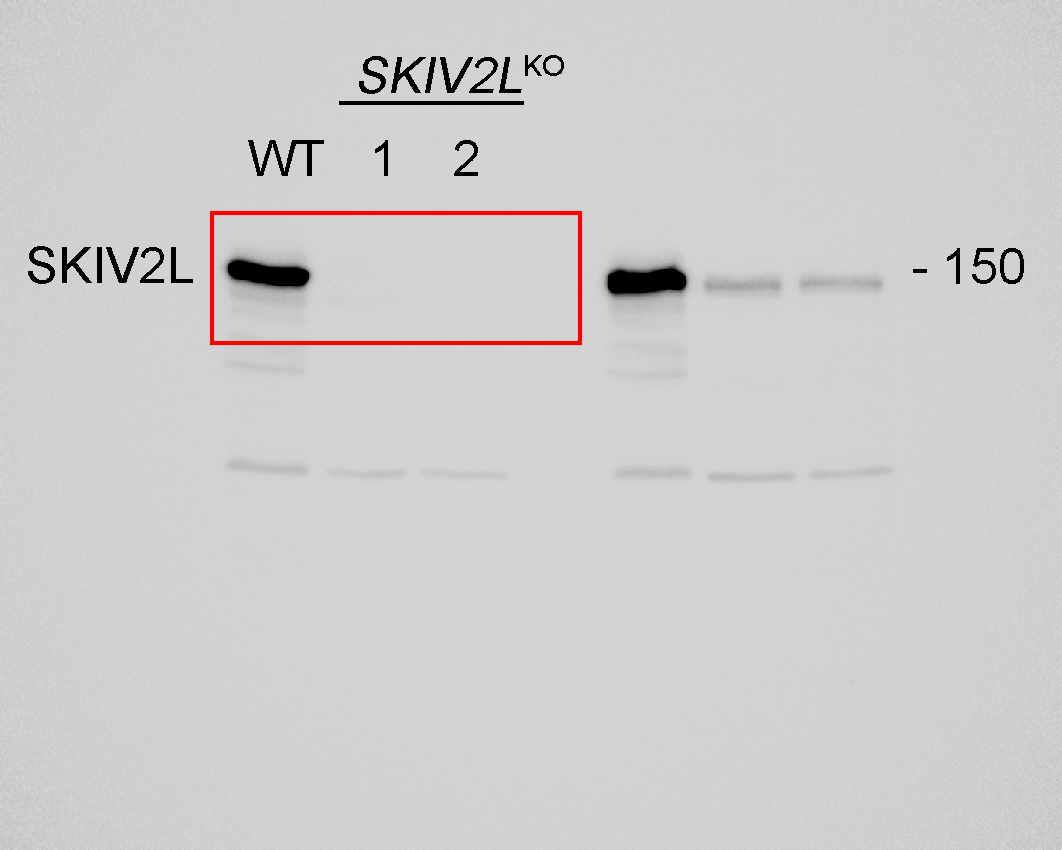

Supplement: Supplementary file 2 — Source data Fig. 1 [file 44318_2024_187_MOESM2_ESM.zip › Figure1/1A/SKIV2L.tif]

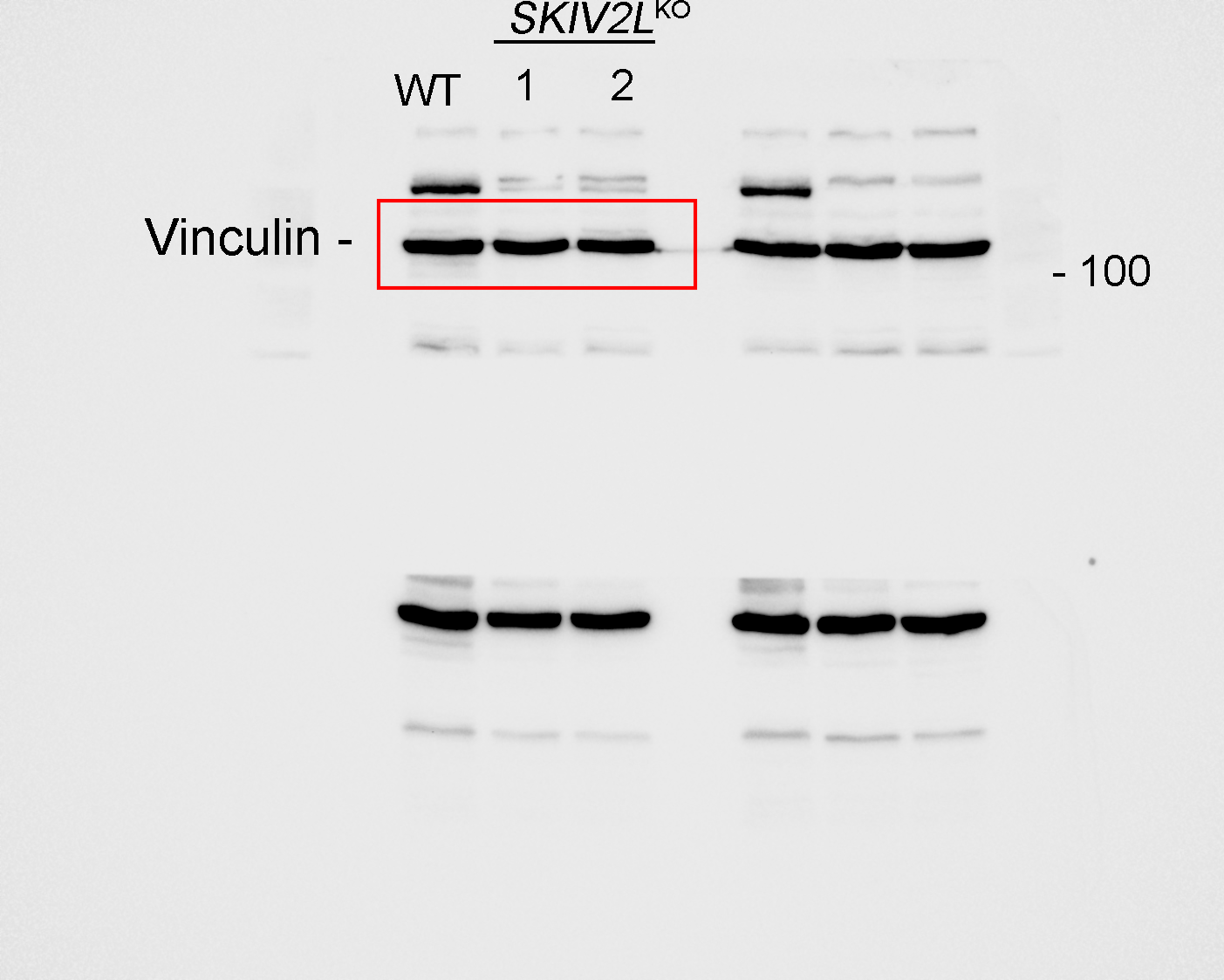

Supplement: Supplementary file 2 — Source data Fig. 1 [file 44318_2024_187_MOESM2_ESM.zip › Figure1/1A/Vinculin.tif]

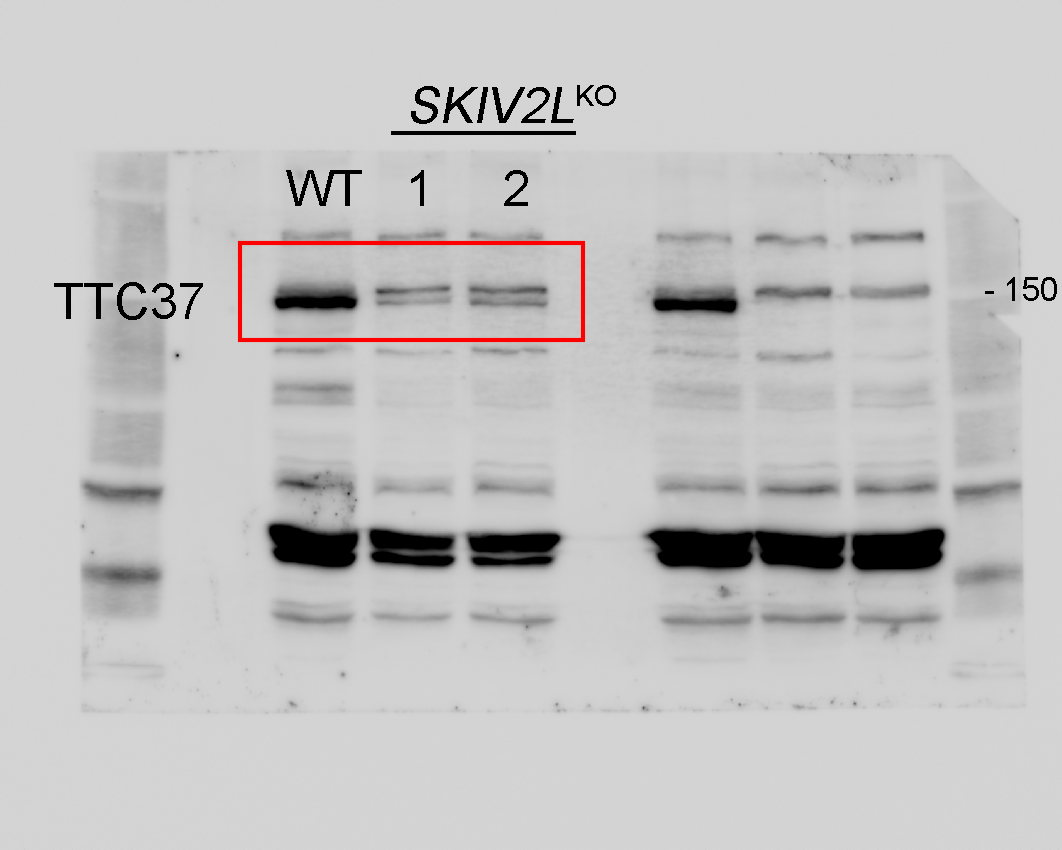

Supplement: Supplementary file 2 — Source data Fig. 1 [file 44318_2024_187_MOESM2_ESM.zip › Figure1/1A/TTC37.tif]

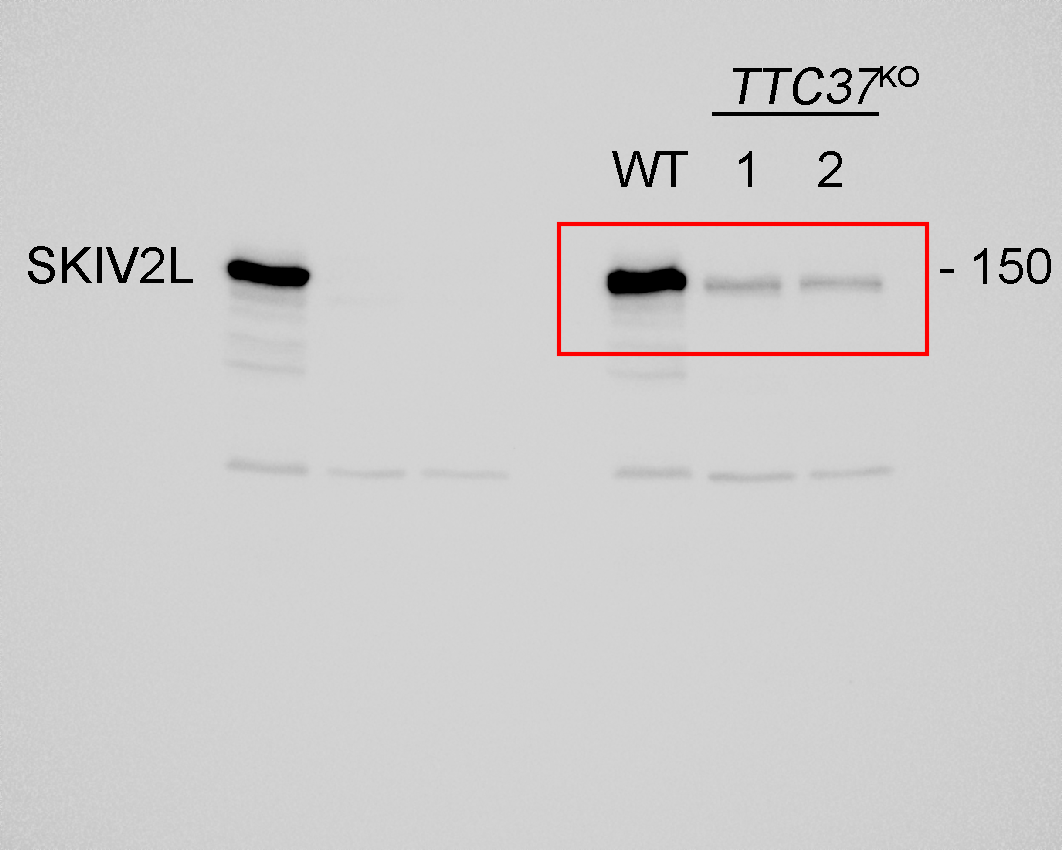

Supplement: Supplementary file 2 — Source data Fig. 1 [file 44318_2024_187_MOESM2_ESM.zip › Figure1/1E/SKIV2L.tif]

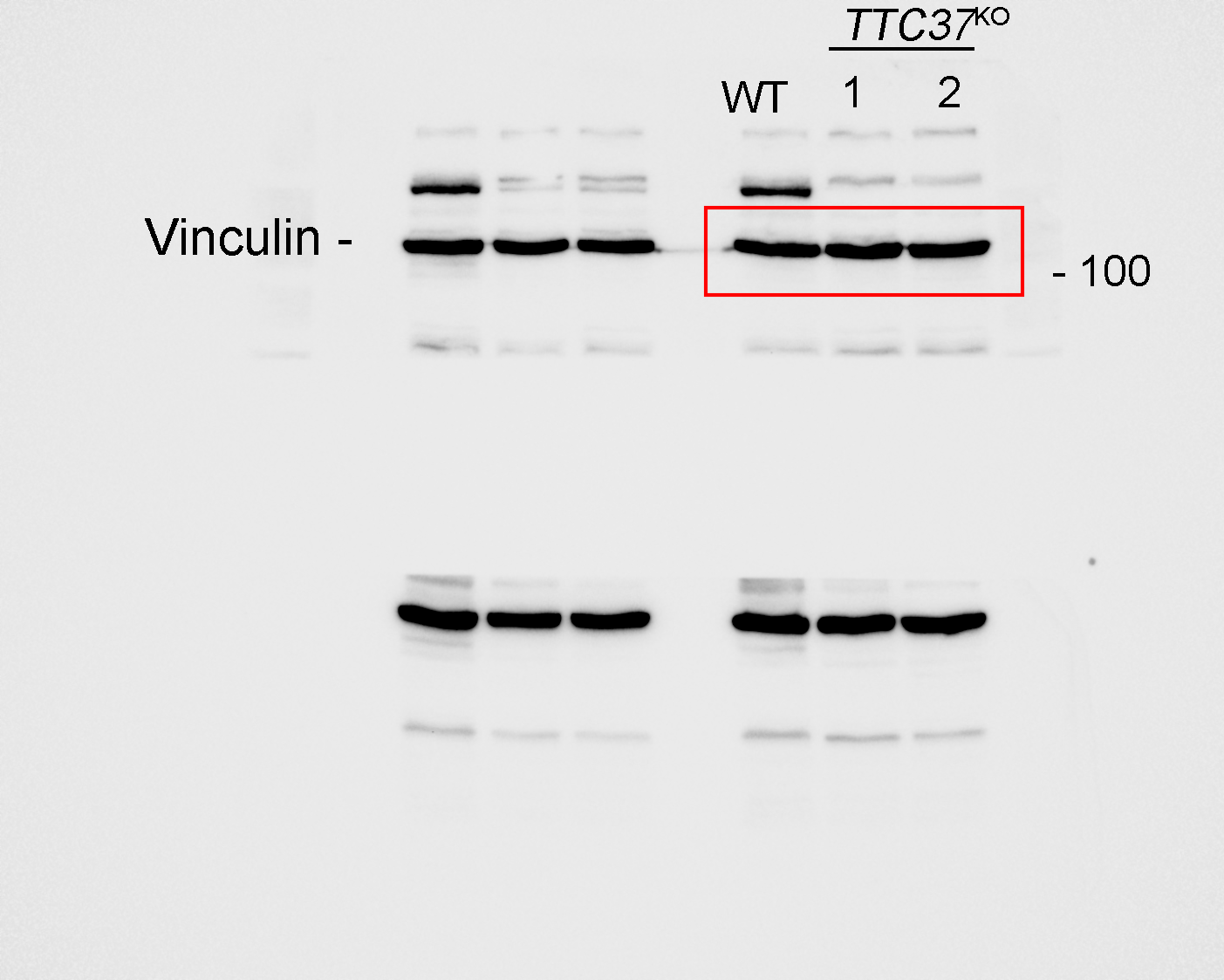

Supplement: Supplementary file 2 — Source data Fig. 1 [file 44318_2024_187_MOESM2_ESM.zip › Figure1/1E/Vinculin.tif]

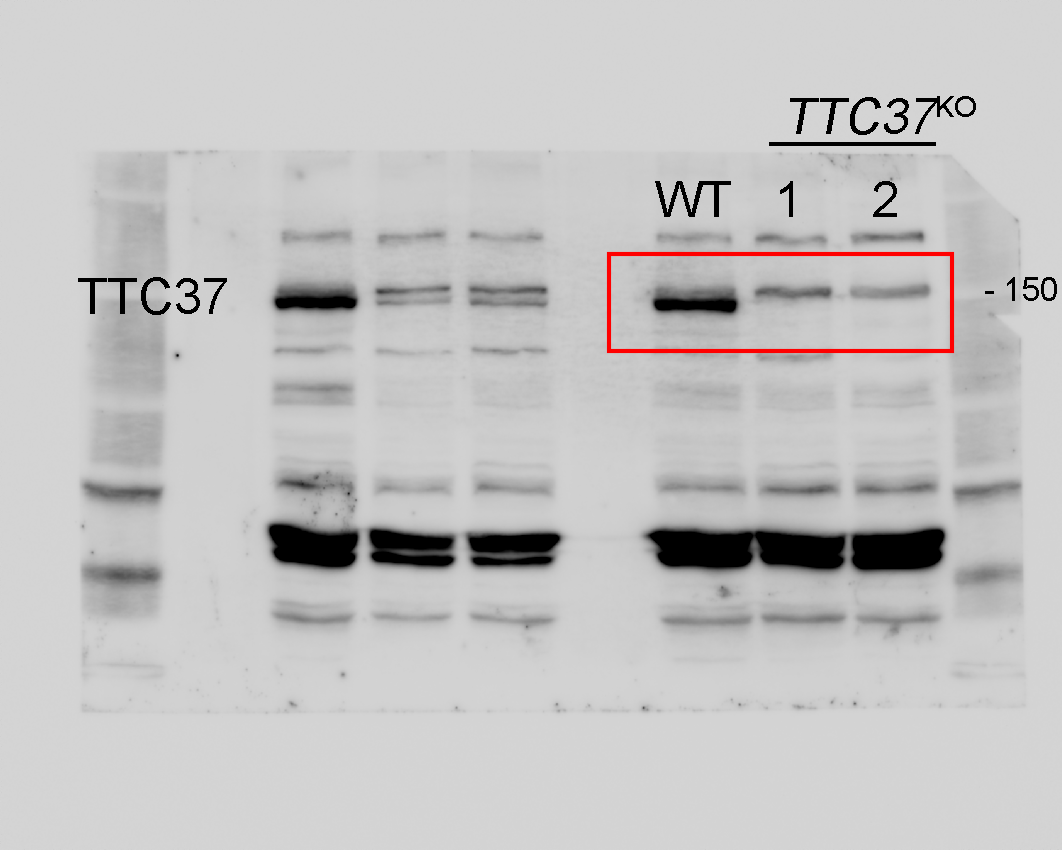

Supplement: Supplementary file 2 — Source data Fig. 1 [file 44318_2024_187_MOESM2_ESM.zip › Figure1/1E/TTC37.tif]

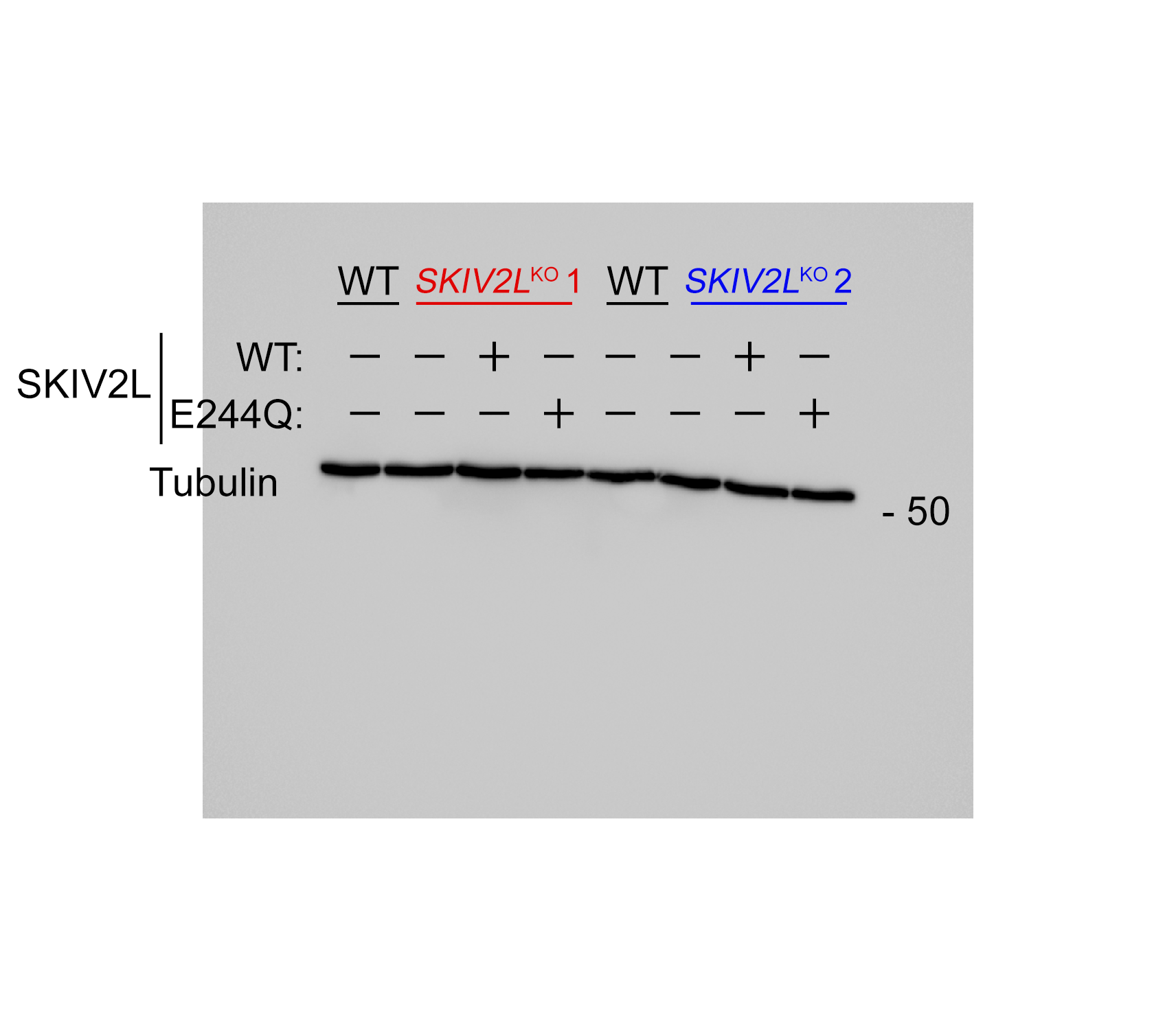

Supplement: Supplementary file 2 — Source data Fig. 1 [file 44318_2024_187_MOESM2_ESM.zip › Figure1/1C/Tubulin.tif]

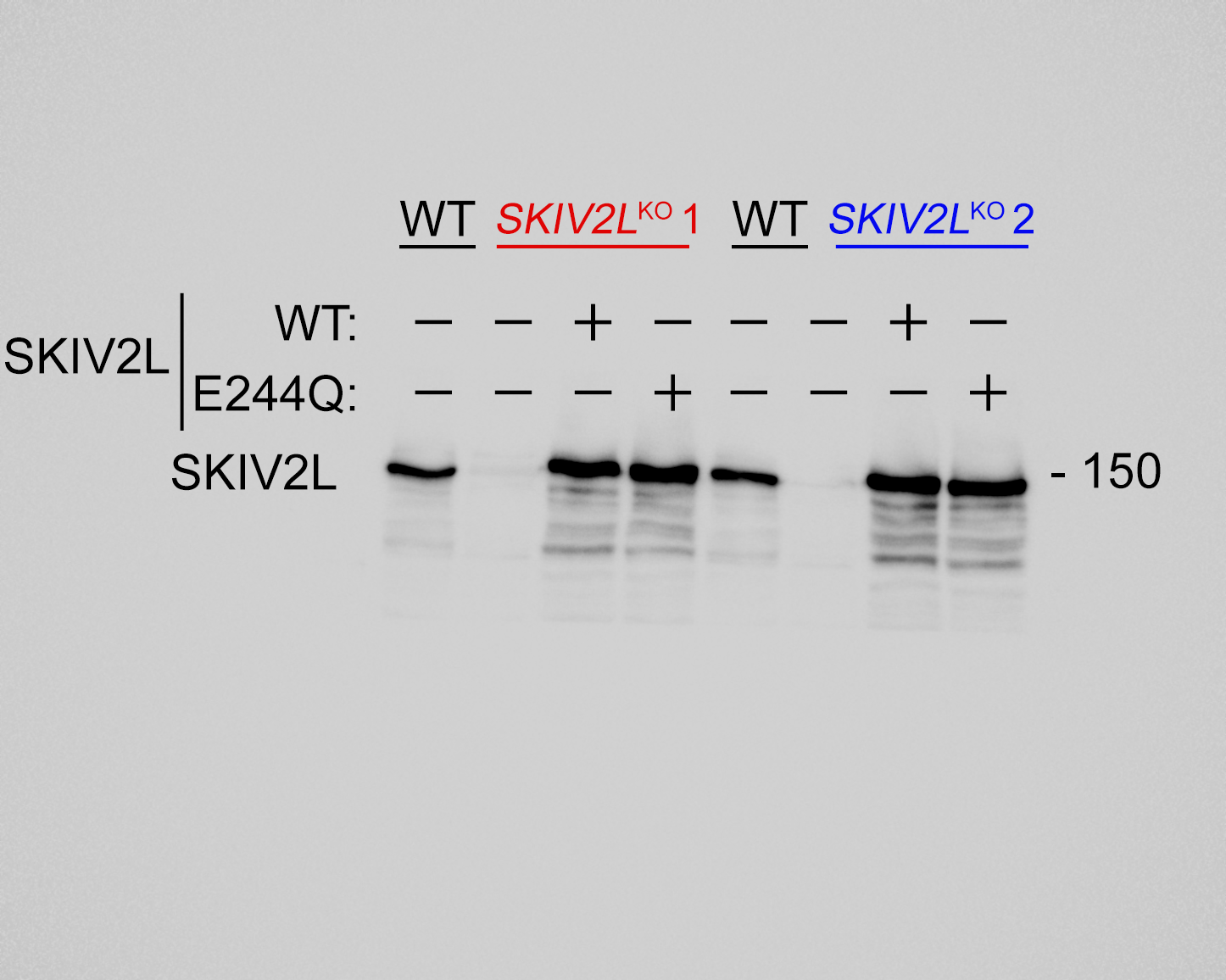

Supplement: Supplementary file 2 — Source data Fig. 1 [file 44318_2024_187_MOESM2_ESM.zip › Figure1/1C/SKIV2L.tif]

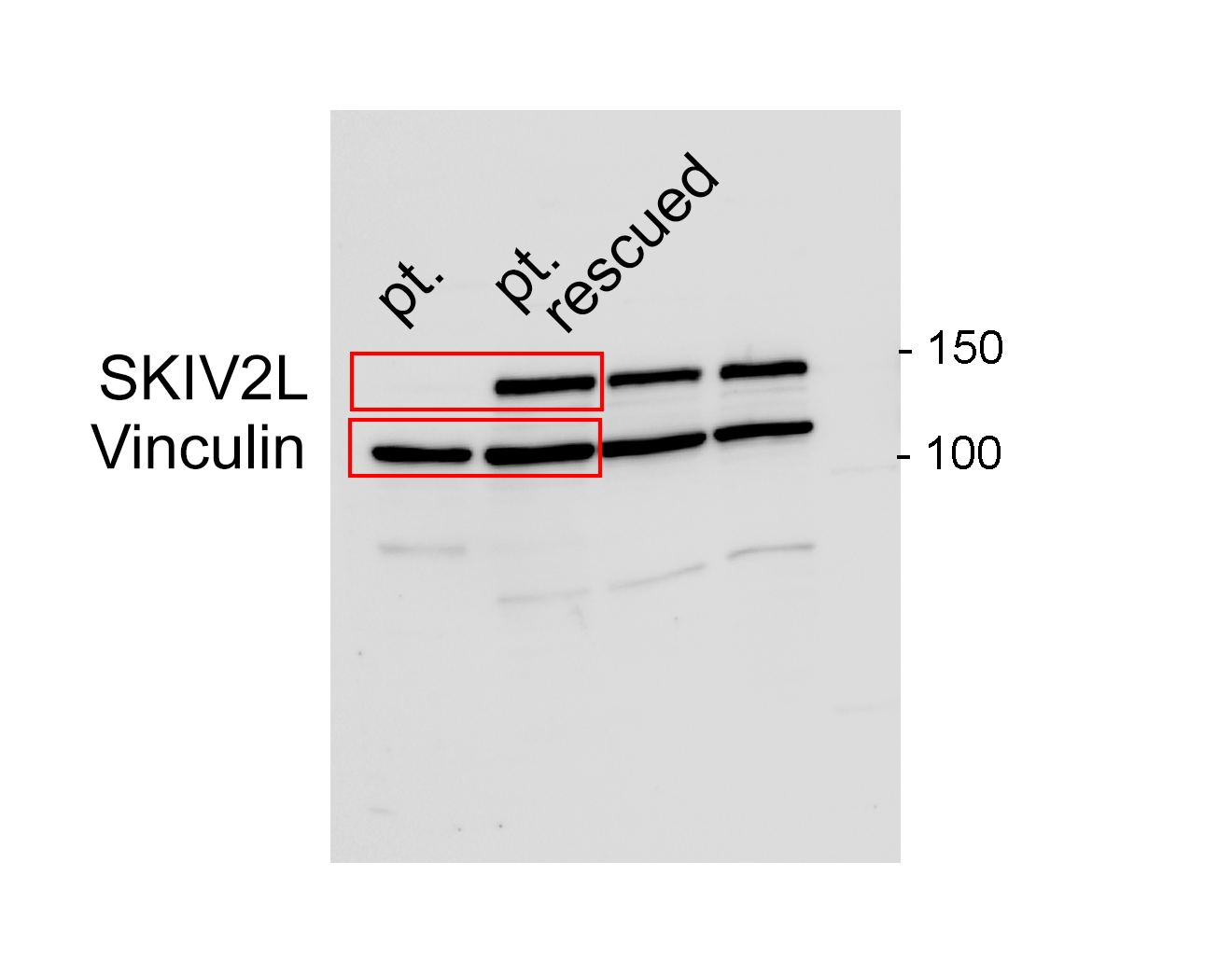

Supplement: Supplementary file 2 — Source data Fig. 1 [file 44318_2024_187_MOESM2_ESM.zip › Figure1/1J/SKIV2L Vinculin.tif]

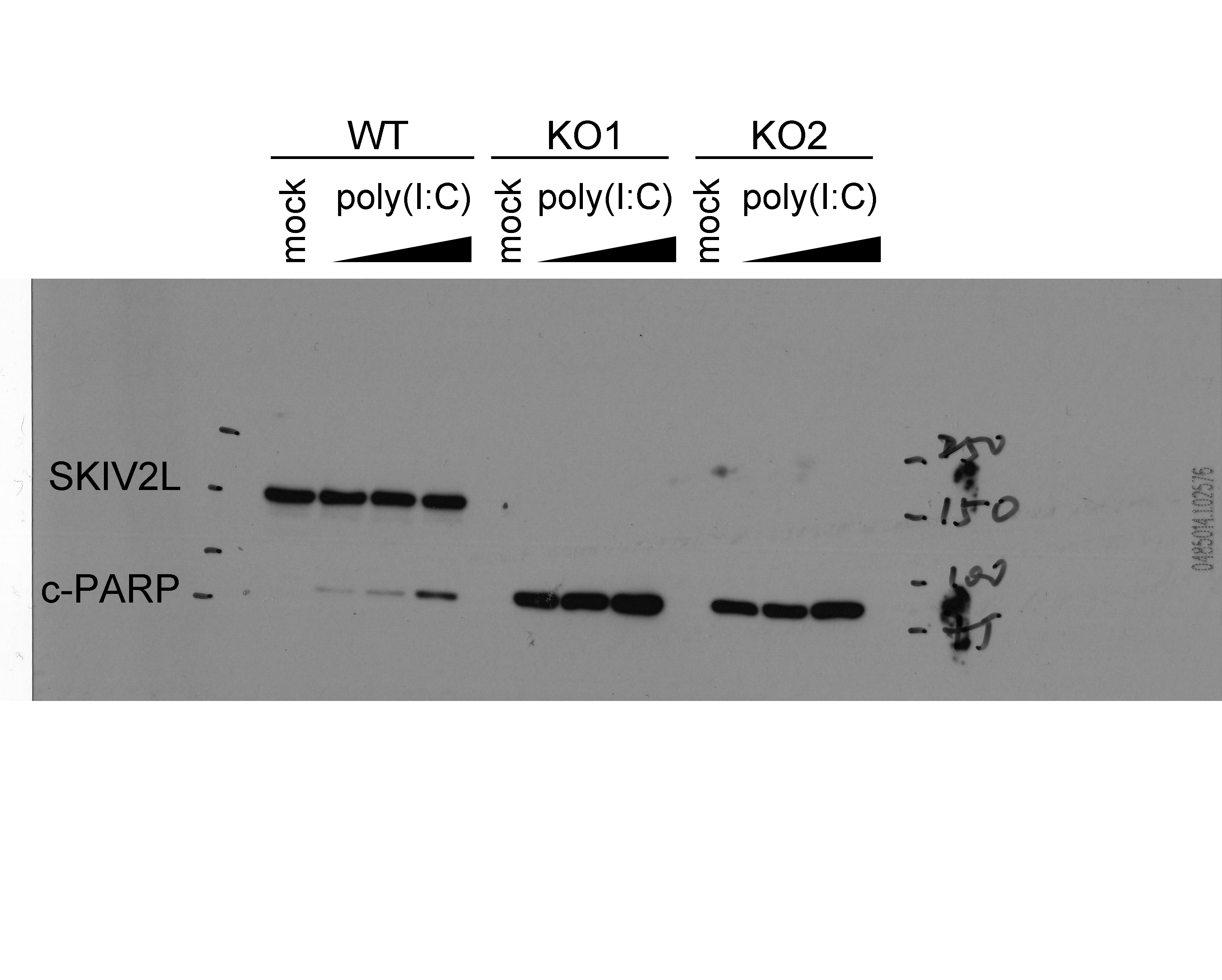

Supplement: Supplementary file 3 — Source data Fig. 2 [file 44318_2024_187_MOESM3_ESM.zip › Figure2/2A/SKIV2L c-PARP.tif]

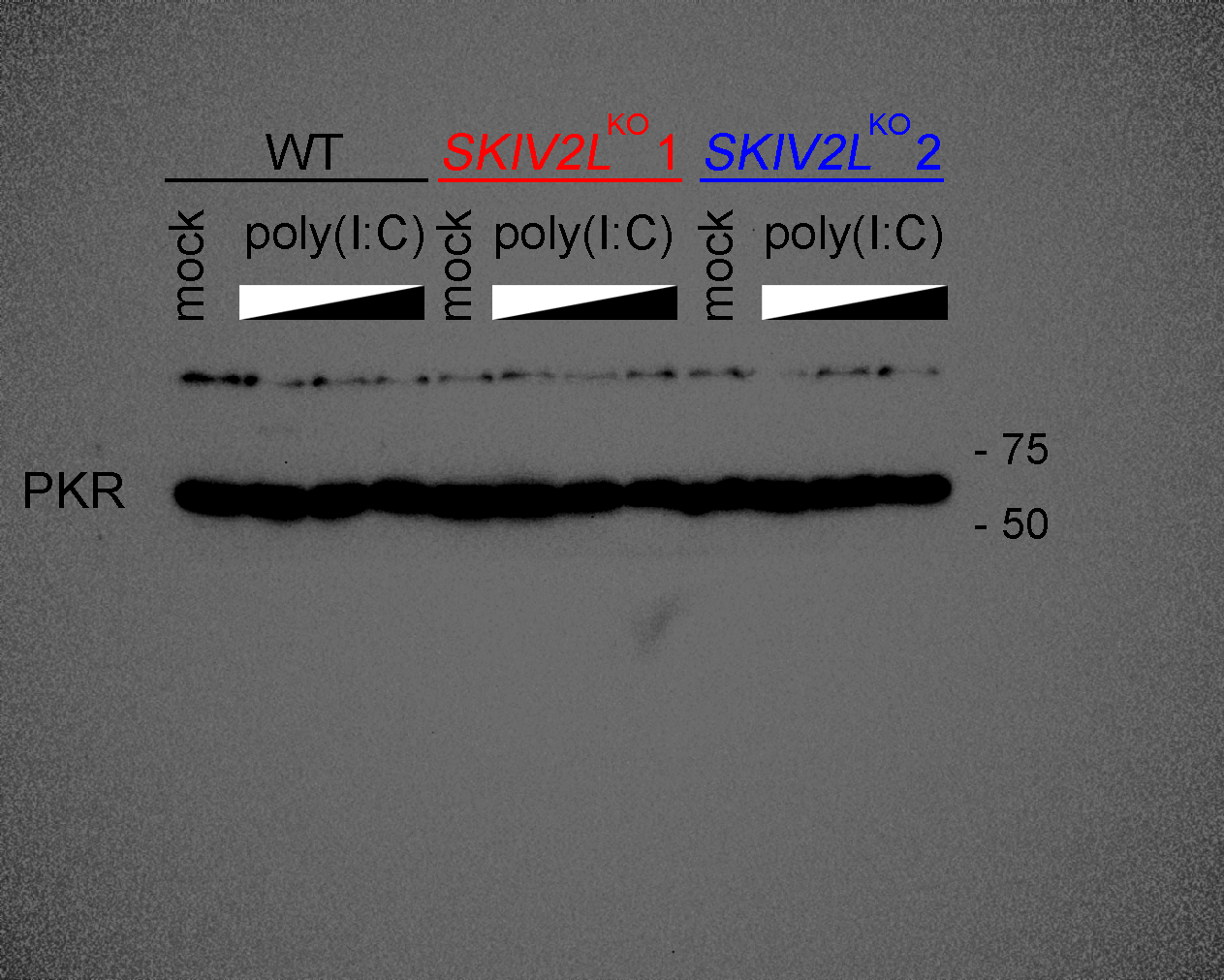

Supplement: Supplementary file 3 — Source data Fig. 2 [file 44318_2024_187_MOESM3_ESM.zip › Figure2/2A/PKR.tif]

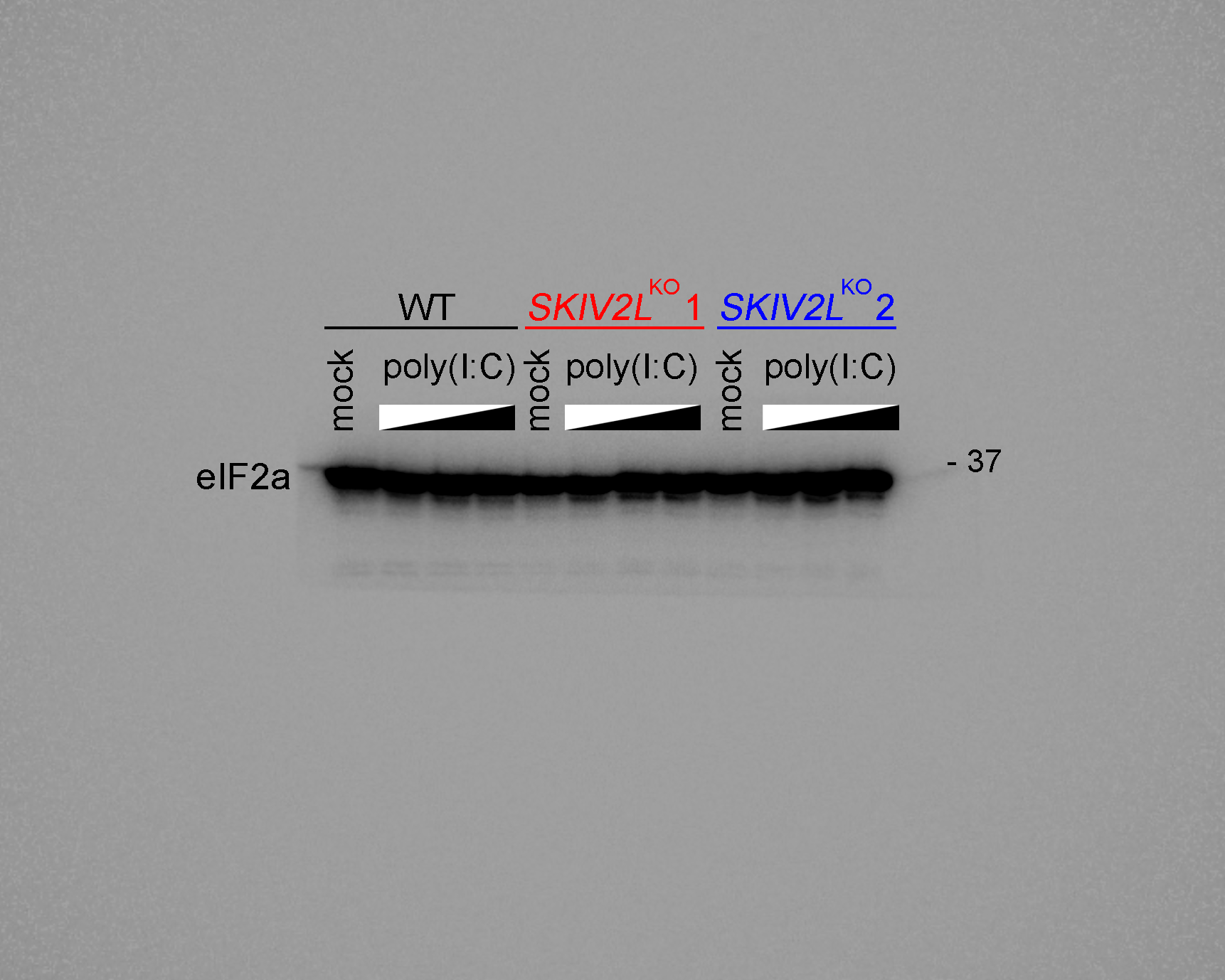

Supplement: Supplementary file 3 — Source data Fig. 2 [file 44318_2024_187_MOESM3_ESM.zip › Figure2/2A/eIF2a.tif]

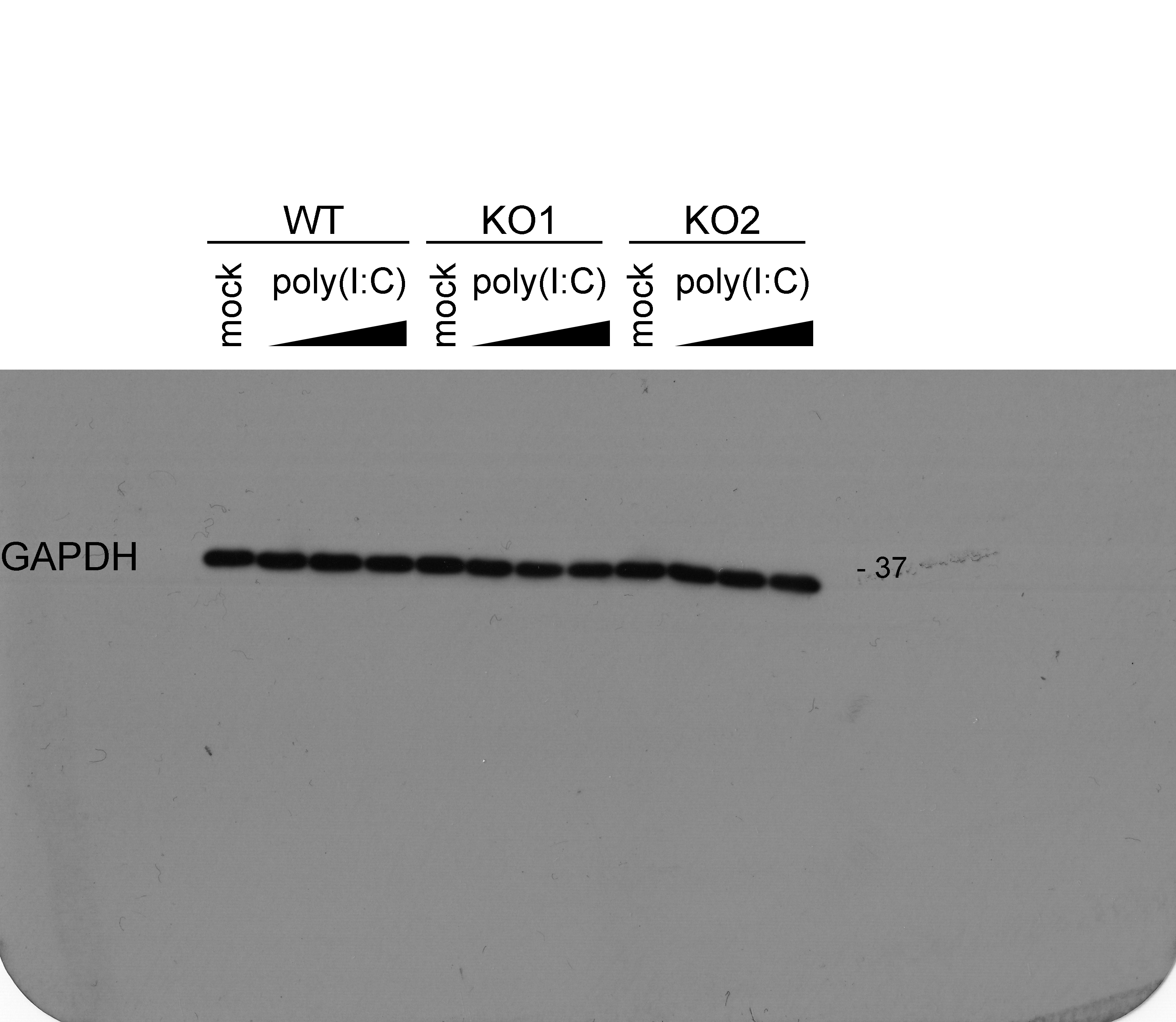

Supplement: Supplementary file 3 — Source data Fig. 2 [file 44318_2024_187_MOESM3_ESM.zip › Figure2/2A/GAPDH.tif]

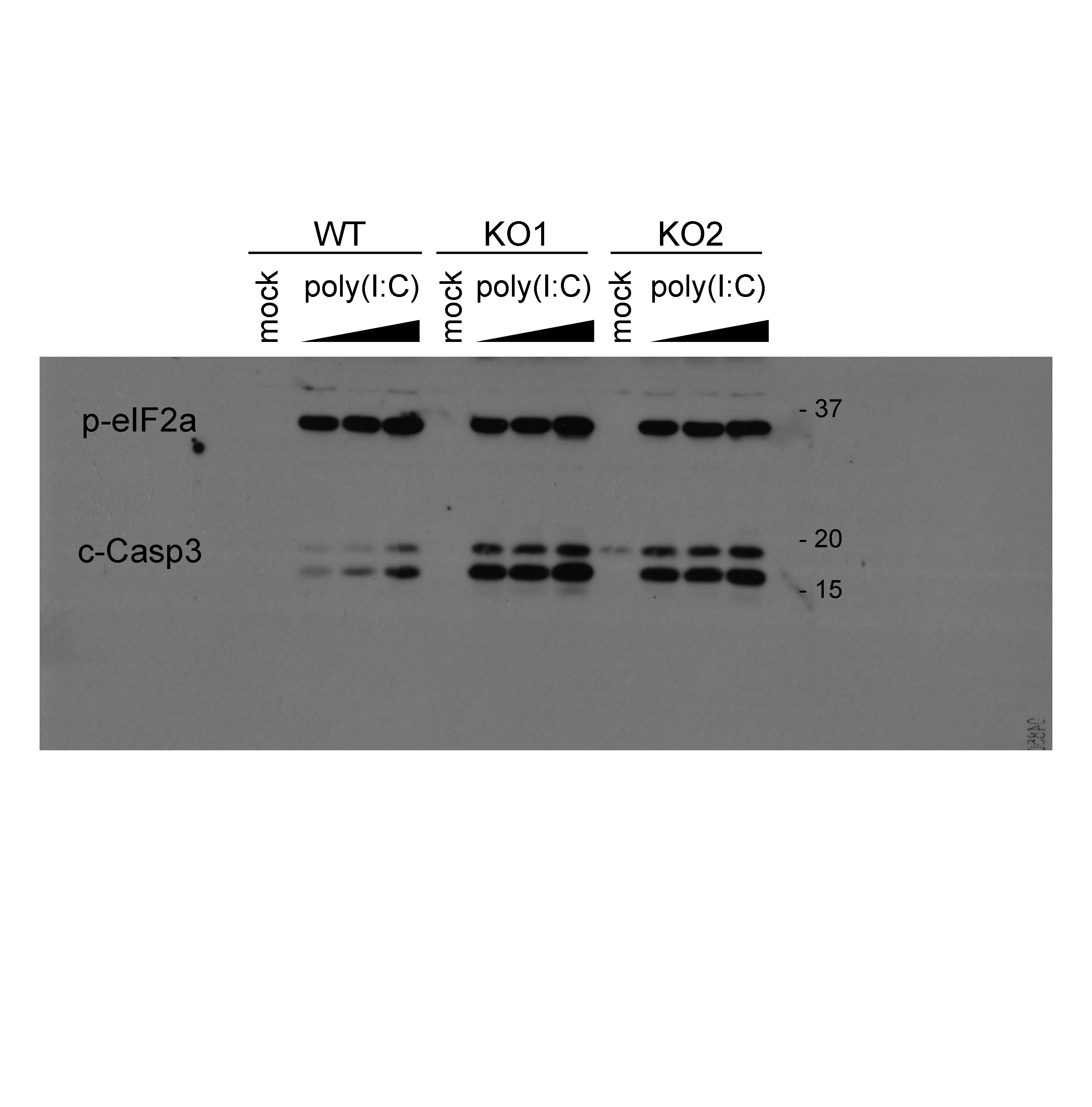

Supplement: Supplementary file 3 — Source data Fig. 2 [file 44318_2024_187_MOESM3_ESM.zip › Figure2/2A/p-eIF2a c-Casp3.tif]

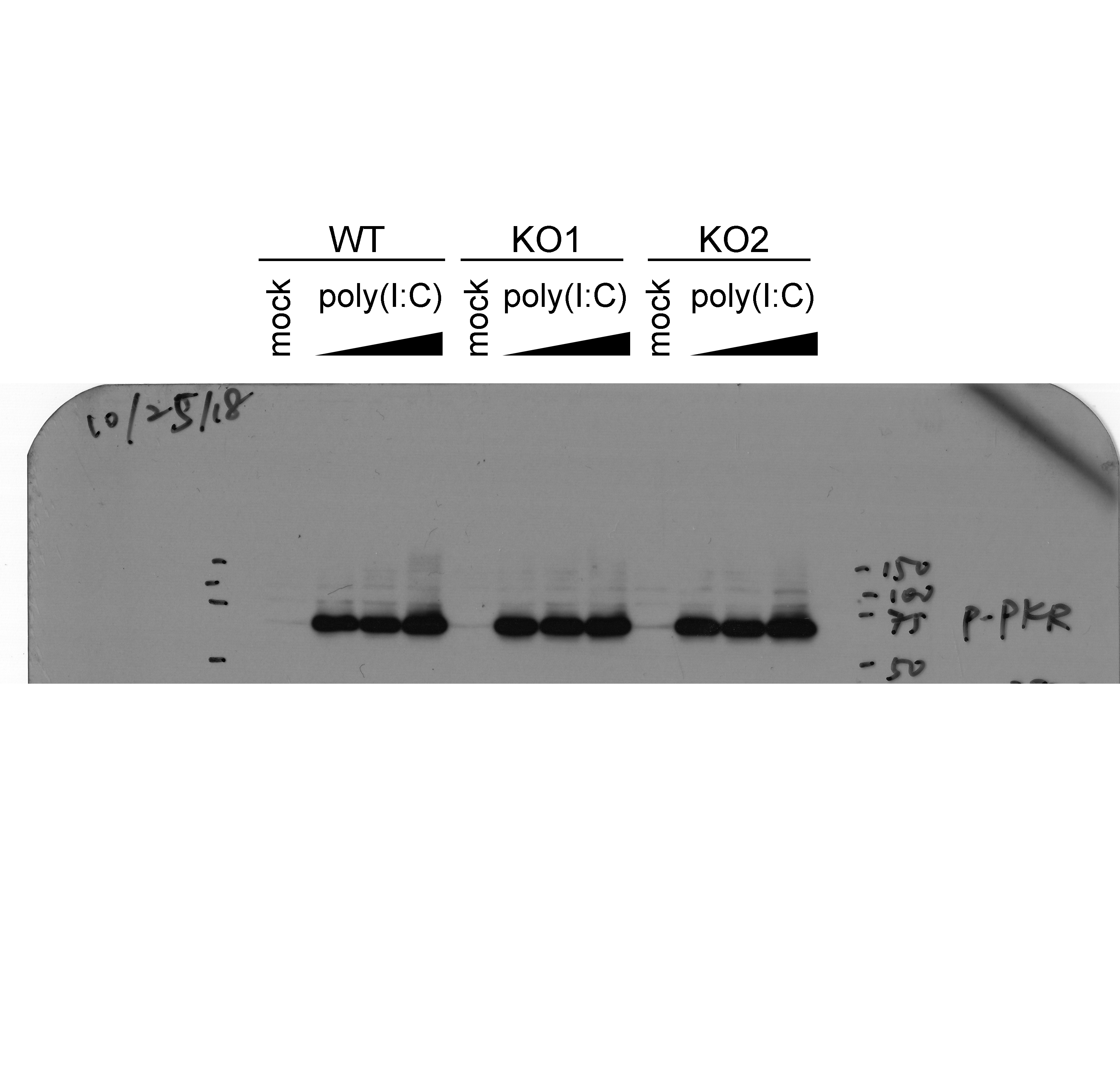

Supplement: Supplementary file 3 — Source data Fig. 2 [file 44318_2024_187_MOESM3_ESM.zip › Figure2/2A/p-PKR.tif]

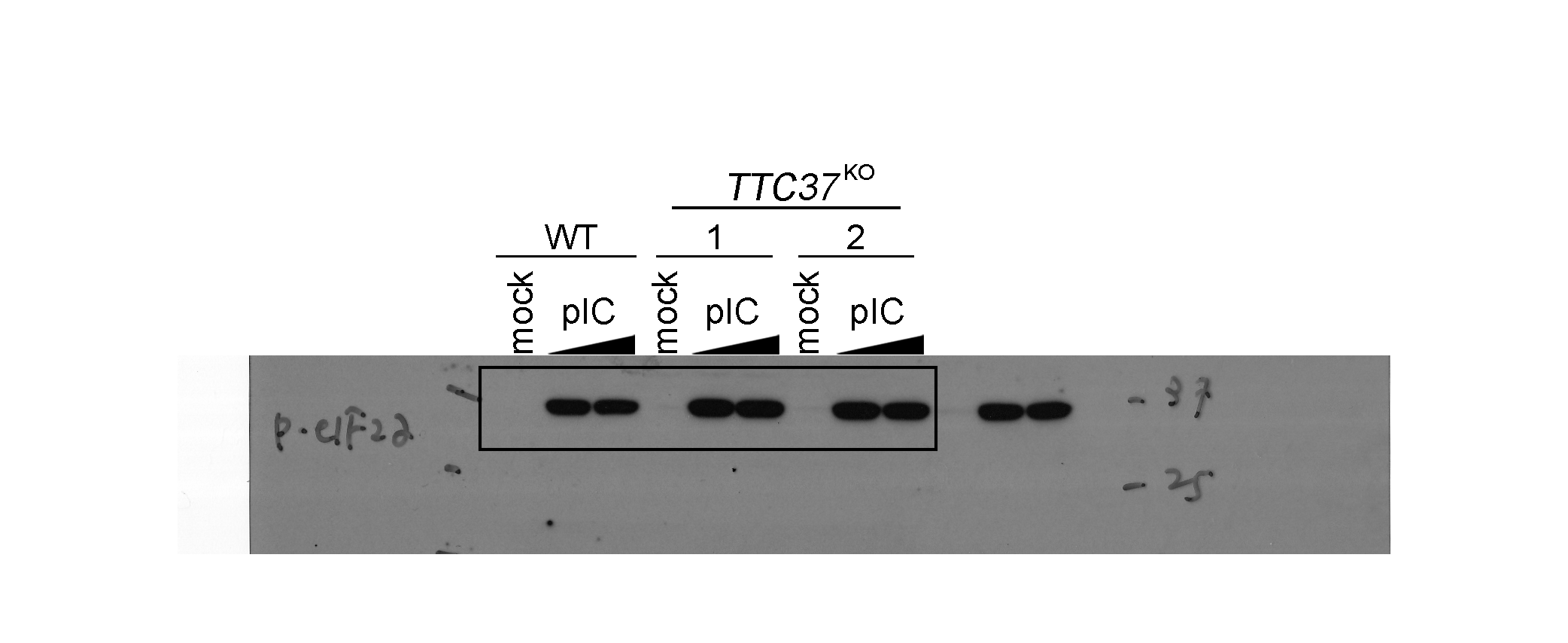

Supplement: Supplementary file 3 — Source data Fig. 2 [file 44318_2024_187_MOESM3_ESM.zip › Figure2/2F/p-eIF2a.tif]

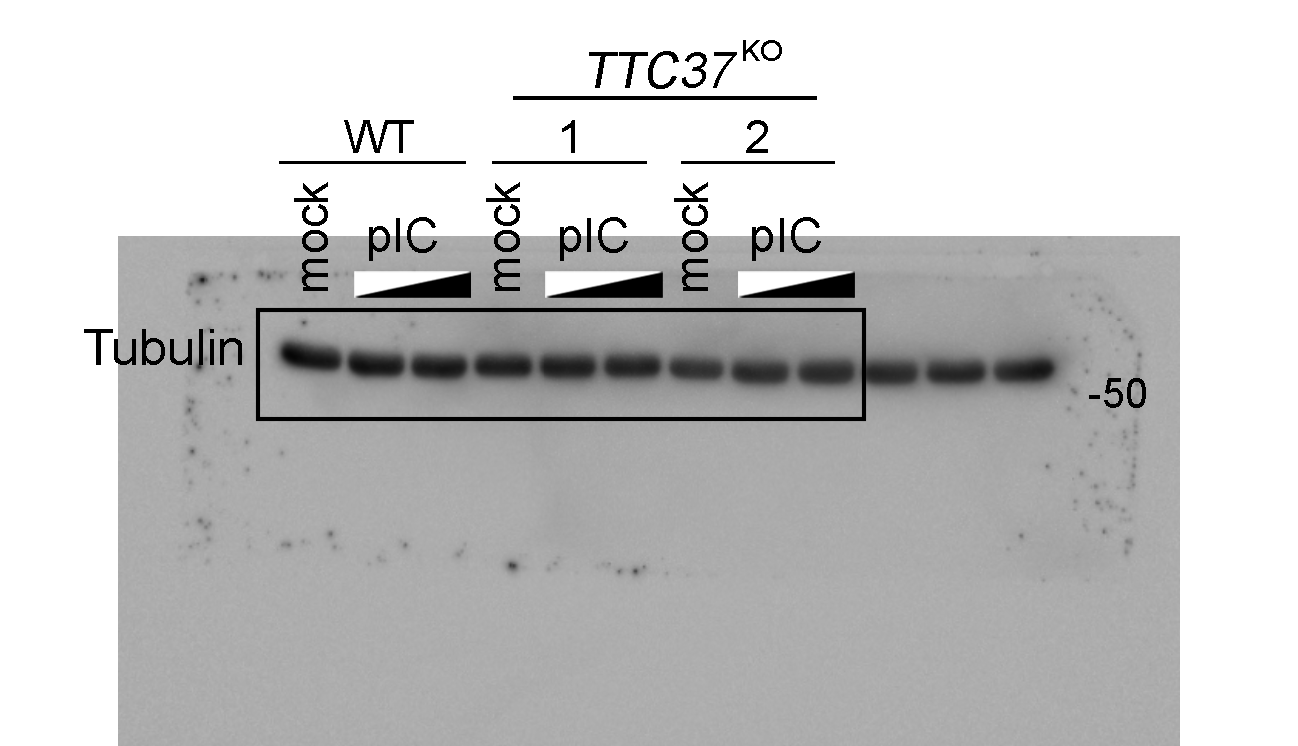

Supplement: Supplementary file 3 — Source data Fig. 2 [file 44318_2024_187_MOESM3_ESM.zip › Figure2/2F/Tubulin.tif]

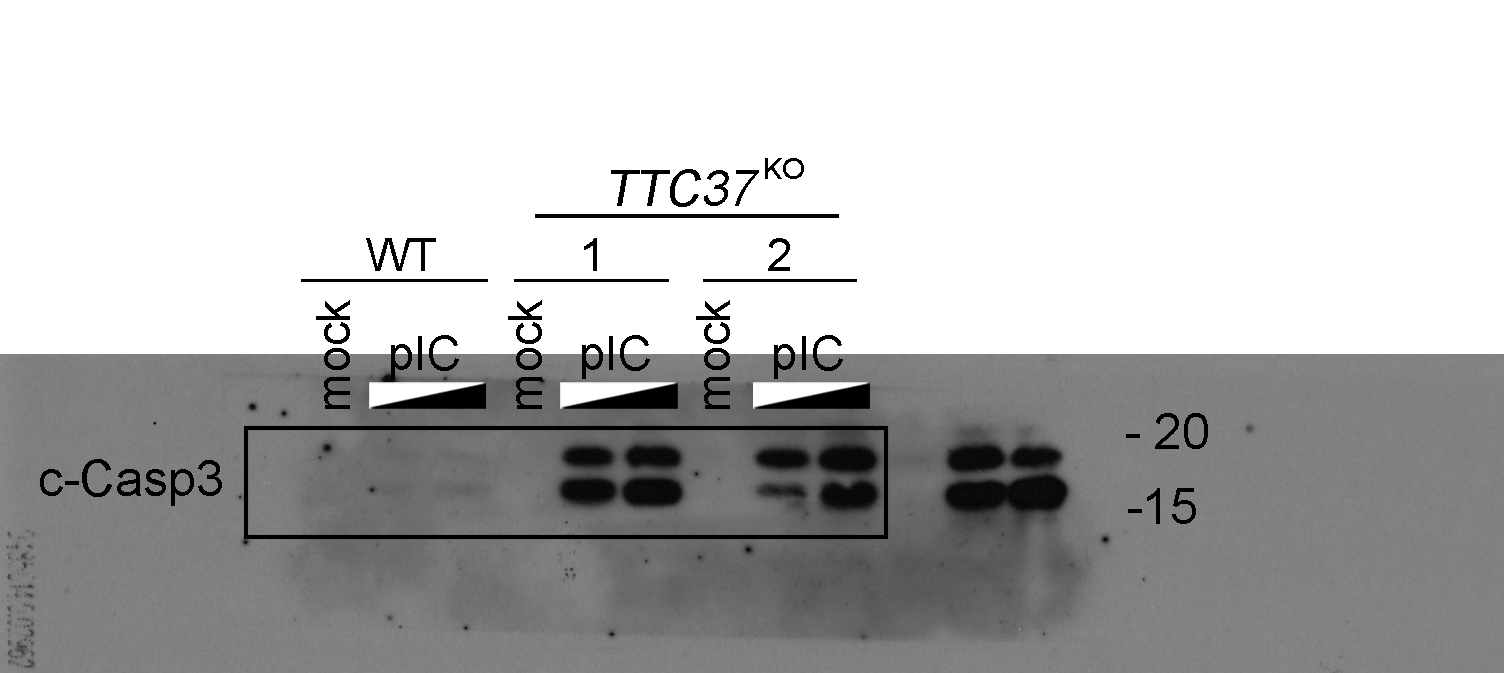

Supplement: Supplementary file 3 — Source data Fig. 2 [file 44318_2024_187_MOESM3_ESM.zip › Figure2/2F/c-Casp3.tif]

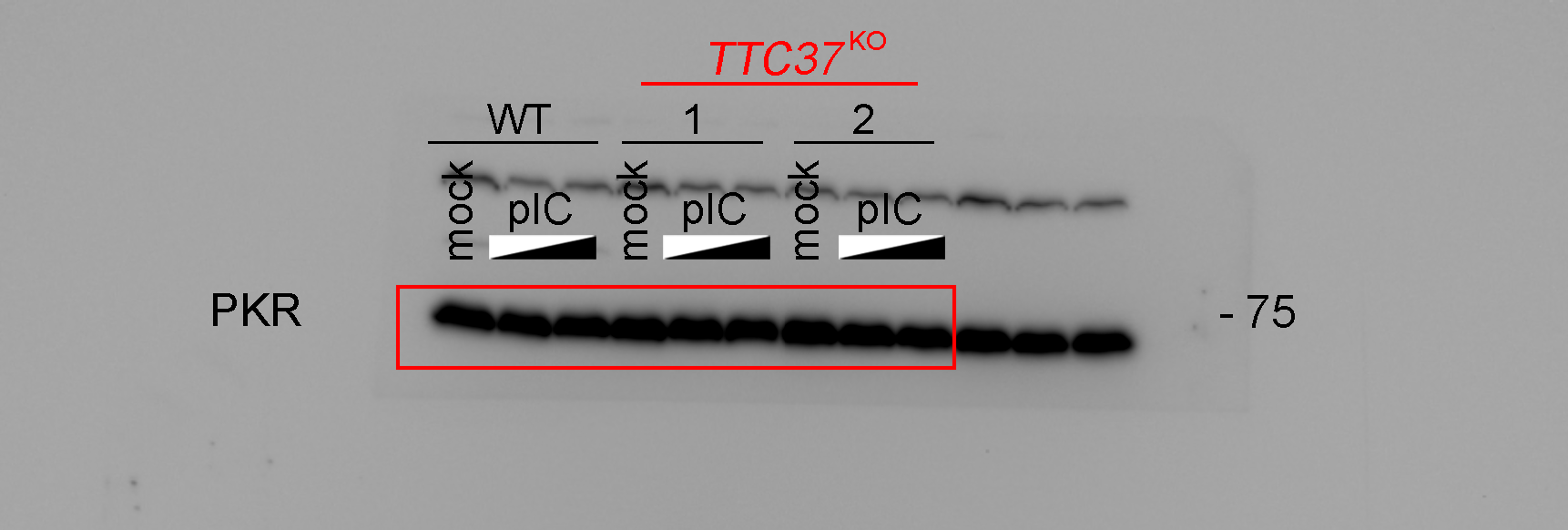

Supplement: Supplementary file 3 — Source data Fig. 2 [file 44318_2024_187_MOESM3_ESM.zip › Figure2/2F/PKR.tif]

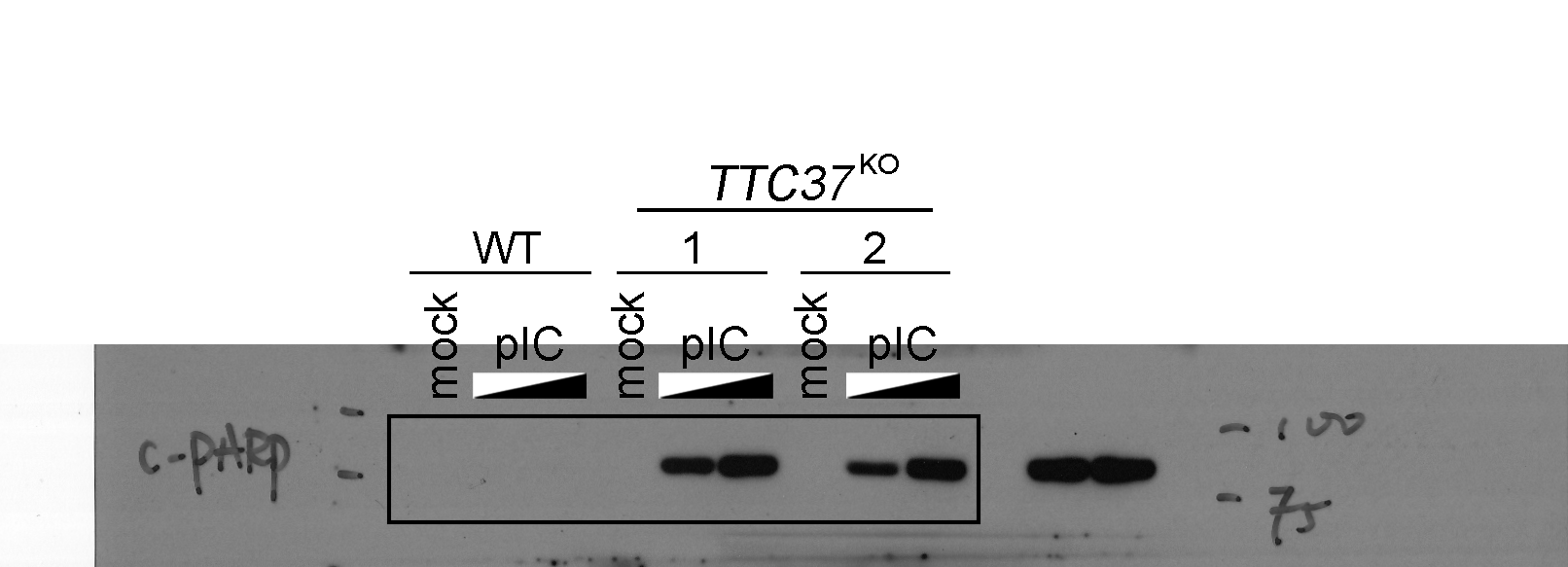

Supplement: Supplementary file 3 — Source data Fig. 2 [file 44318_2024_187_MOESM3_ESM.zip › Figure2/2F/c-PARP.tif]

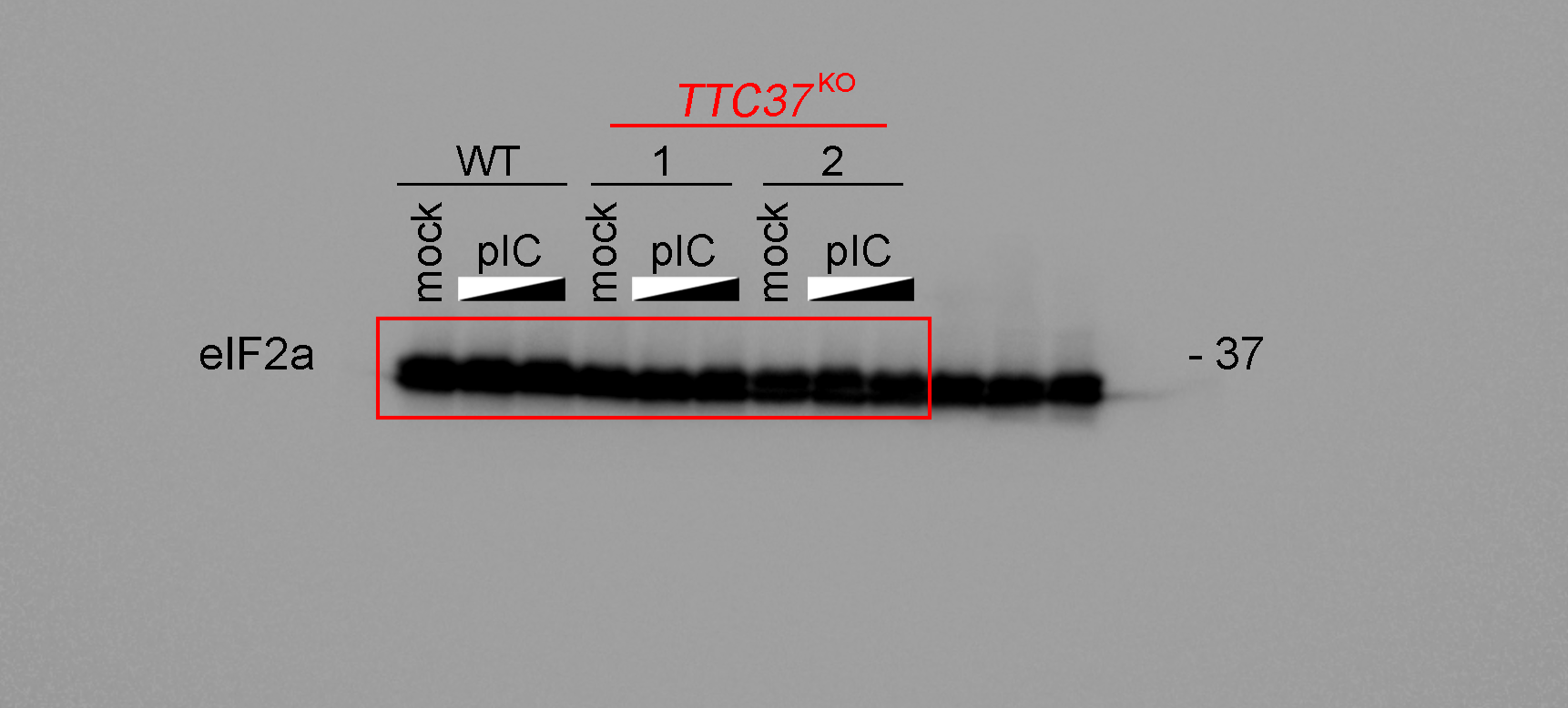

Supplement: Supplementary file 3 — Source data Fig. 2 [file 44318_2024_187_MOESM3_ESM.zip › Figure2/2F/eIF2a.tif]

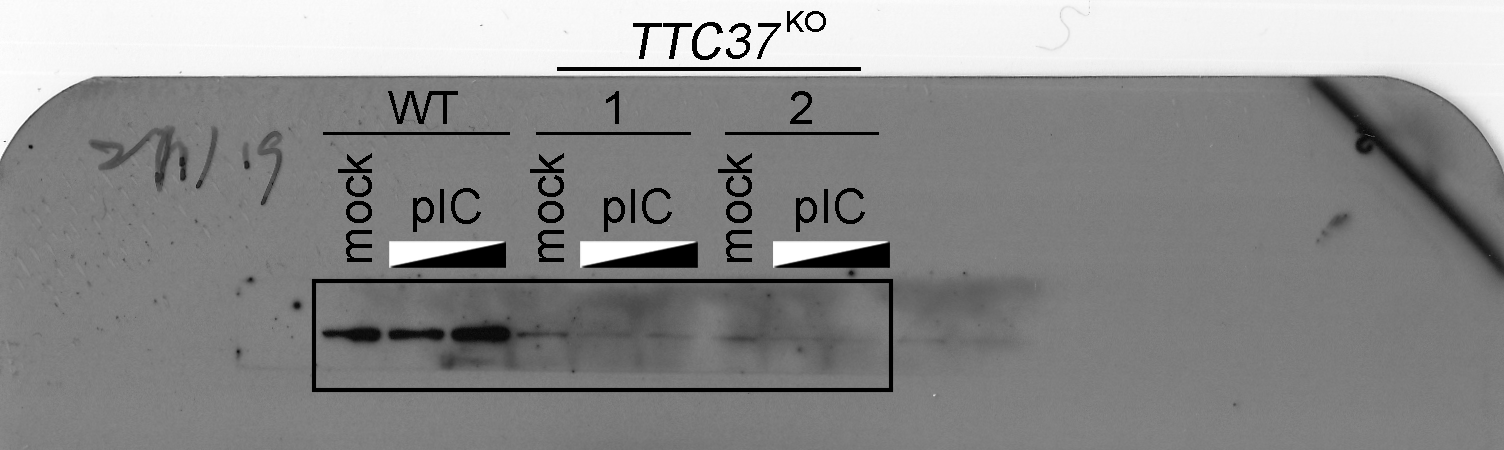

Supplement: Supplementary file 3 — Source data Fig. 2 [file 44318_2024_187_MOESM3_ESM.zip › Figure2/2F/SKIV2L.tif]

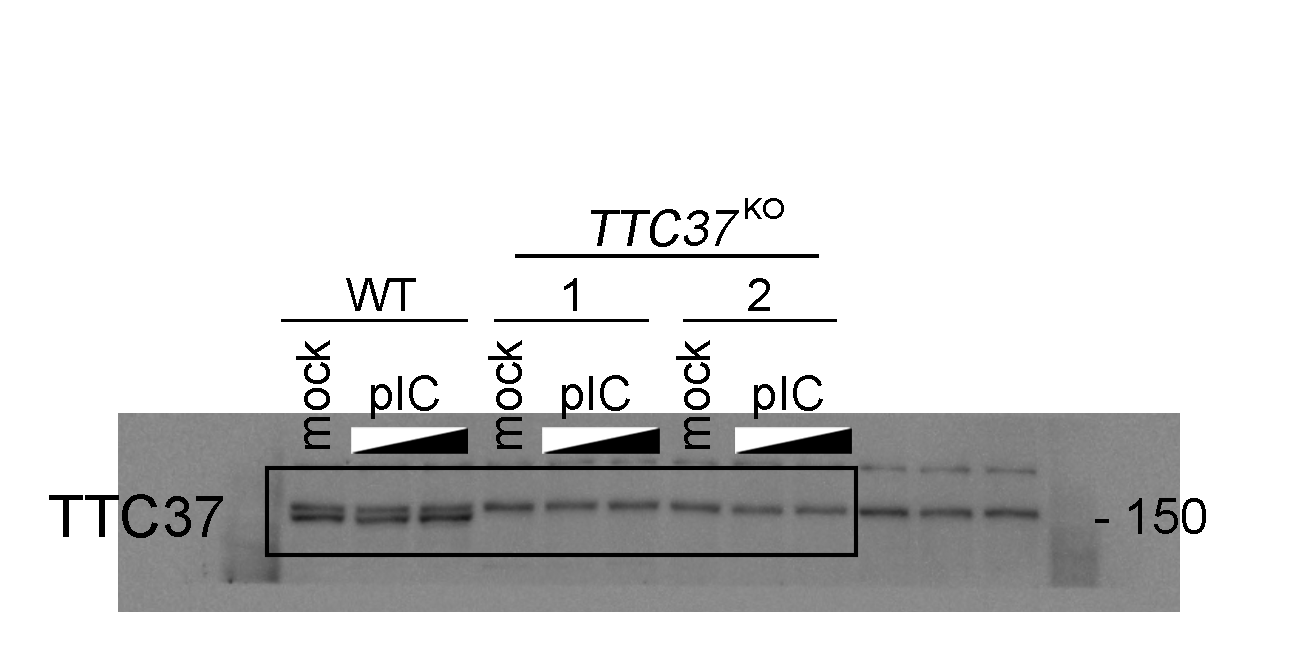

Supplement: Supplementary file 3 — Source data Fig. 2 [file 44318_2024_187_MOESM3_ESM.zip › Figure2/2F/TTC37.tif]

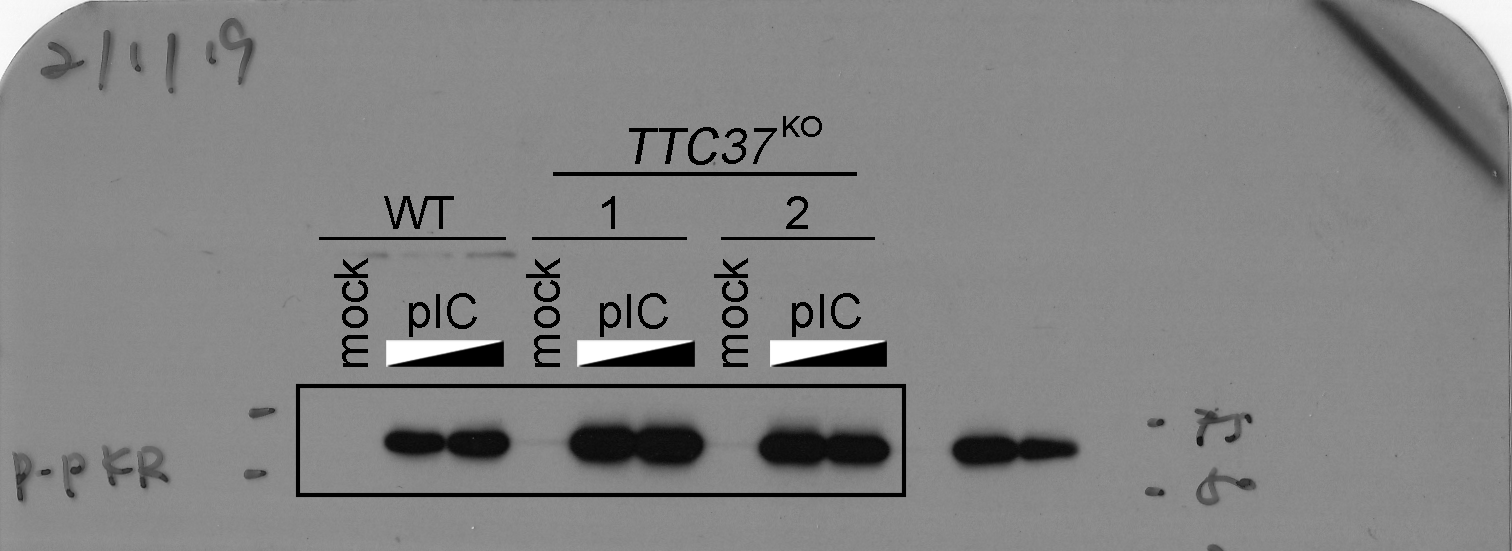

Supplement: Supplementary file 3 — Source data Fig. 2 [file 44318_2024_187_MOESM3_ESM.zip › Figure2/2F/p-PKR.tif]

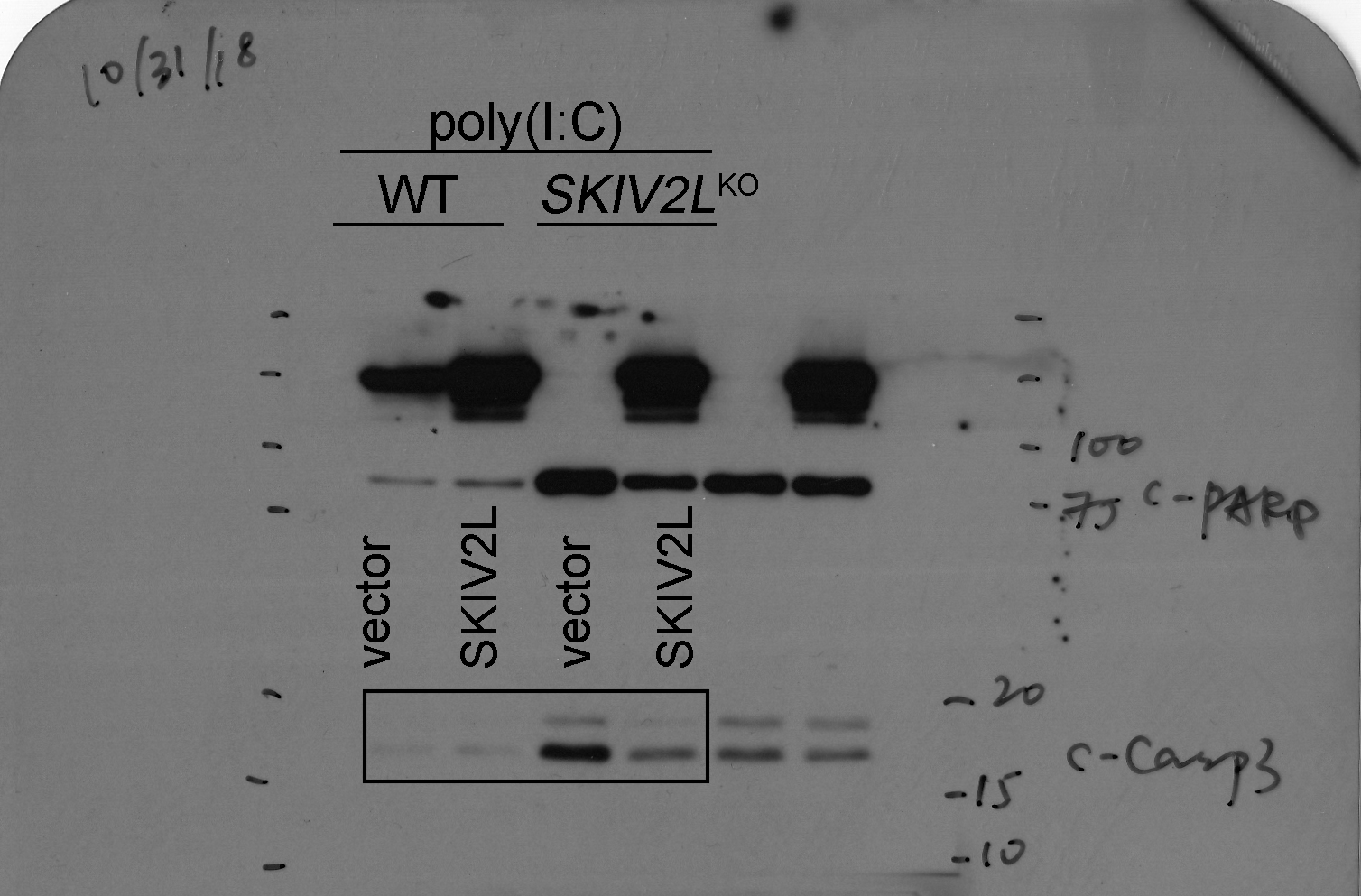

Supplement: Supplementary file 3 — Source data Fig. 2 [file 44318_2024_187_MOESM3_ESM.zip › Figure2/2D/c-Casp3.tif]

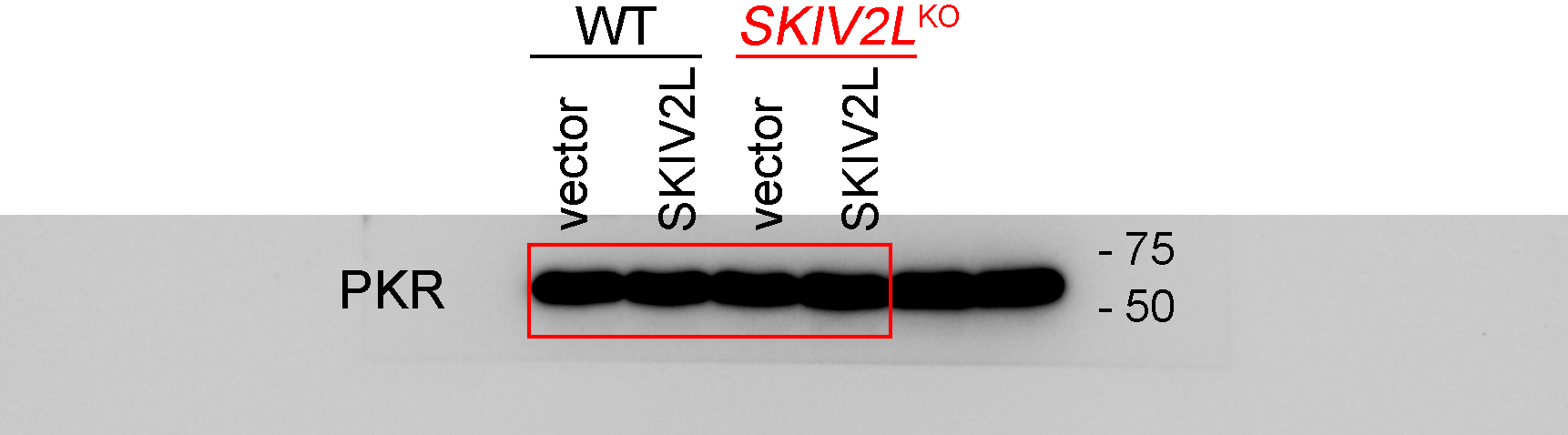

Supplement: Supplementary file 3 — Source data Fig. 2 [file 44318_2024_187_MOESM3_ESM.zip › Figure2/2D/PKR.tif]

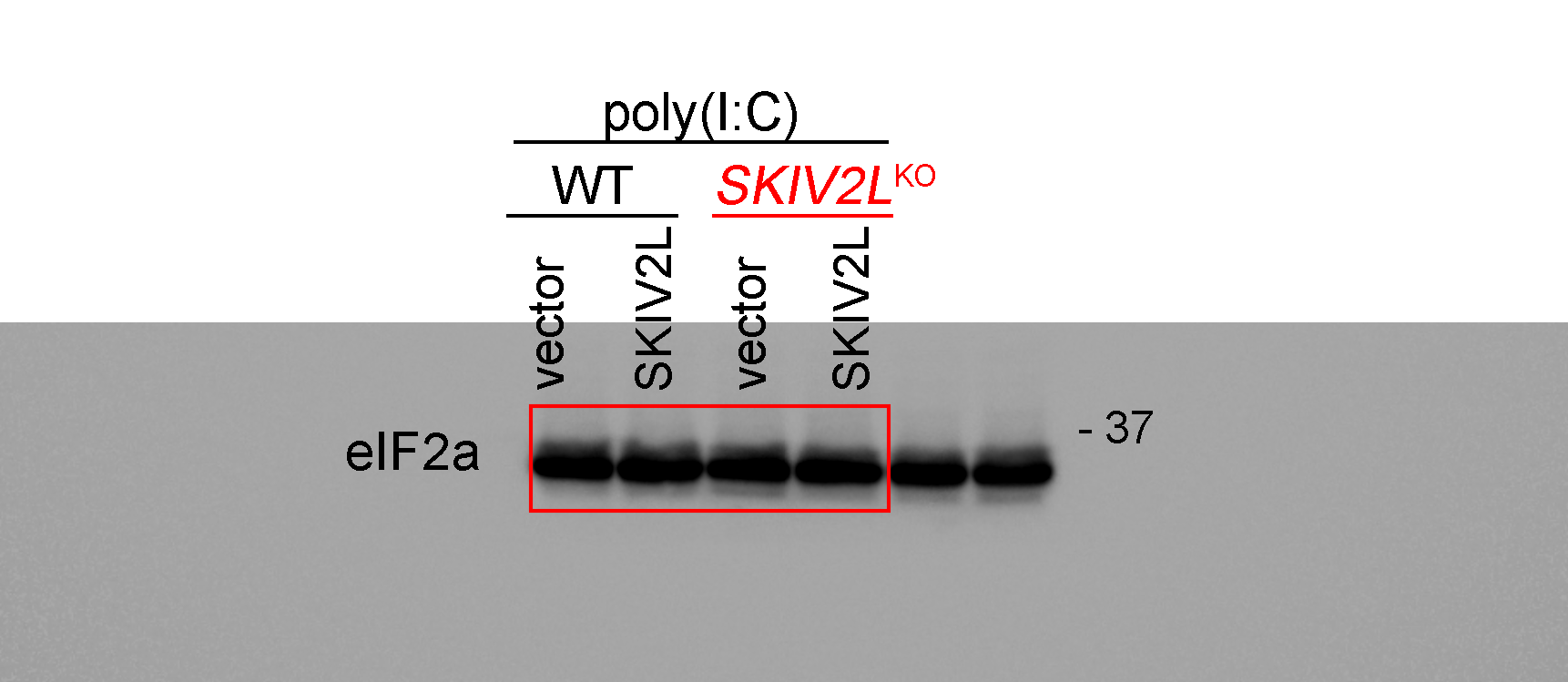

Supplement: Supplementary file 3 — Source data Fig. 2 [file 44318_2024_187_MOESM3_ESM.zip › Figure2/2D/eIF2a.tif]

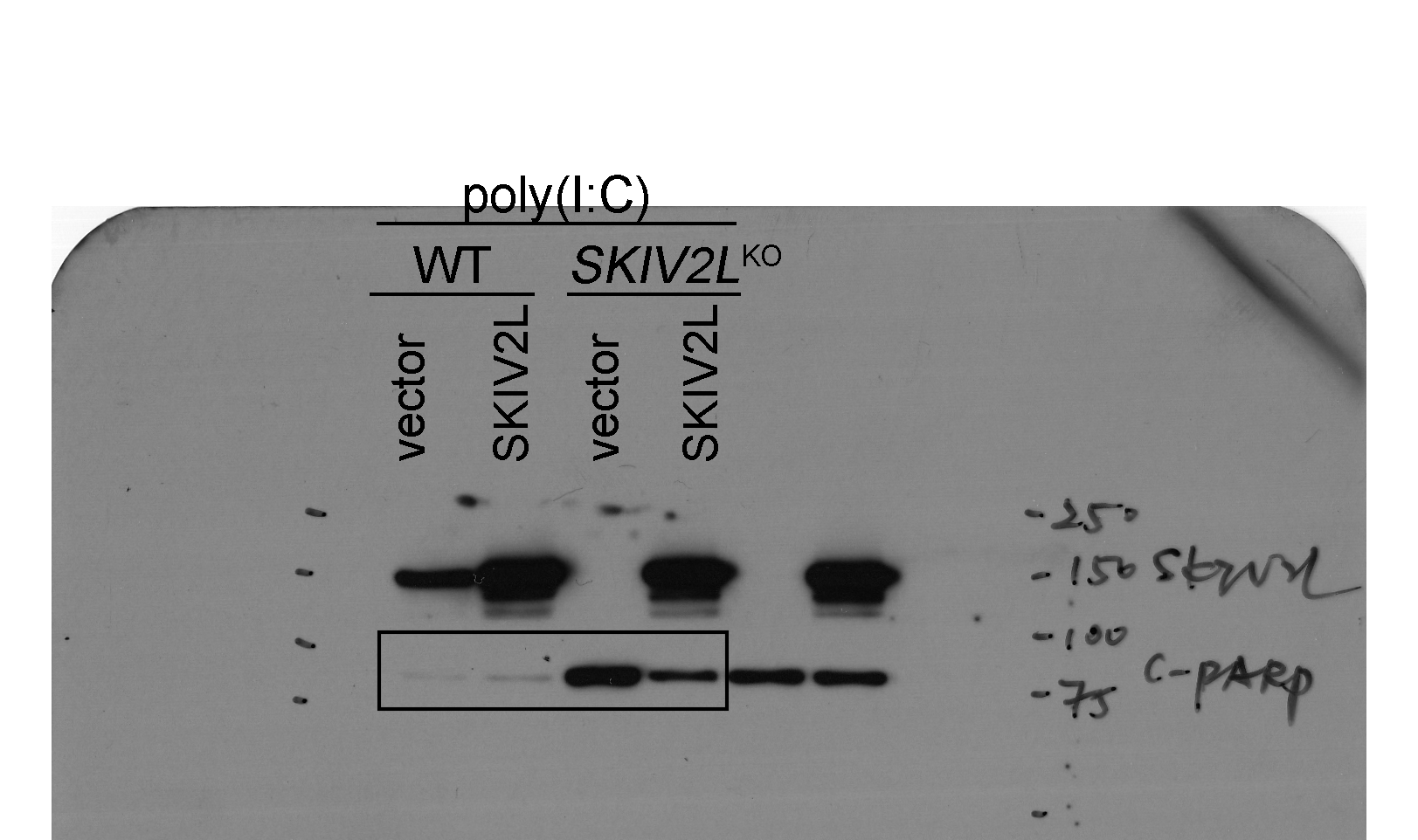

Supplement: Supplementary file 3 — Source data Fig. 2 [file 44318_2024_187_MOESM3_ESM.zip › Figure2/2D/c-PAPR.tif]

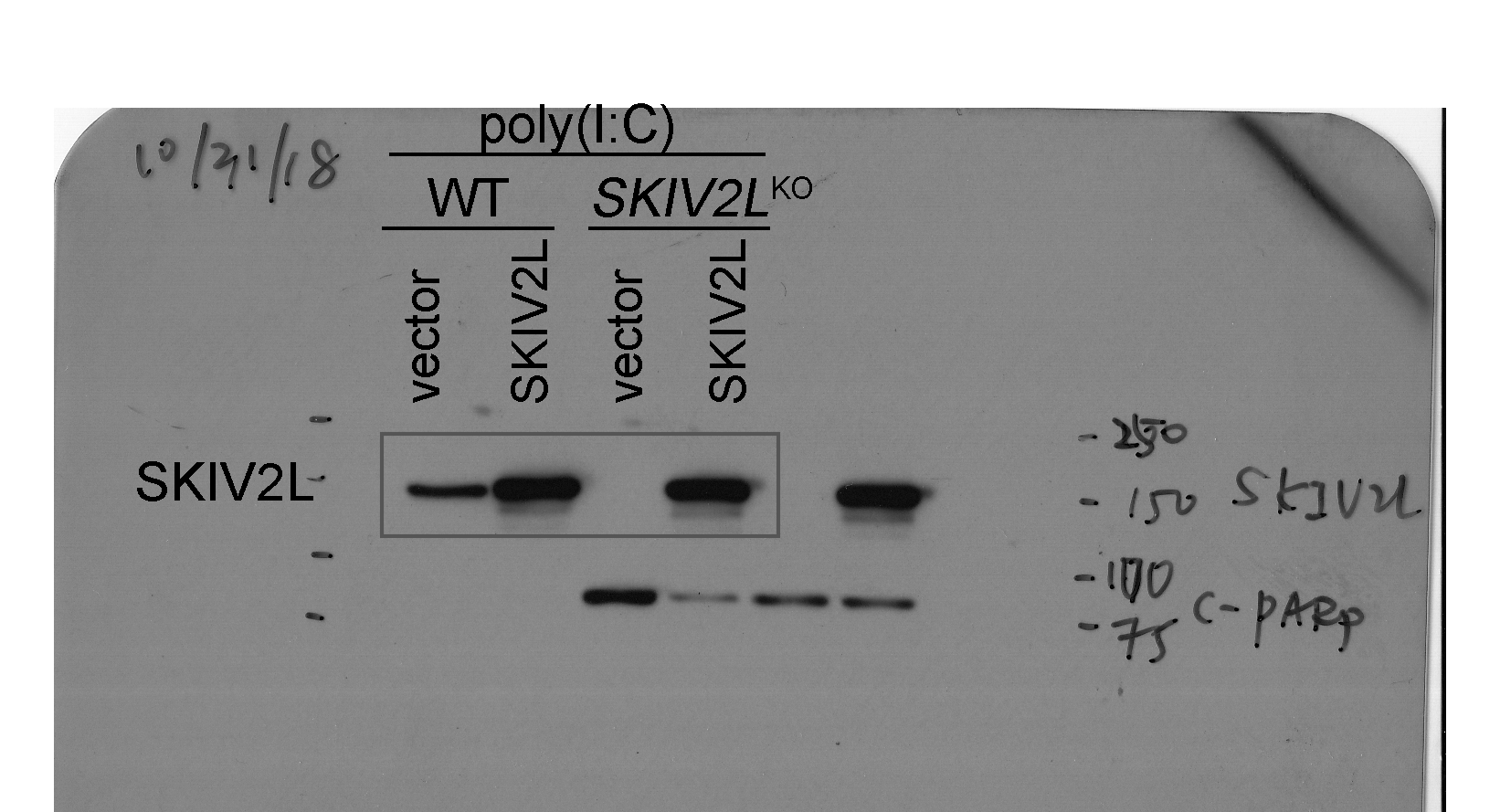

Supplement: Supplementary file 3 — Source data Fig. 2 [file 44318_2024_187_MOESM3_ESM.zip › Figure2/2D/SKIV2L.tif]

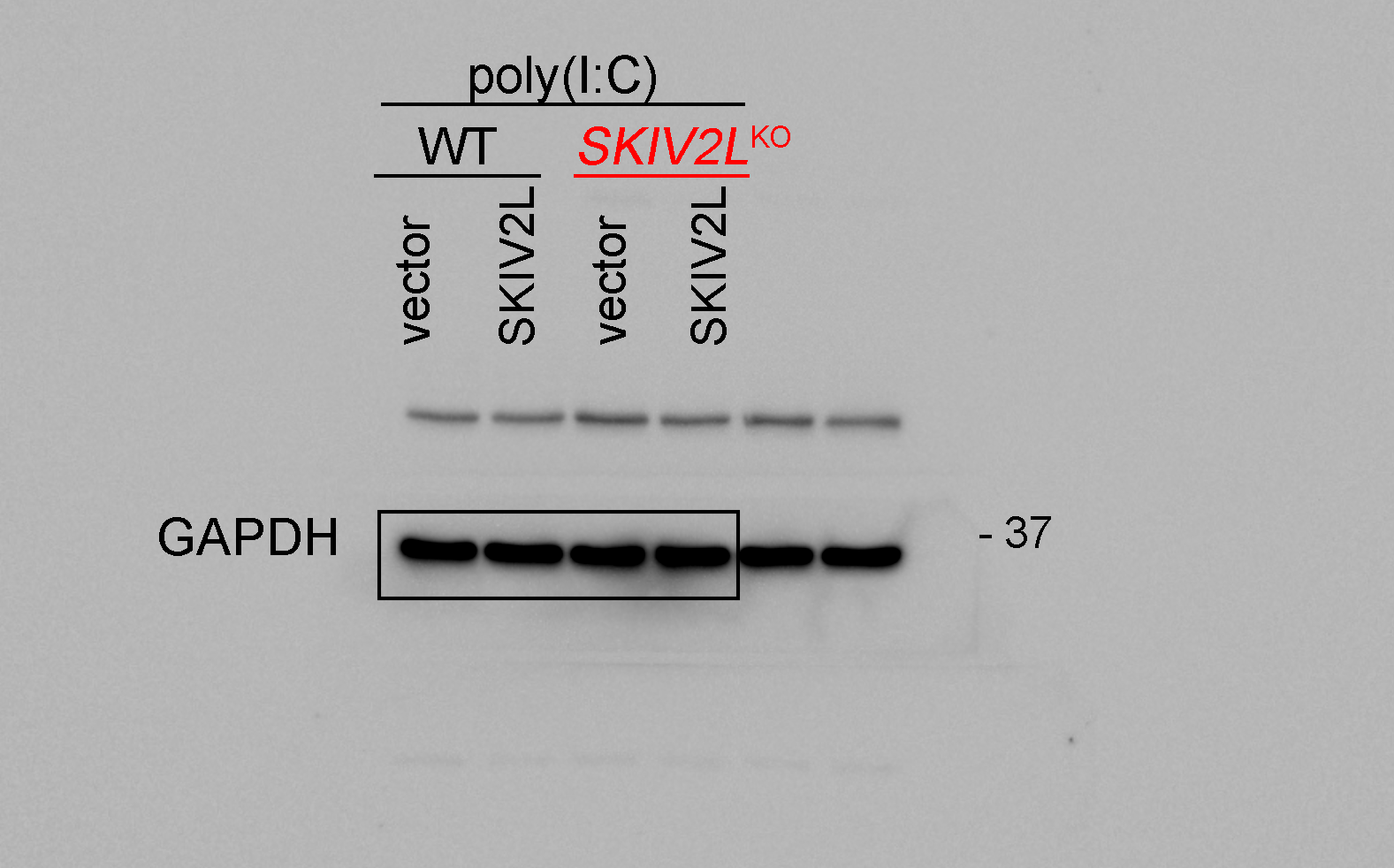

Supplement: Supplementary file 3 — Source data Fig. 2 [file 44318_2024_187_MOESM3_ESM.zip › Figure2/2D/GAPDH.tif]

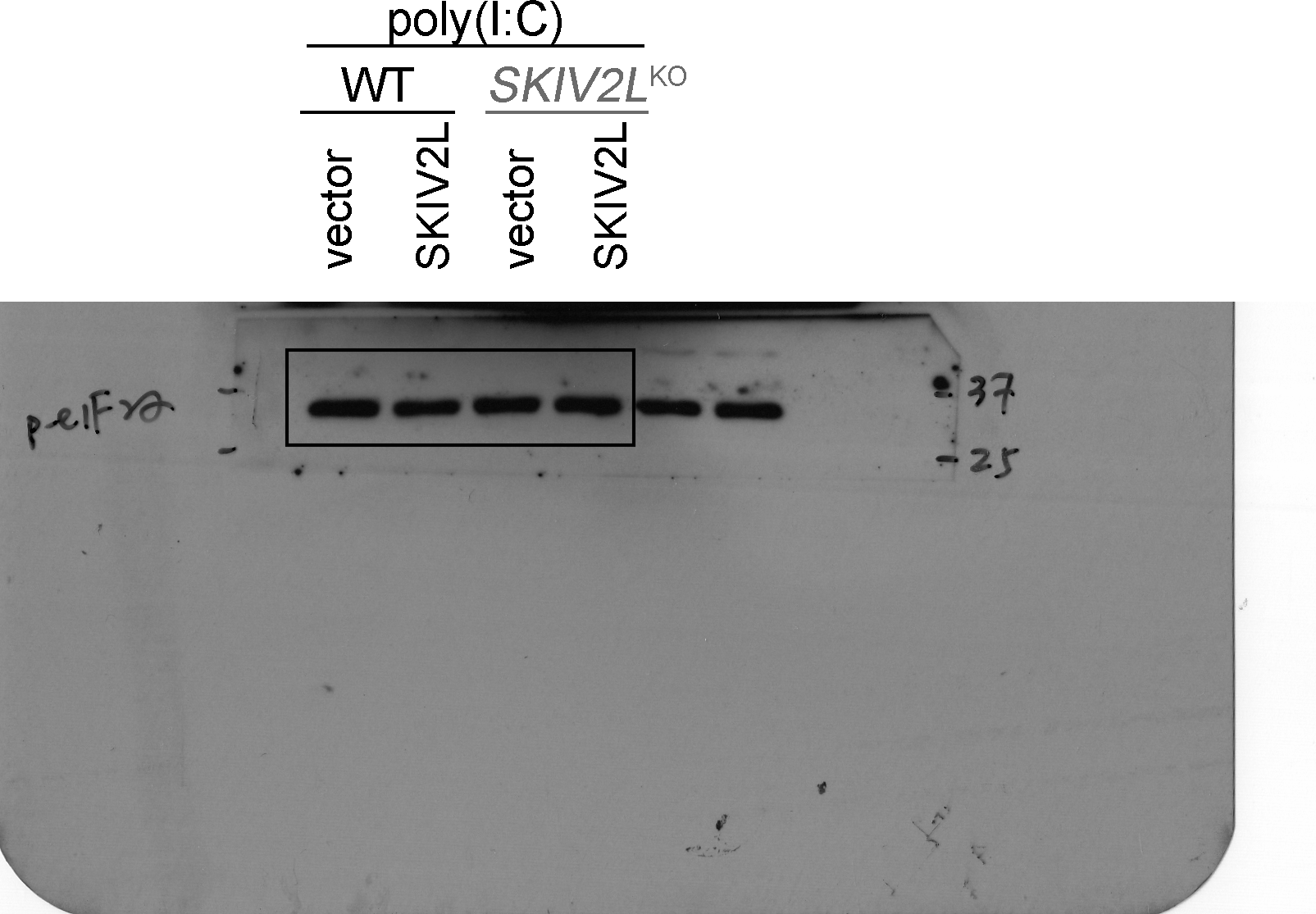

Supplement: Supplementary file 3 — Source data Fig. 2 [file 44318_2024_187_MOESM3_ESM.zip › Figure2/2D/p-eIF2a copy.tif]

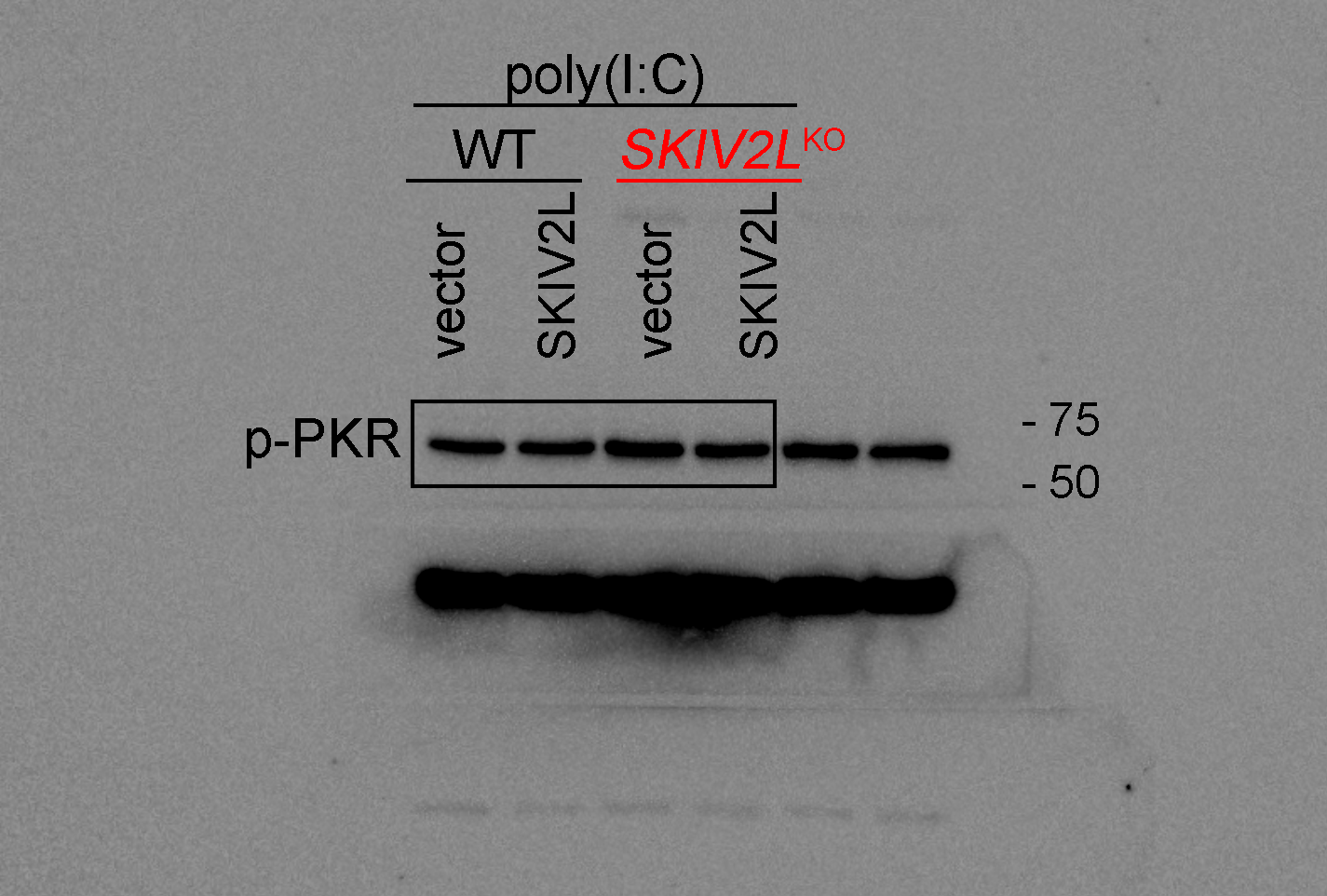

Supplement: Supplementary file 3 — Source data Fig. 2 [file 44318_2024_187_MOESM3_ESM.zip › Figure2/2D/p-PKR.tif]

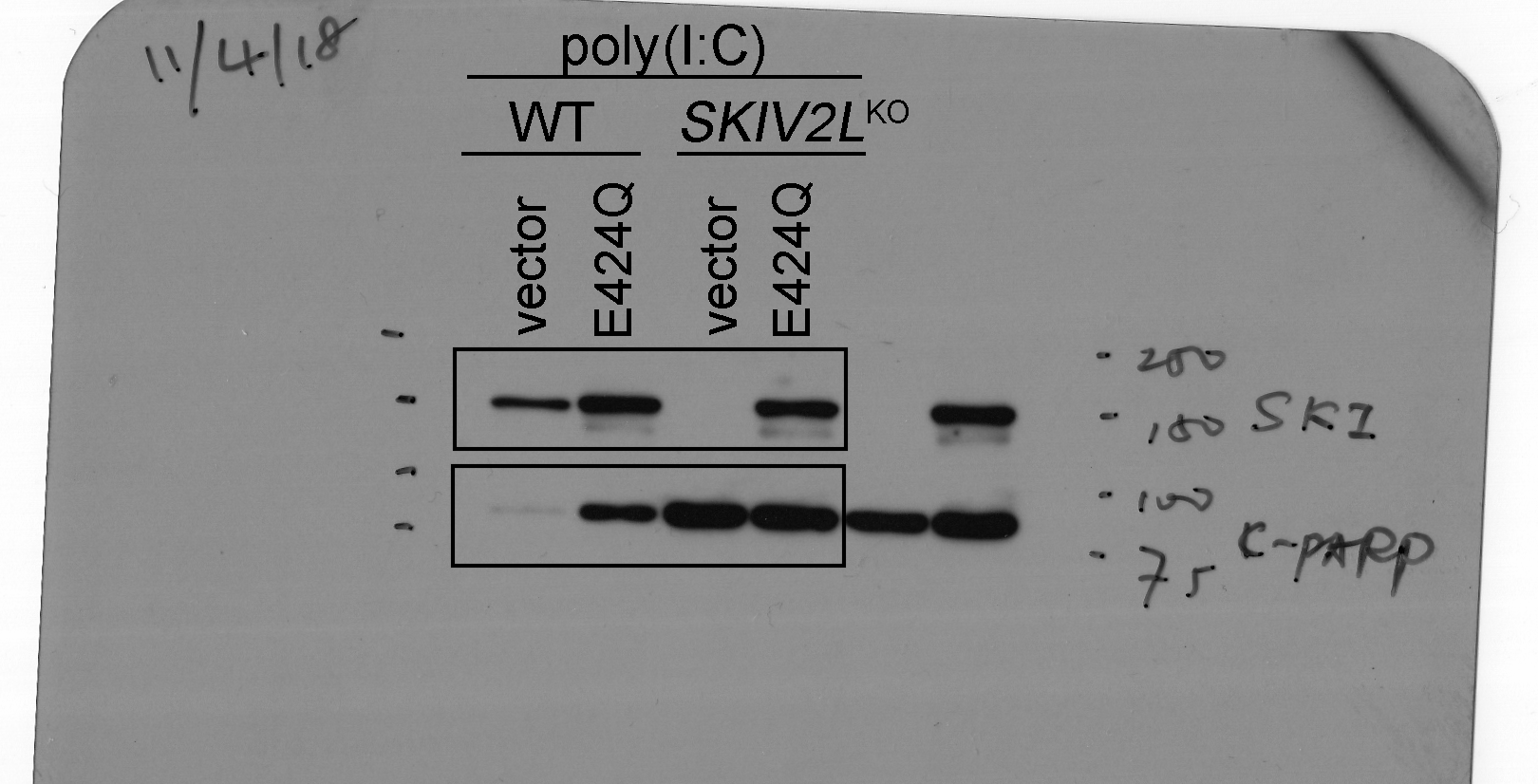

Supplement: Supplementary file 3 — Source data Fig. 2 [file 44318_2024_187_MOESM3_ESM.zip › Figure2/2E/SKIV2L c-PARP.tif]

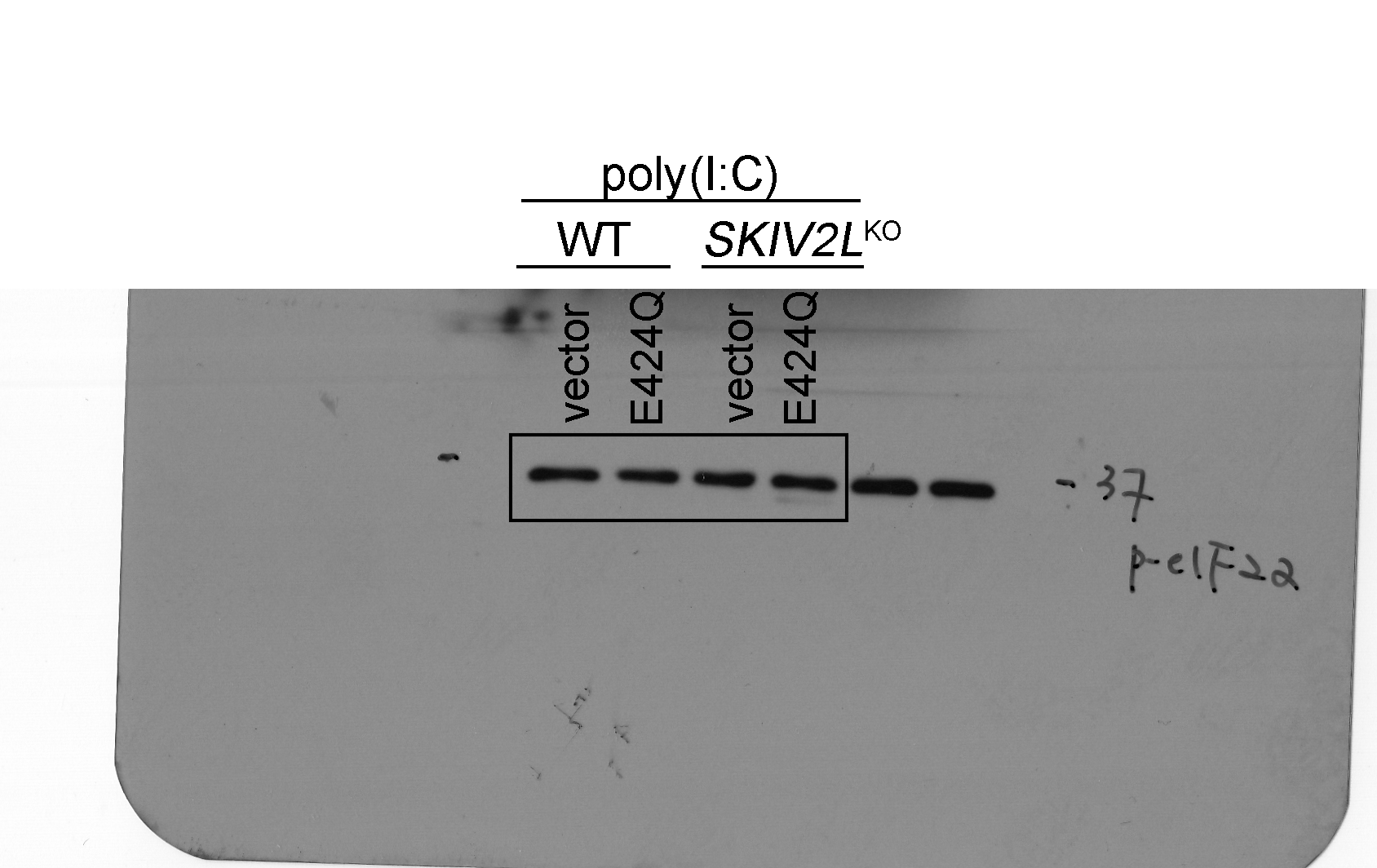

Supplement: Supplementary file 3 — Source data Fig. 2 [file 44318_2024_187_MOESM3_ESM.zip › Figure2/2E/p-eIF2a.tif]

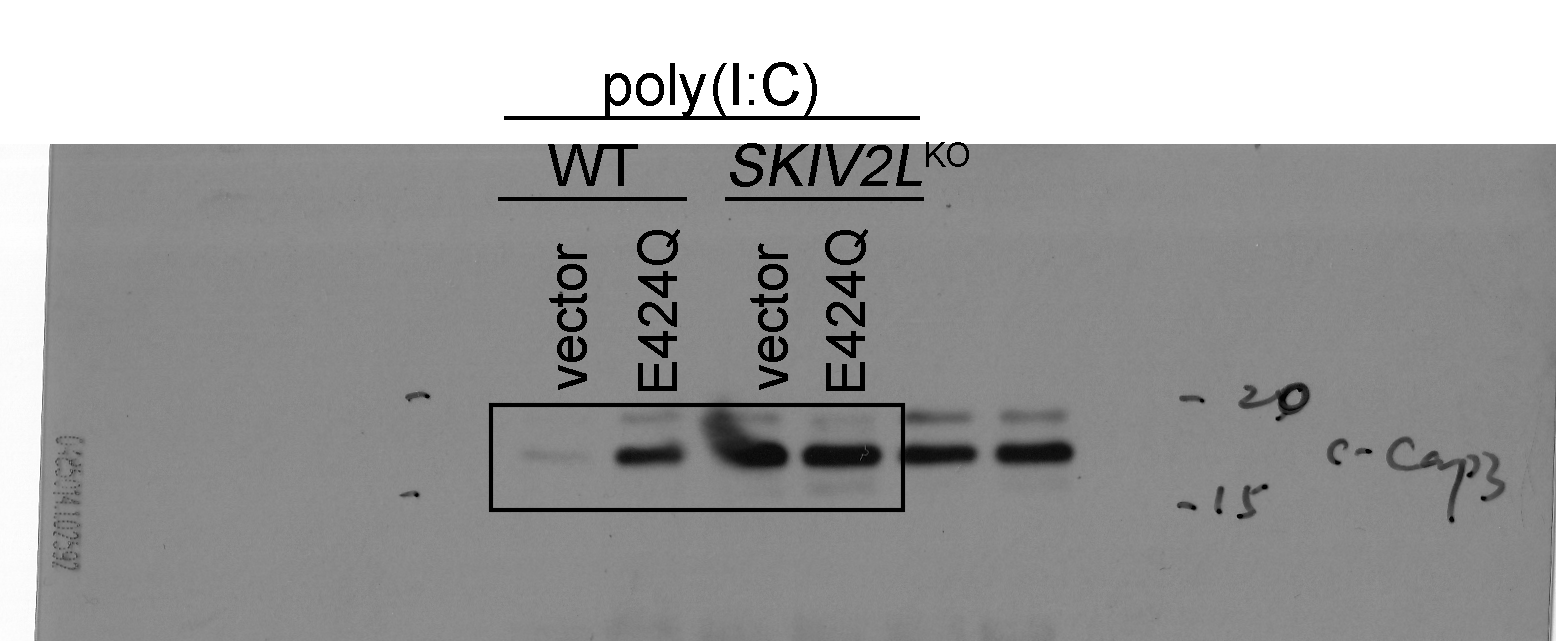

Supplement: Supplementary file 3 — Source data Fig. 2 [file 44318_2024_187_MOESM3_ESM.zip › Figure2/2E/c-Casp3.tif]

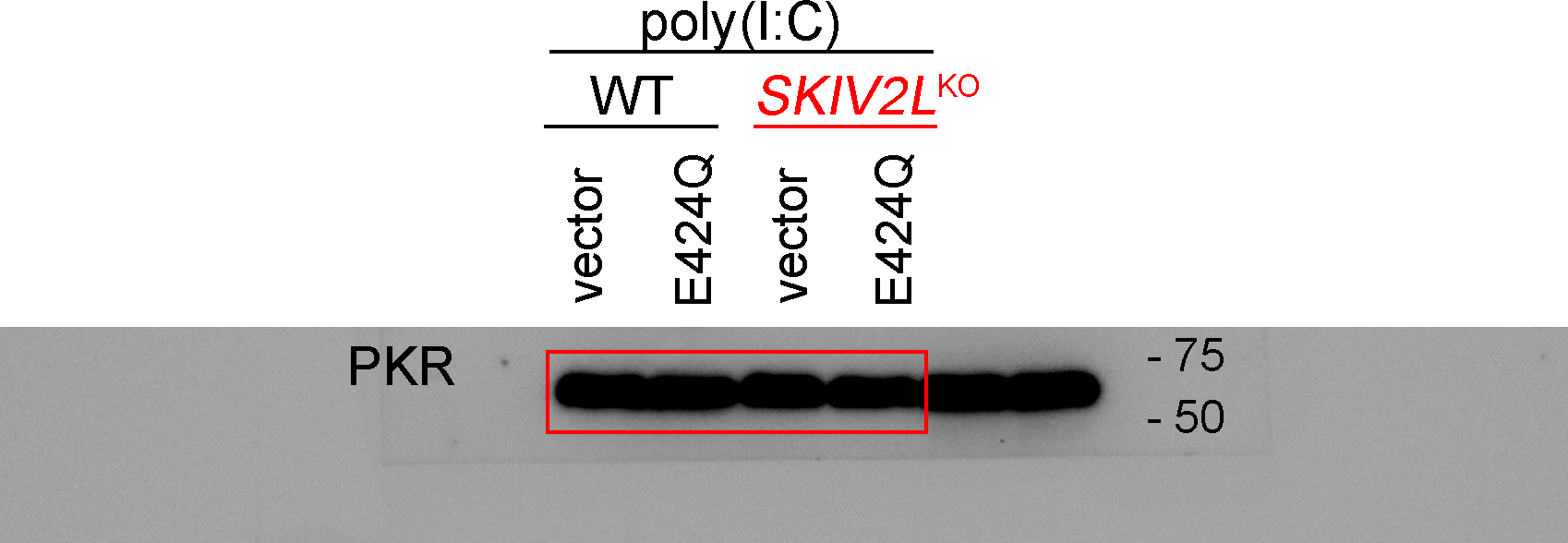

Supplement: Supplementary file 3 — Source data Fig. 2 [file 44318_2024_187_MOESM3_ESM.zip › Figure2/2E/PKR.tif]

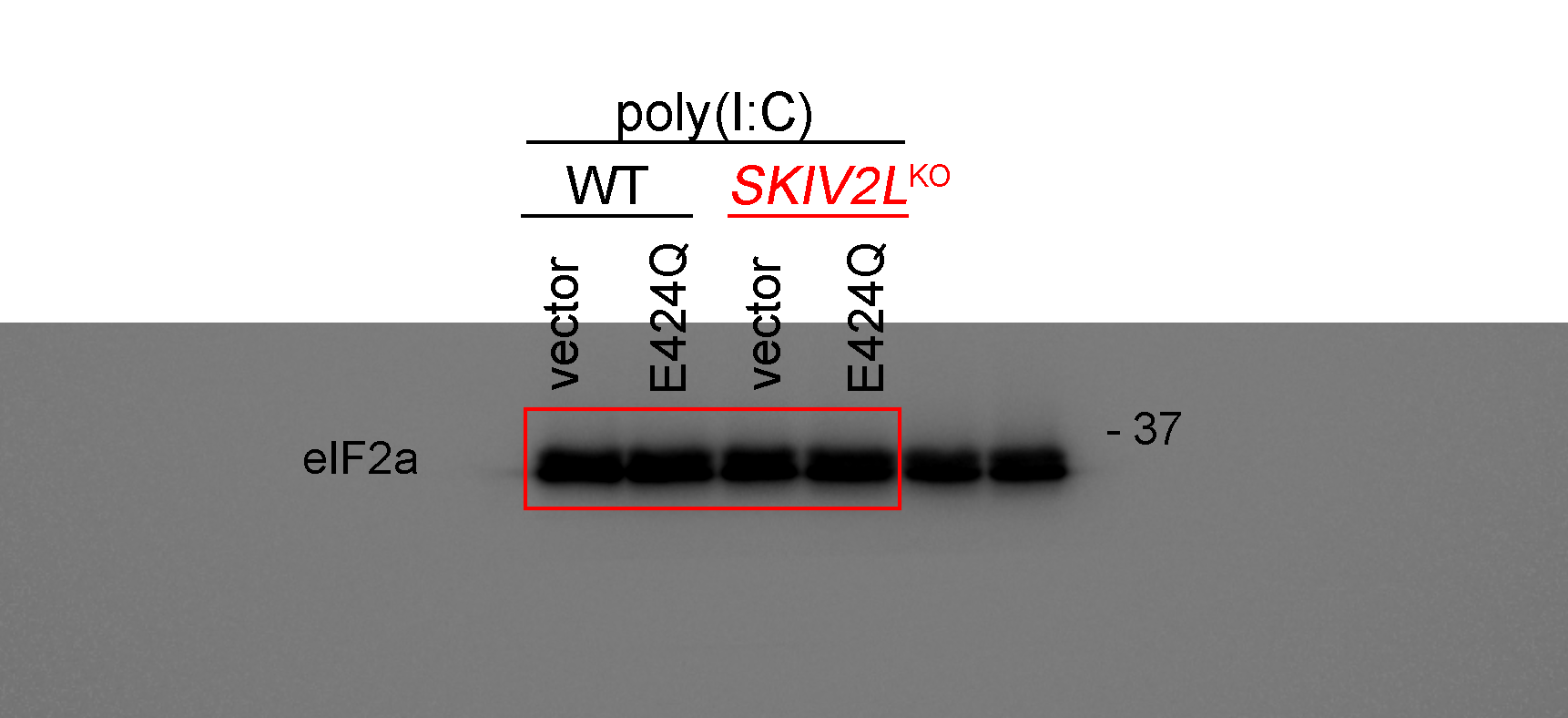

Supplement: Supplementary file 3 — Source data Fig. 2 [file 44318_2024_187_MOESM3_ESM.zip › Figure2/2E/eIF2a.tif]

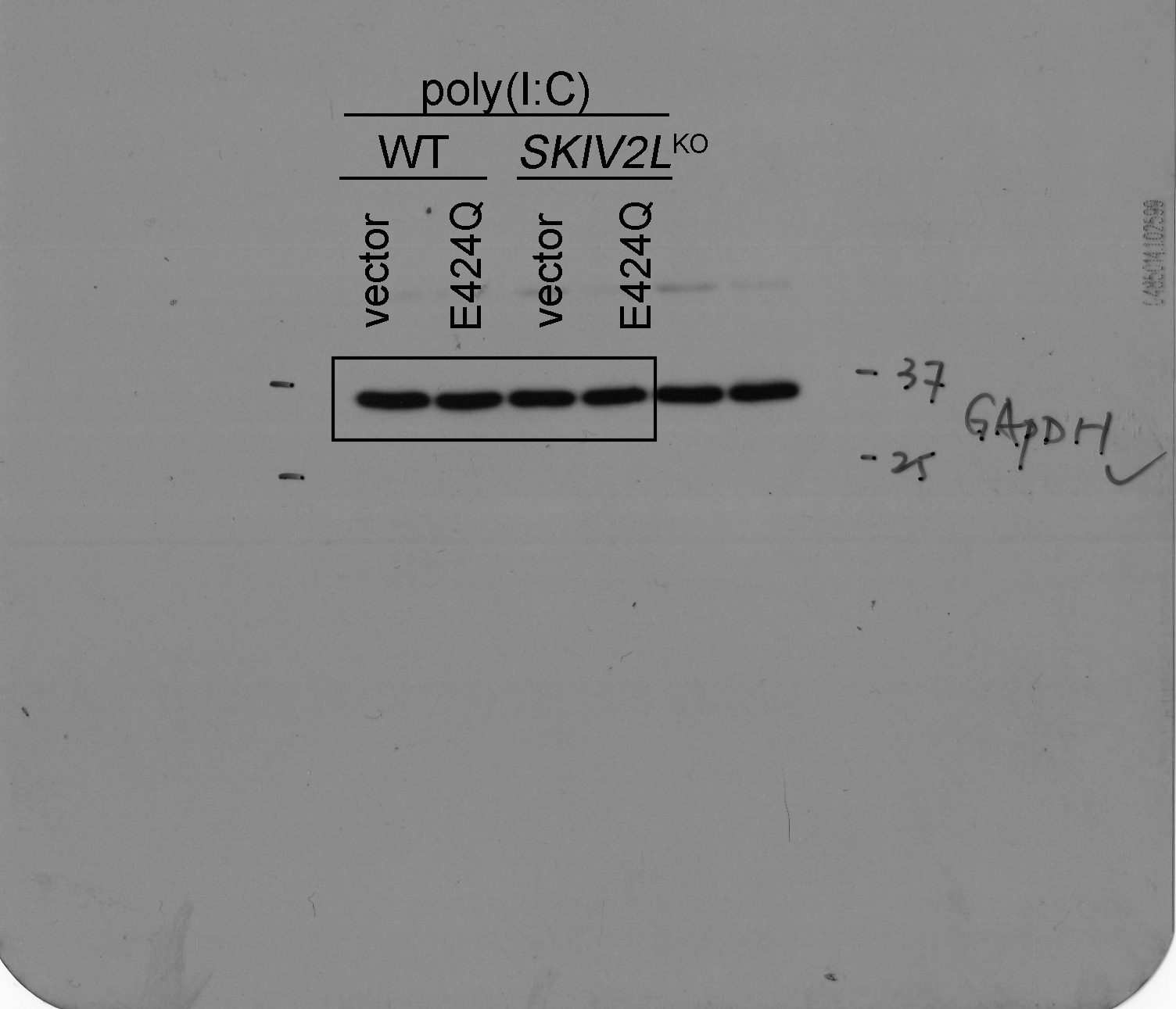

Supplement: Supplementary file 3 — Source data Fig. 2 [file 44318_2024_187_MOESM3_ESM.zip › Figure2/2E/GAPDH.tif]

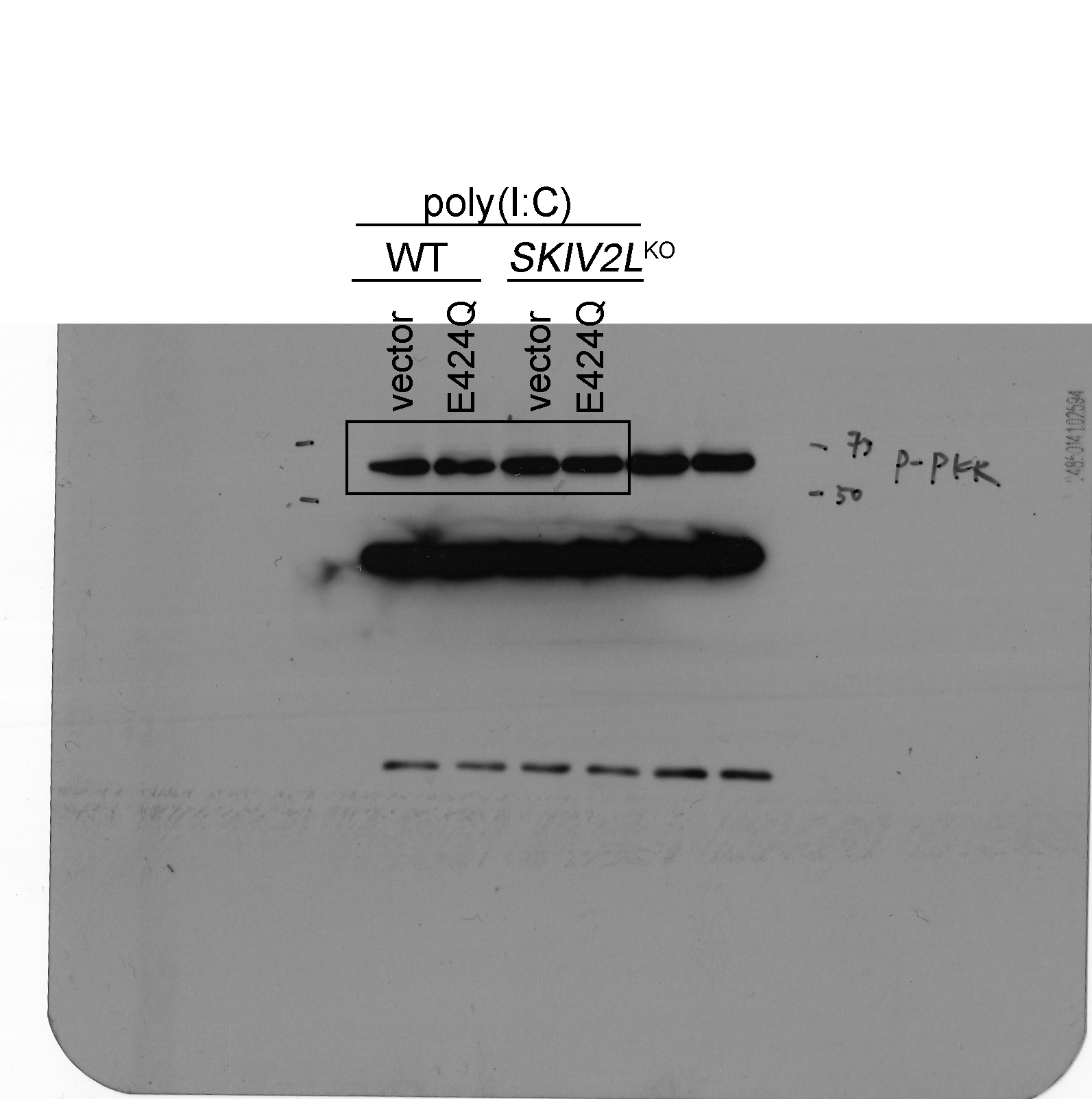

Supplement: Supplementary file 3 — Source data Fig. 2 [file 44318_2024_187_MOESM3_ESM.zip › Figure2/2E/p-PKR.tif]

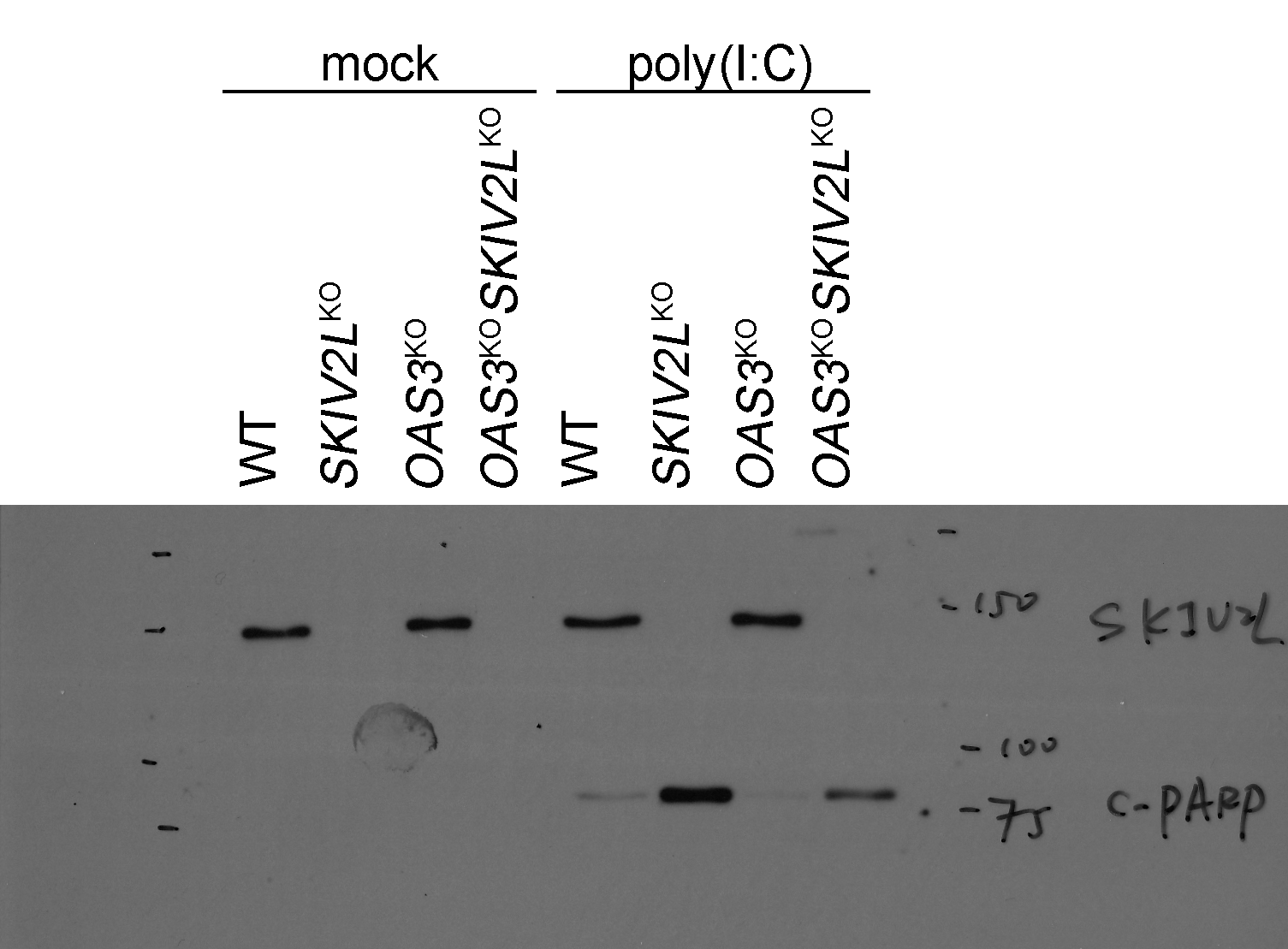

Supplement: Supplementary file 4 — Source data Fig. 3 [file 44318_2024_187_MOESM4_ESM.zip › Figure3/3B/SKIV2L c-PARP.tif]

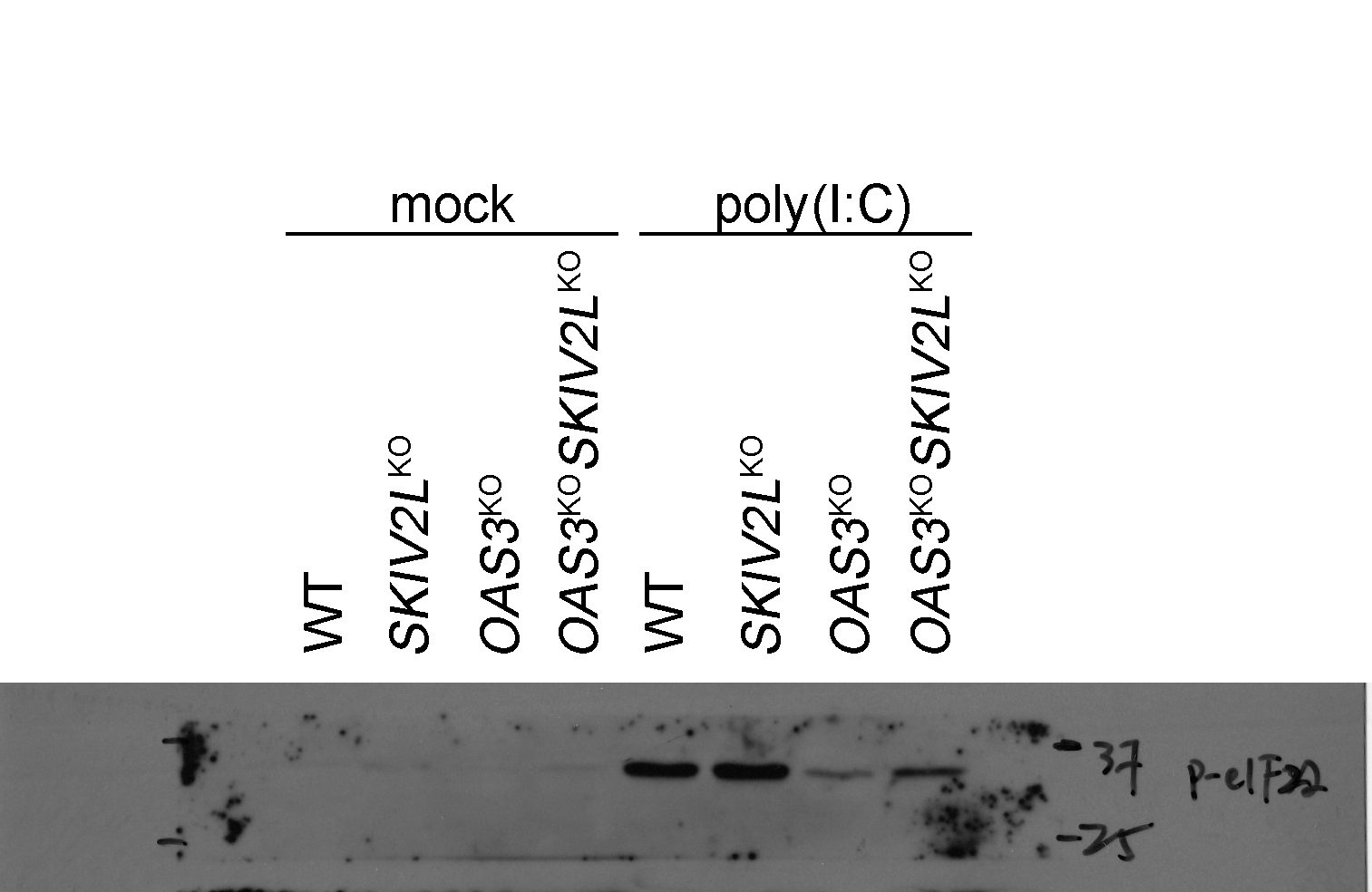

Supplement: Supplementary file 4 — Source data Fig. 3 [file 44318_2024_187_MOESM4_ESM.zip › Figure3/3B/p-eIF2a.tif]

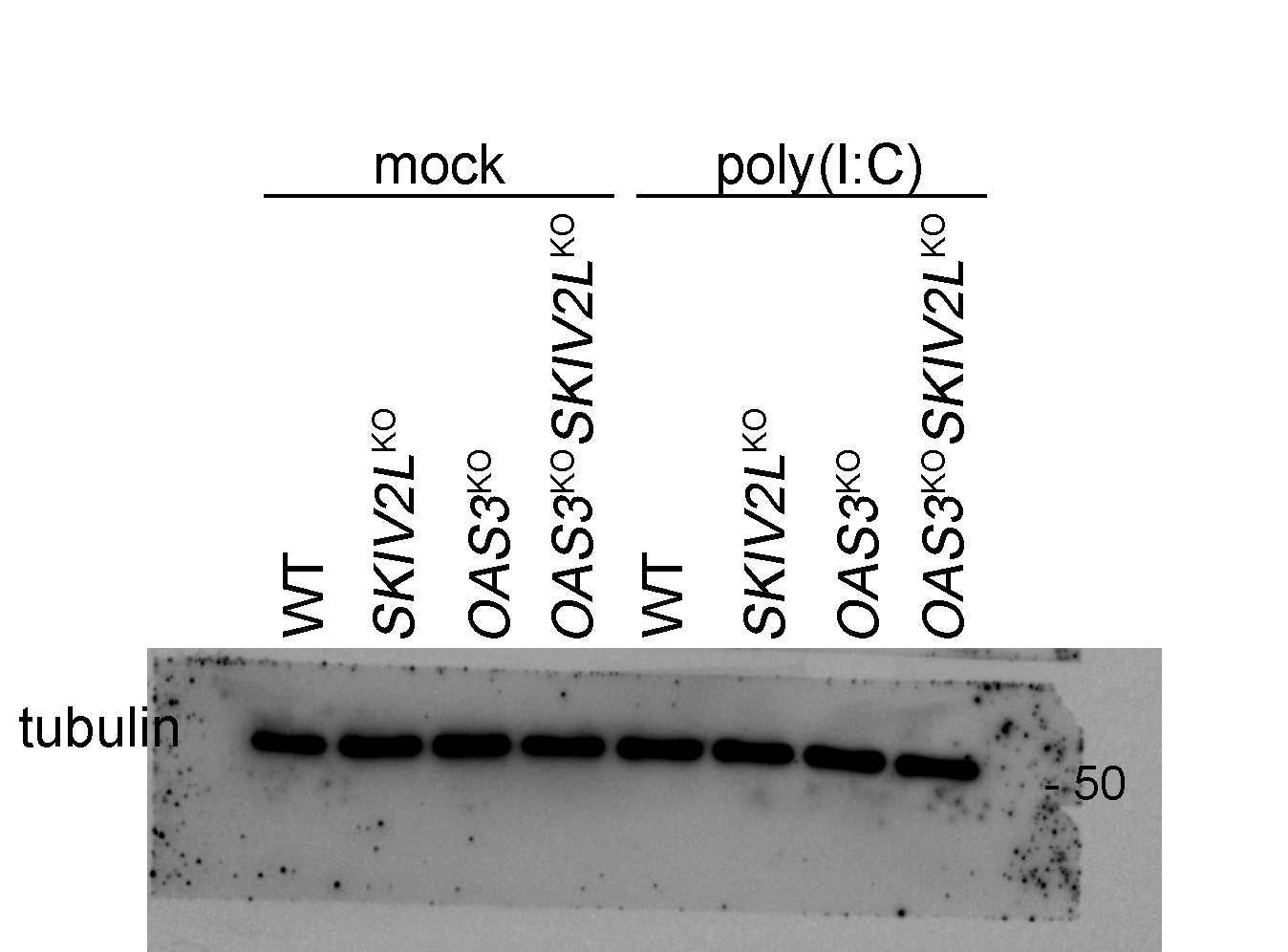

Supplement: Supplementary file 4 — Source data Fig. 3 [file 44318_2024_187_MOESM4_ESM.zip › Figure3/3B/Tubulin.tif]

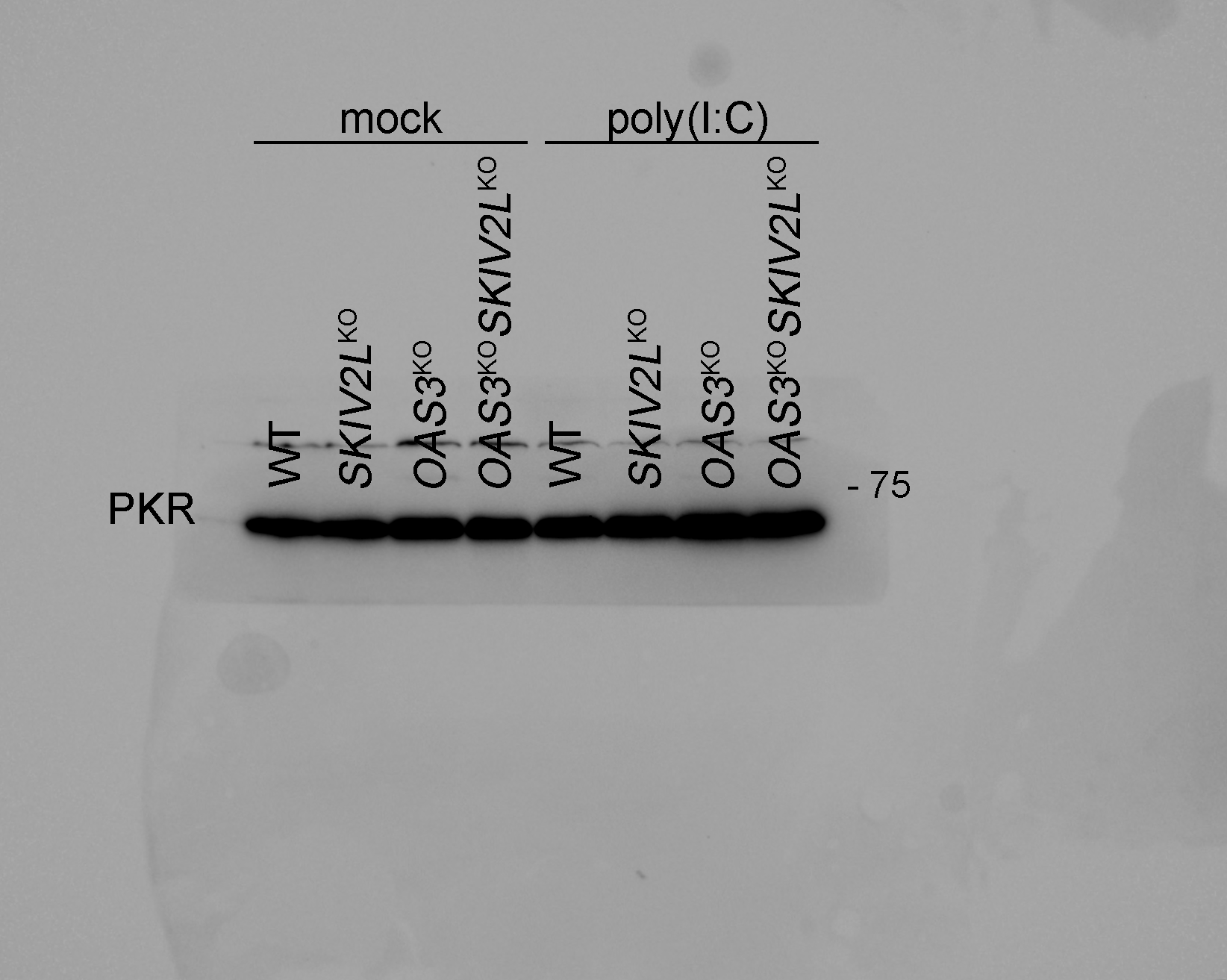

Supplement: Supplementary file 4 — Source data Fig. 3 [file 44318_2024_187_MOESM4_ESM.zip › Figure3/3B/PKR.tif]

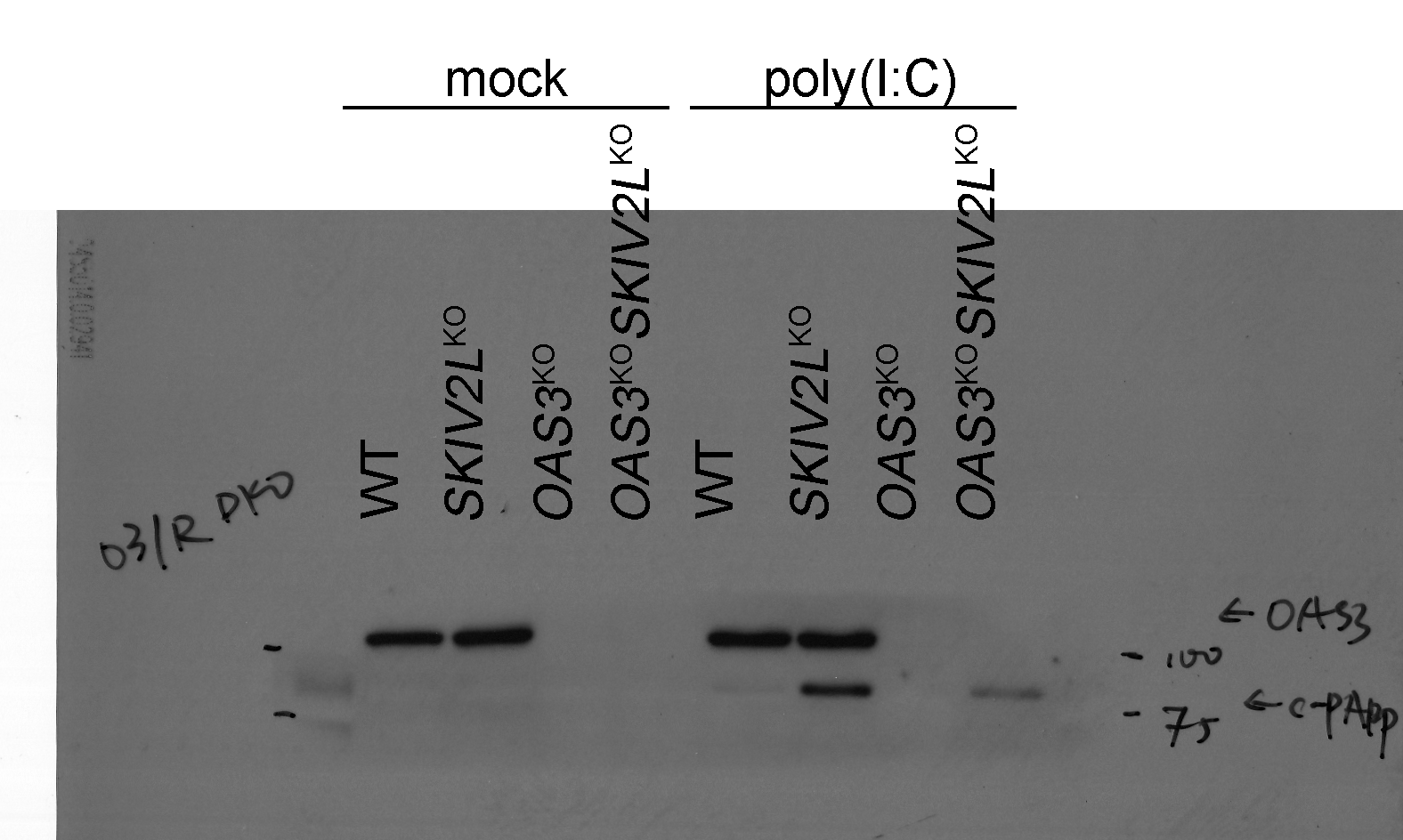

Supplement: Supplementary file 4 — Source data Fig. 3 [file 44318_2024_187_MOESM4_ESM.zip › Figure3/3B/OAS3.tif]

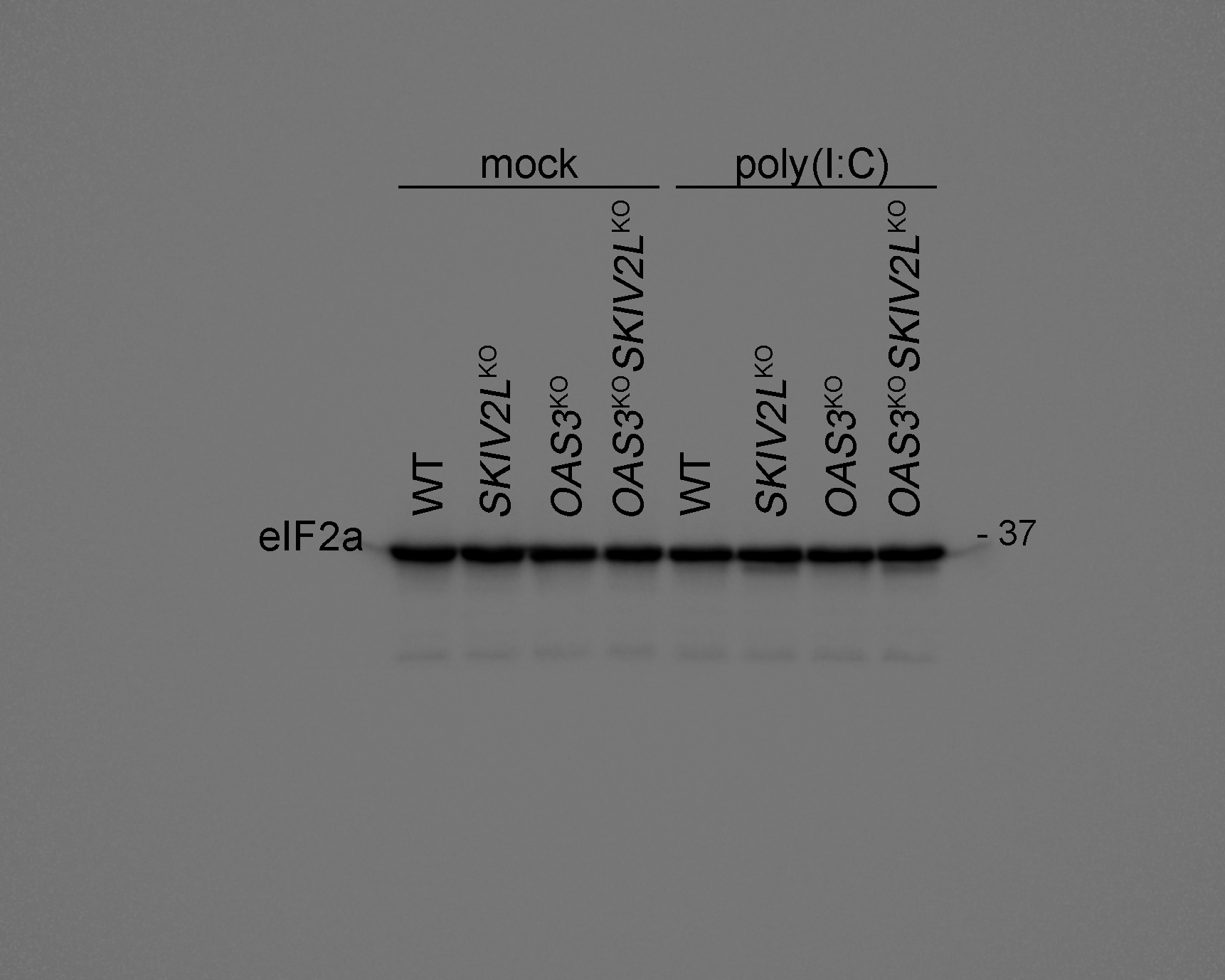

Supplement: Supplementary file 4 — Source data Fig. 3 [file 44318_2024_187_MOESM4_ESM.zip › Figure3/3B/eIF2a.tif]

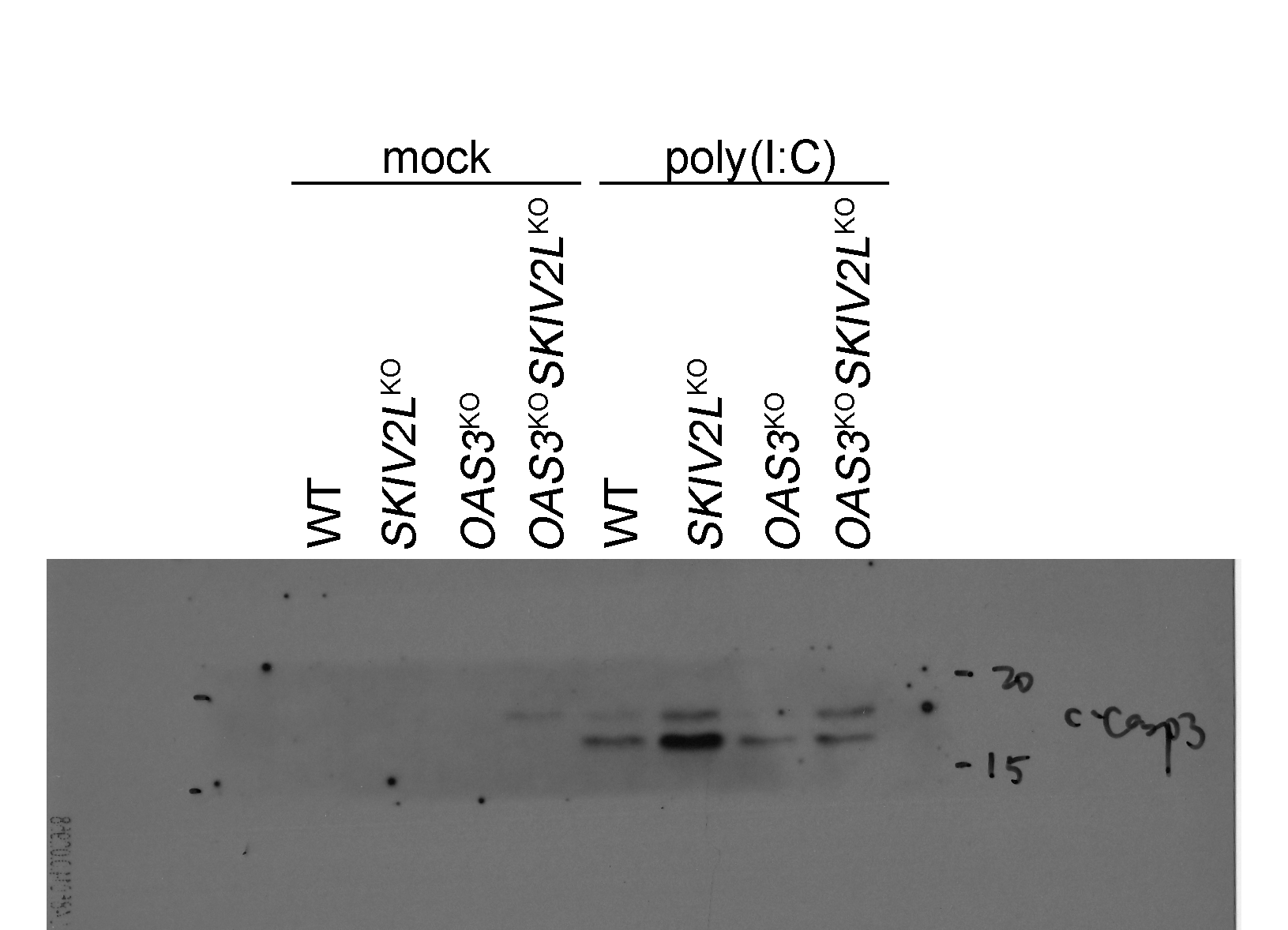

Supplement: Supplementary file 4 — Source data Fig. 3 [file 44318_2024_187_MOESM4_ESM.zip › Figure3/3B/cCasp3.tif]

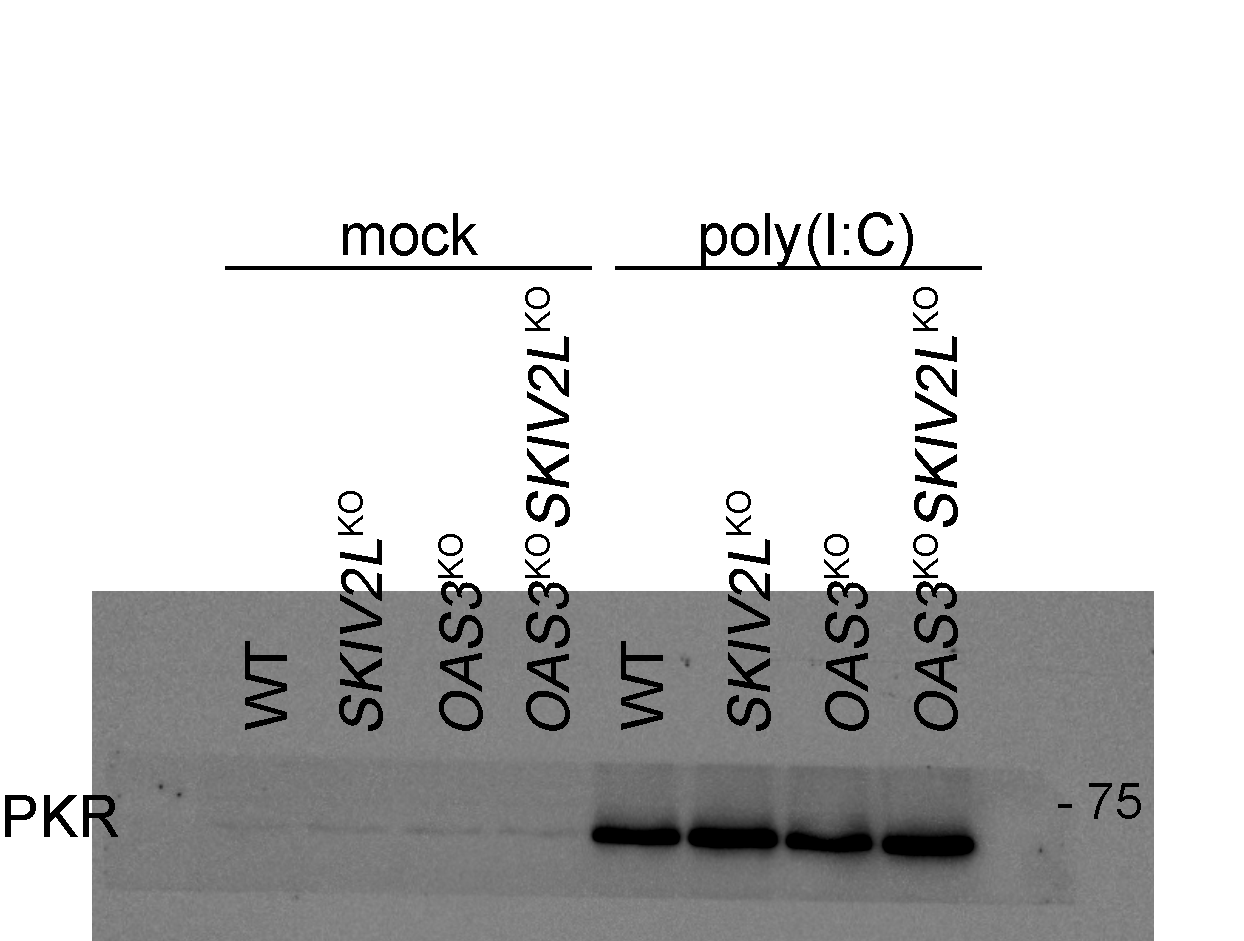

Supplement: Supplementary file 4 — Source data Fig. 3 [file 44318_2024_187_MOESM4_ESM.zip › Figure3/3B/p-PKR.tif]

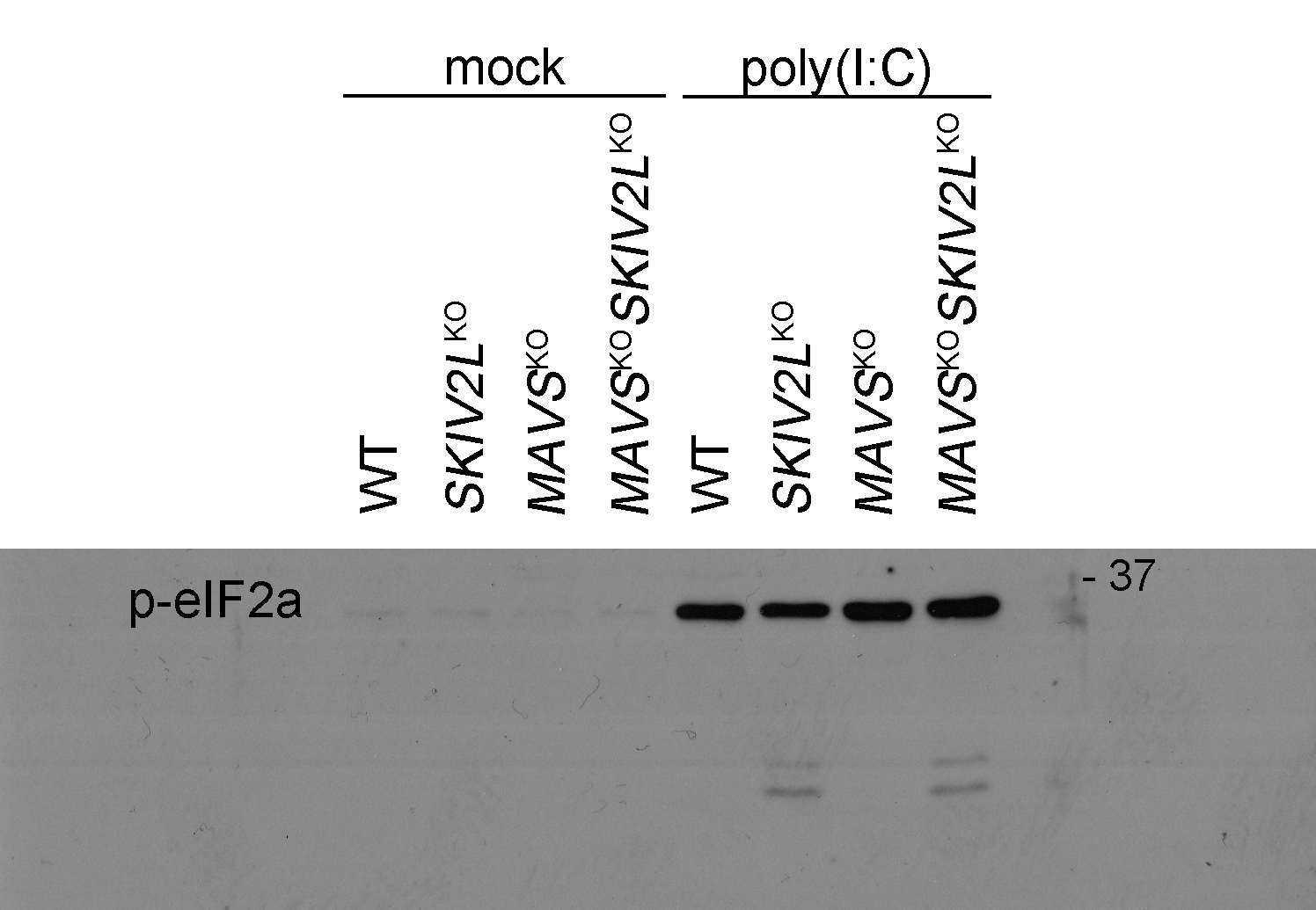

Supplement: Supplementary file 4 — Source data Fig. 3 [file 44318_2024_187_MOESM4_ESM.zip › Figure3/3C/p-eIF2a.tif]

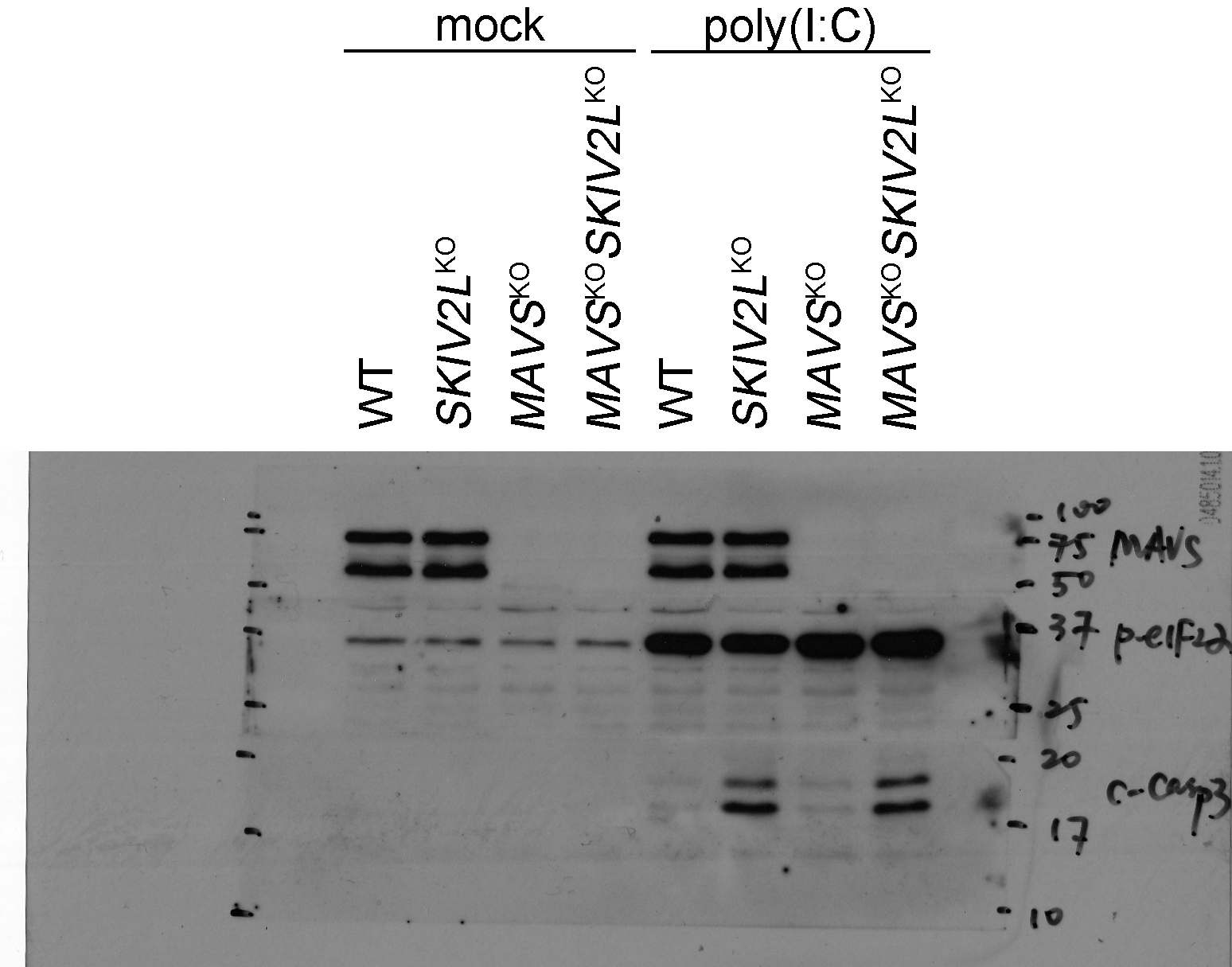

Supplement: Supplementary file 4 — Source data Fig. 3 [file 44318_2024_187_MOESM4_ESM.zip › Figure3/3C/MAVS c-Casp3.tif]

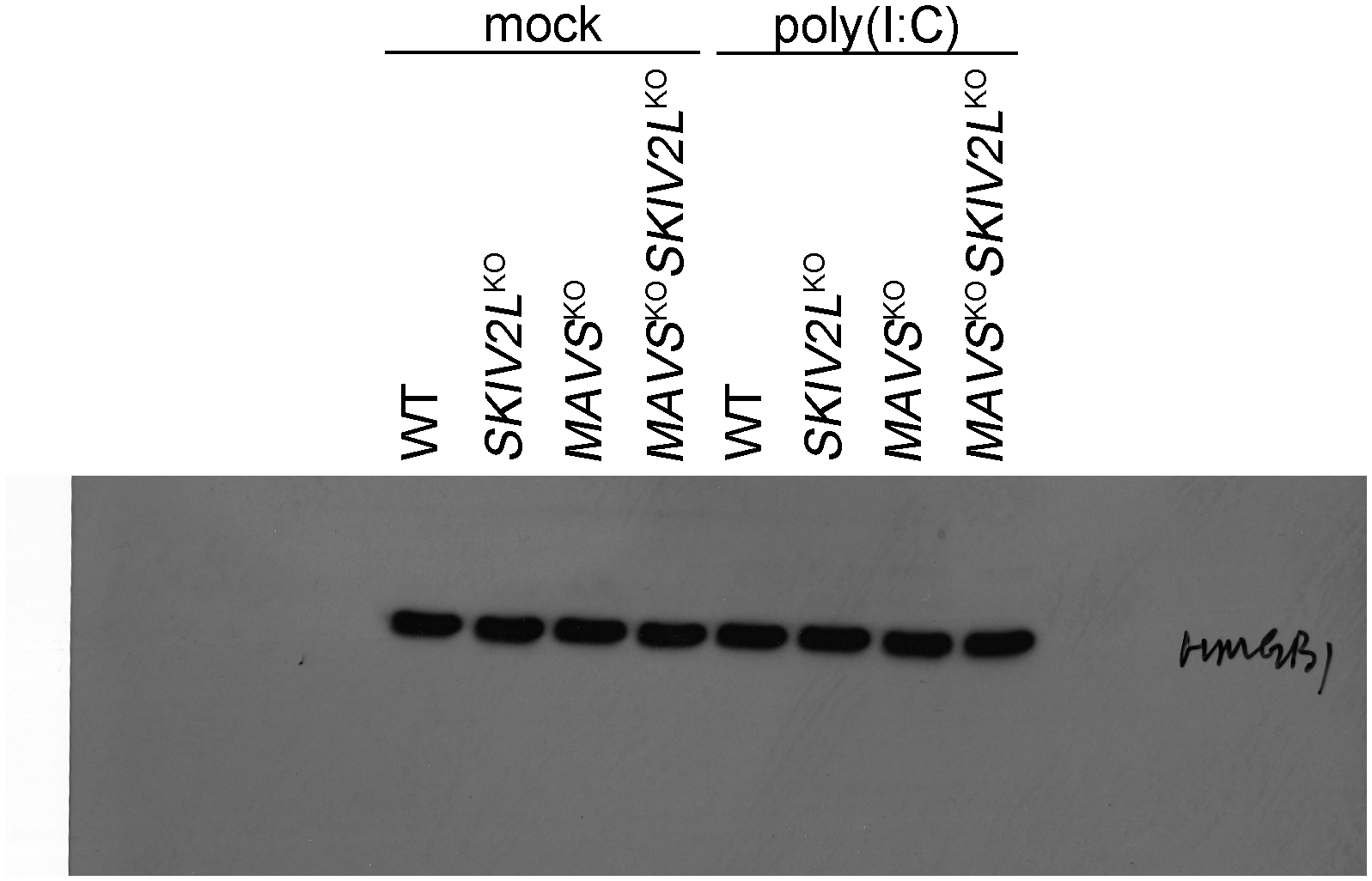

Supplement: Supplementary file 4 — Source data Fig. 3 [file 44318_2024_187_MOESM4_ESM.zip › Figure3/3C/HMGB1.tif]

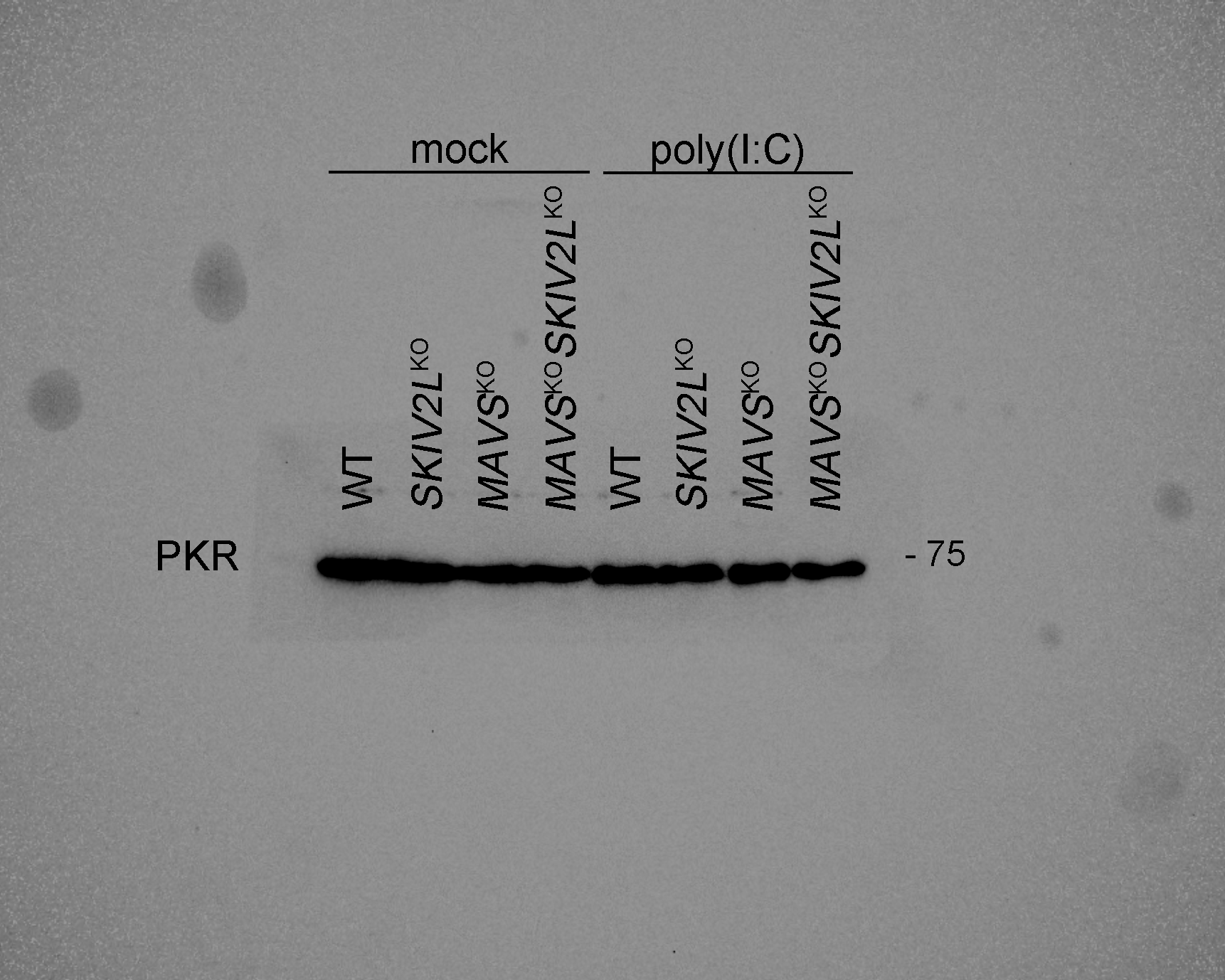

Supplement: Supplementary file 4 — Source data Fig. 3 [file 44318_2024_187_MOESM4_ESM.zip › Figure3/3C/PKR.tif]

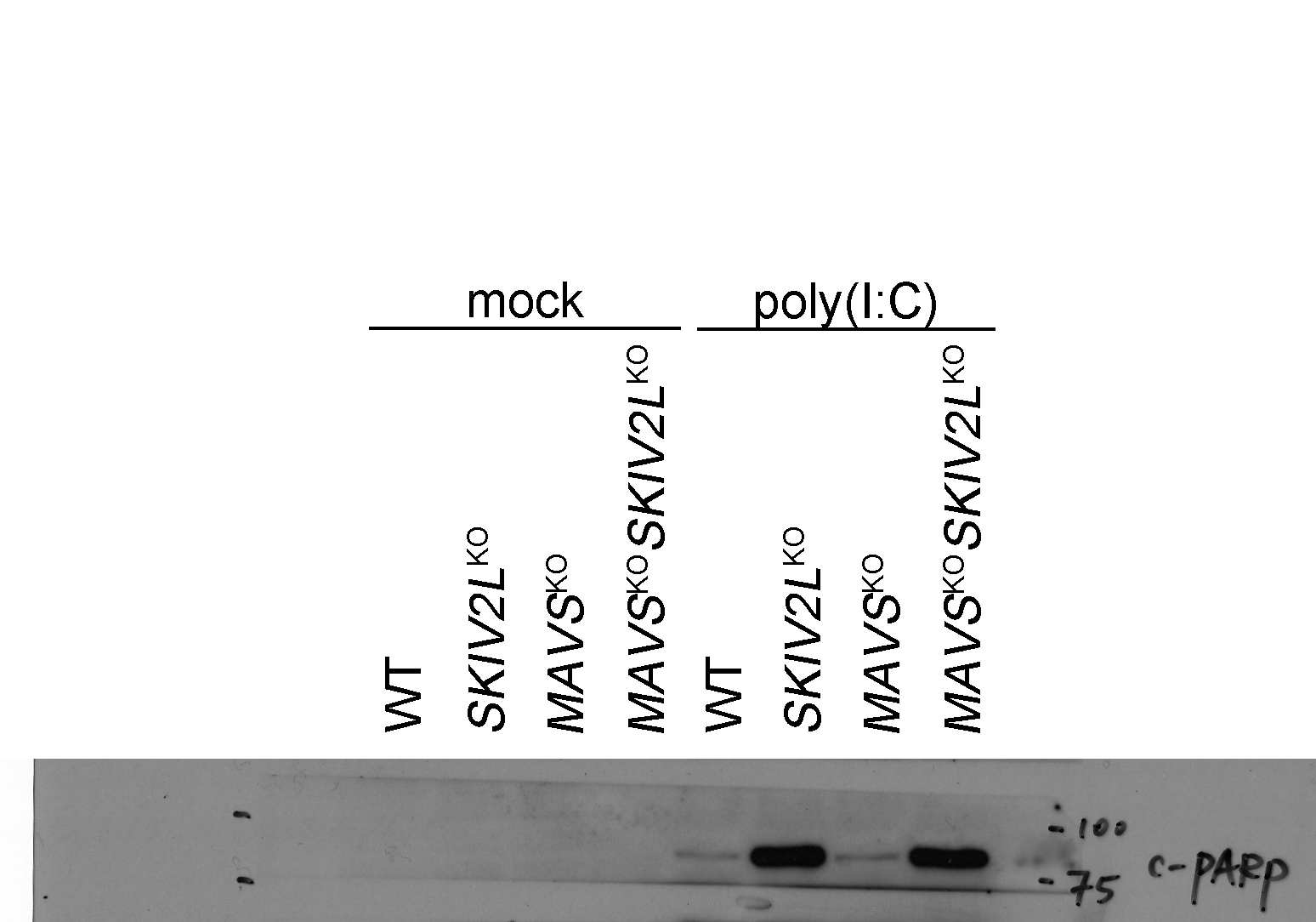

Supplement: Supplementary file 4 — Source data Fig. 3 [file 44318_2024_187_MOESM4_ESM.zip › Figure3/3C/c-PARP.tif]

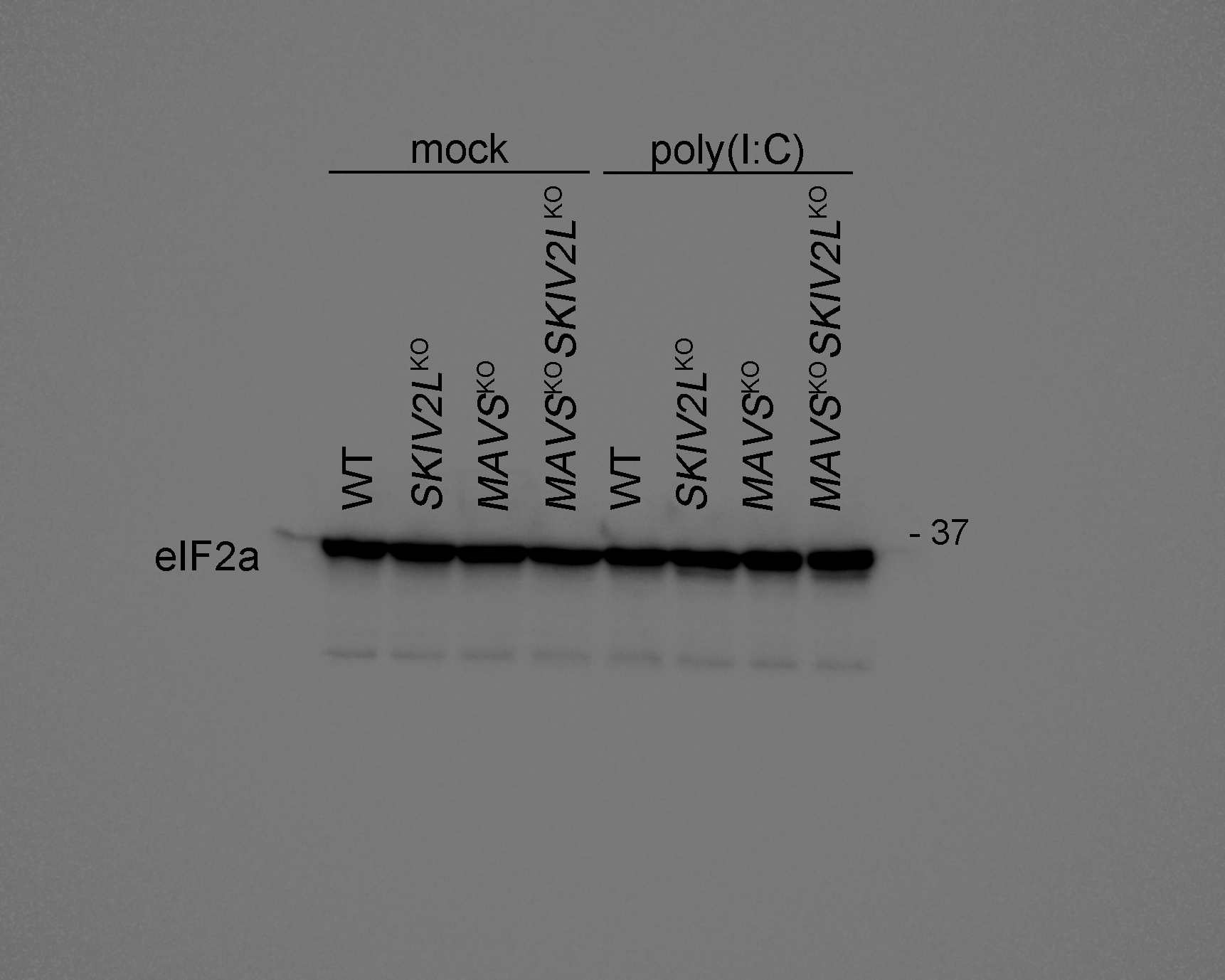

Supplement: Supplementary file 4 — Source data Fig. 3 [file 44318_2024_187_MOESM4_ESM.zip › Figure3/3C/eIF2a.tif]

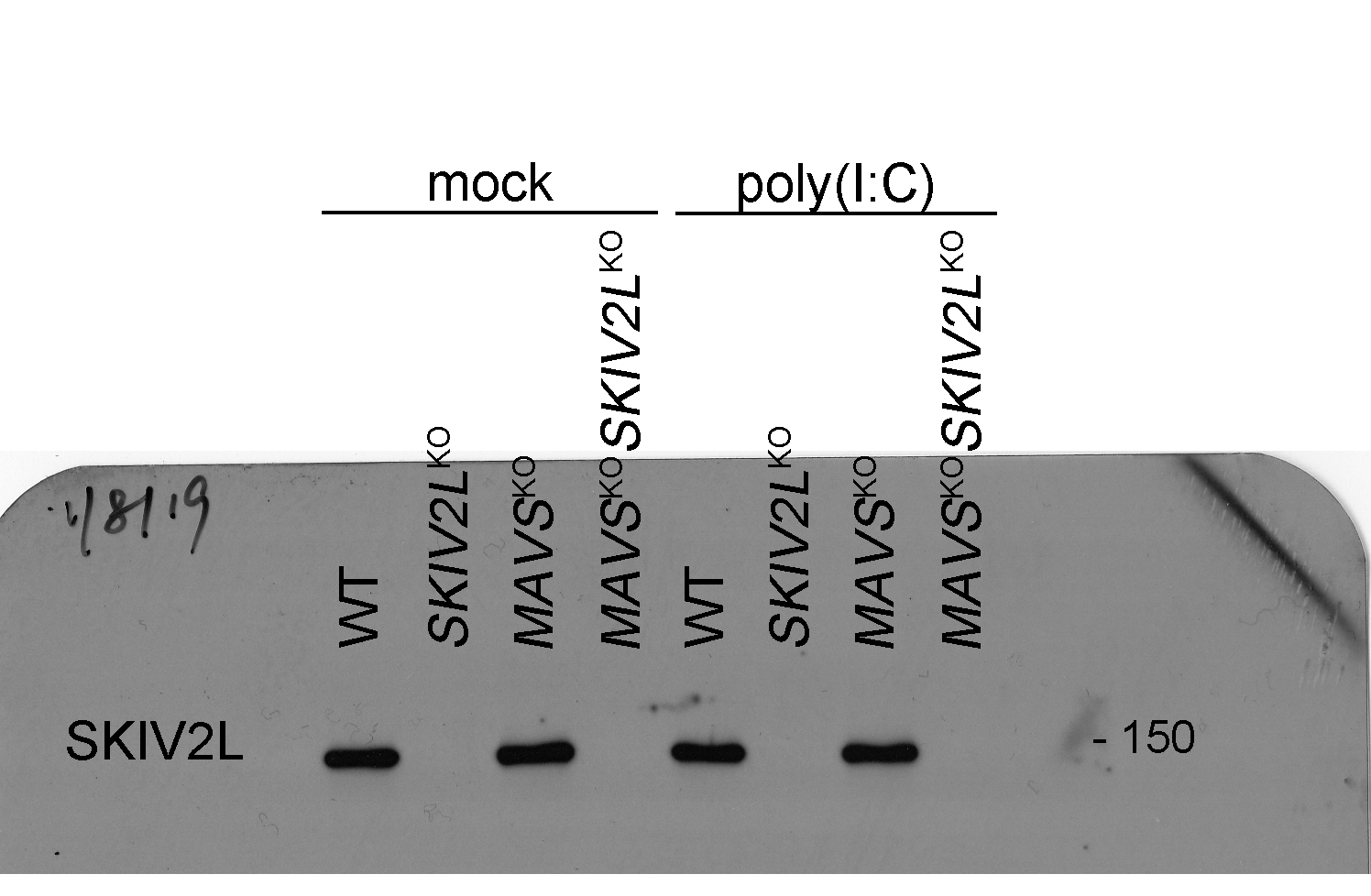

Supplement: Supplementary file 4 — Source data Fig. 3 [file 44318_2024_187_MOESM4_ESM.zip › Figure3/3C/SKIV2L.tif]

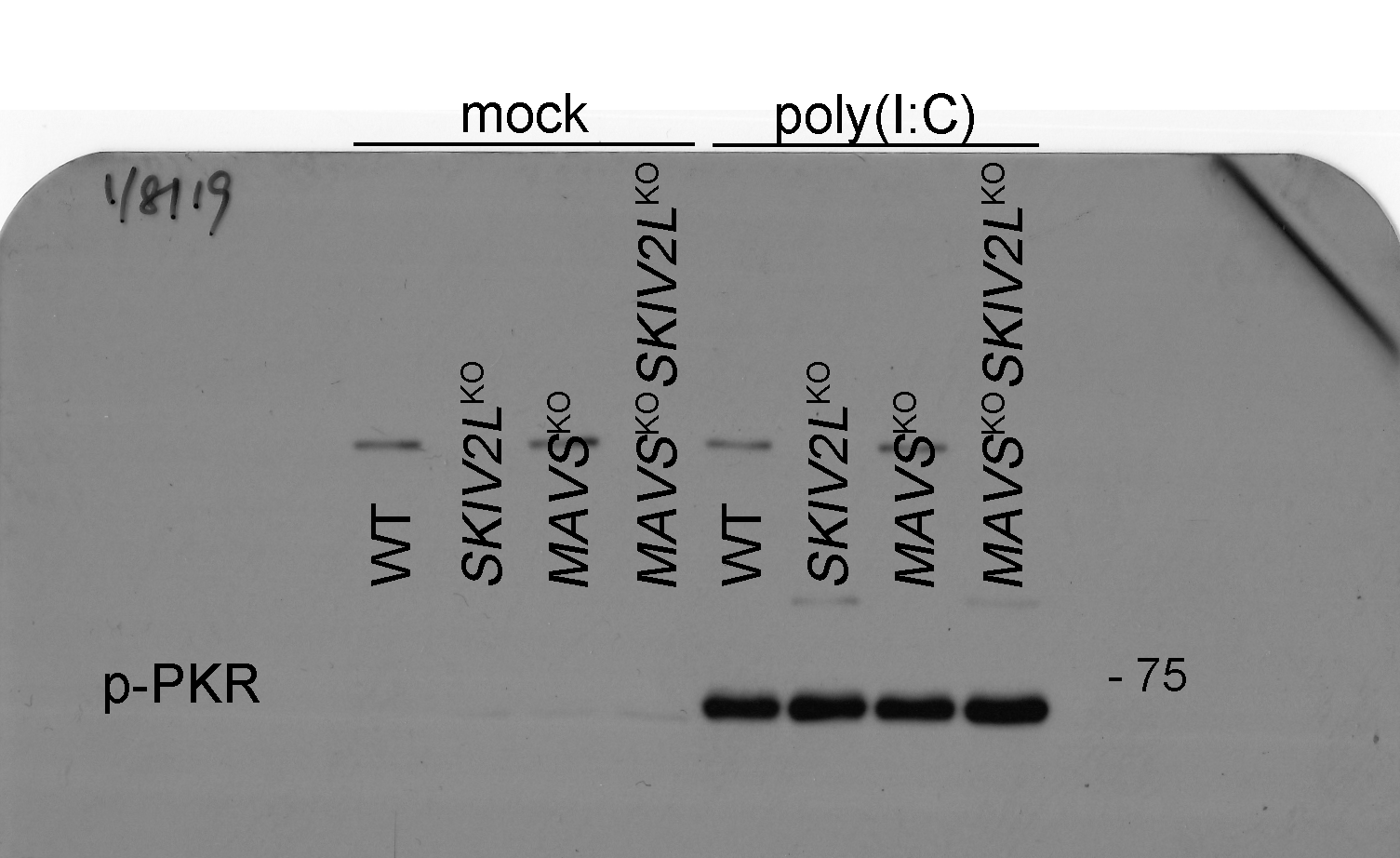

Supplement: Supplementary file 4 — Source data Fig. 3 [file 44318_2024_187_MOESM4_ESM.zip › Figure3/3C/p-PKR.tif]

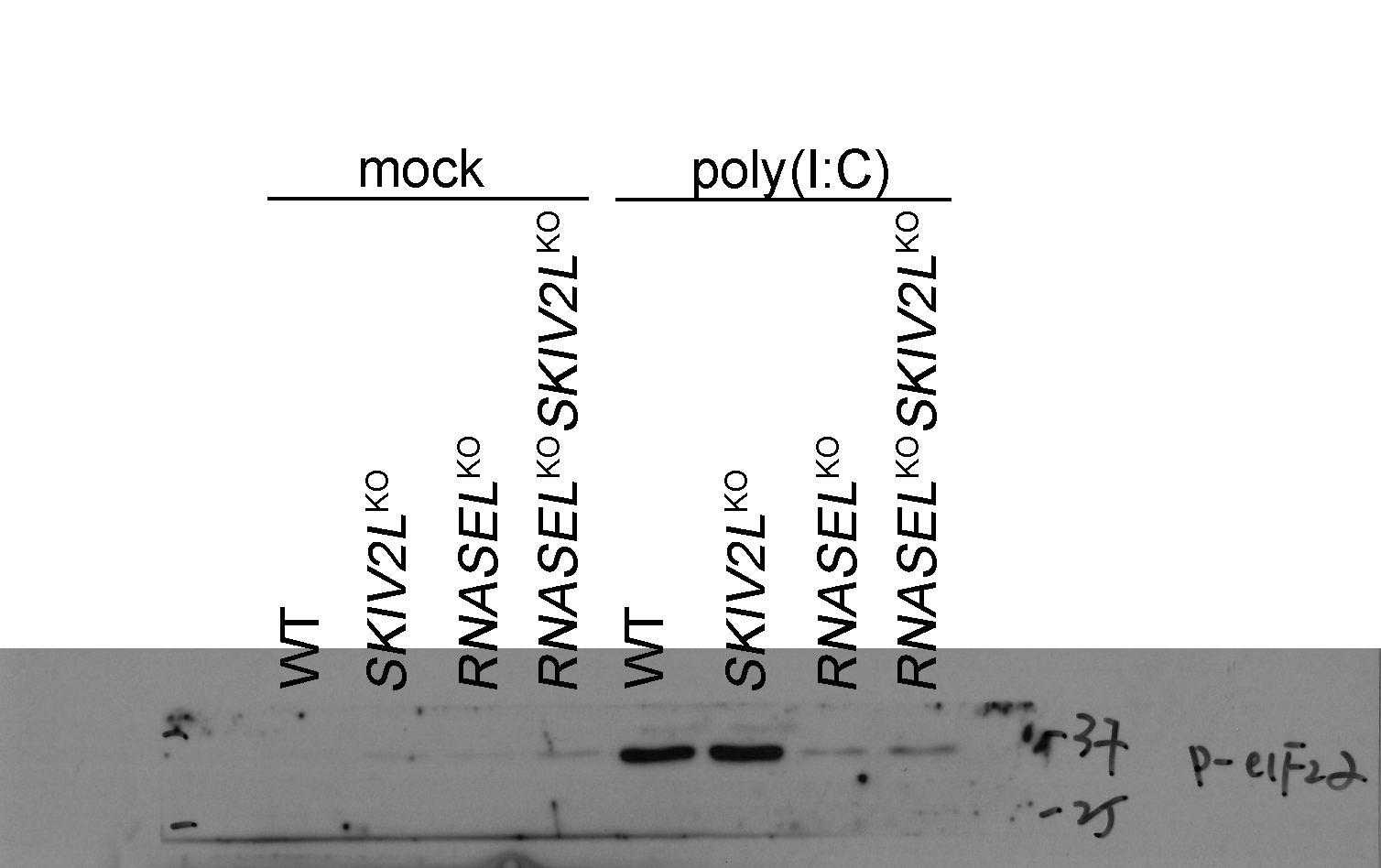

Supplement: Supplementary file 4 — Source data Fig. 3 [file 44318_2024_187_MOESM4_ESM.zip › Figure3/3A/p-eIF2a.tif]

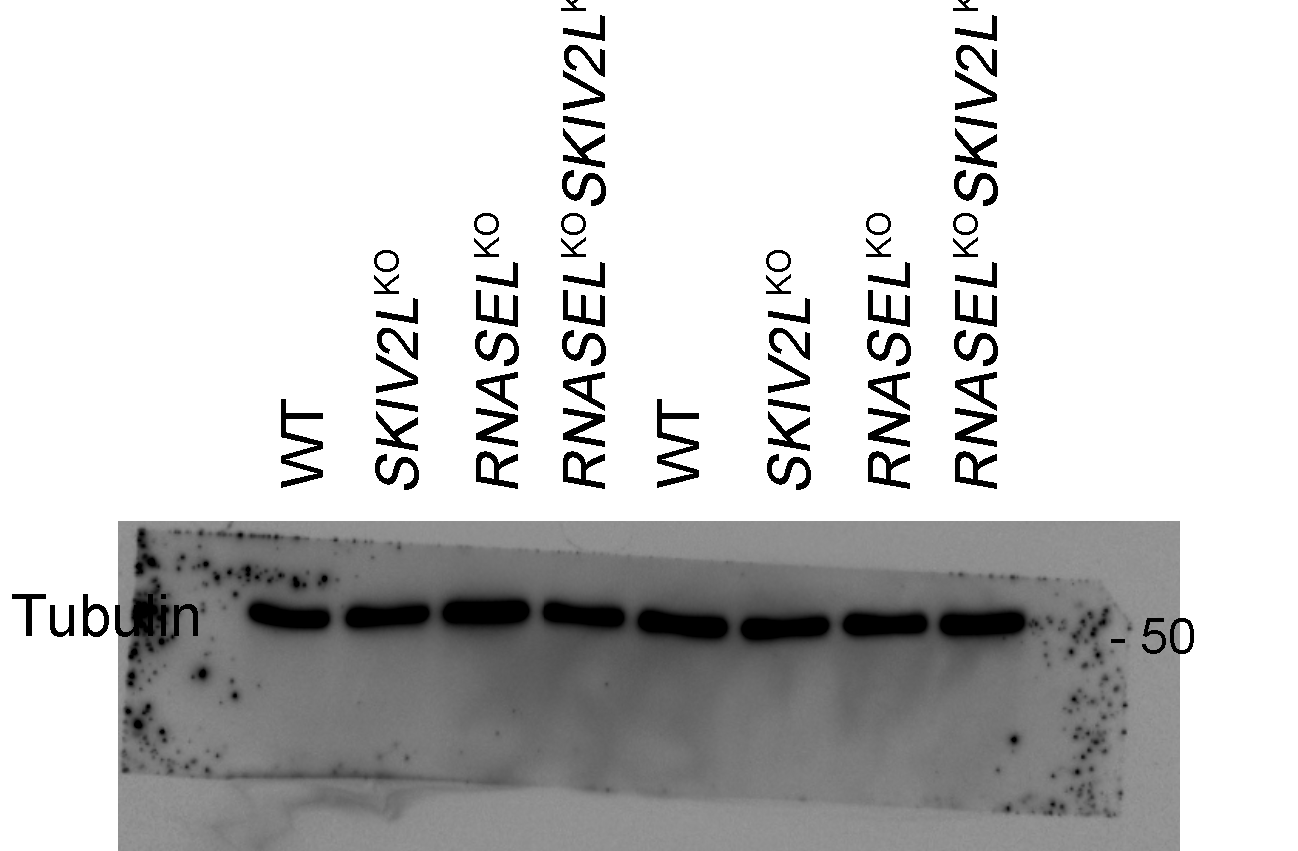

Supplement: Supplementary file 4 — Source data Fig. 3 [file 44318_2024_187_MOESM4_ESM.zip › Figure3/3A/tubulin.tif]

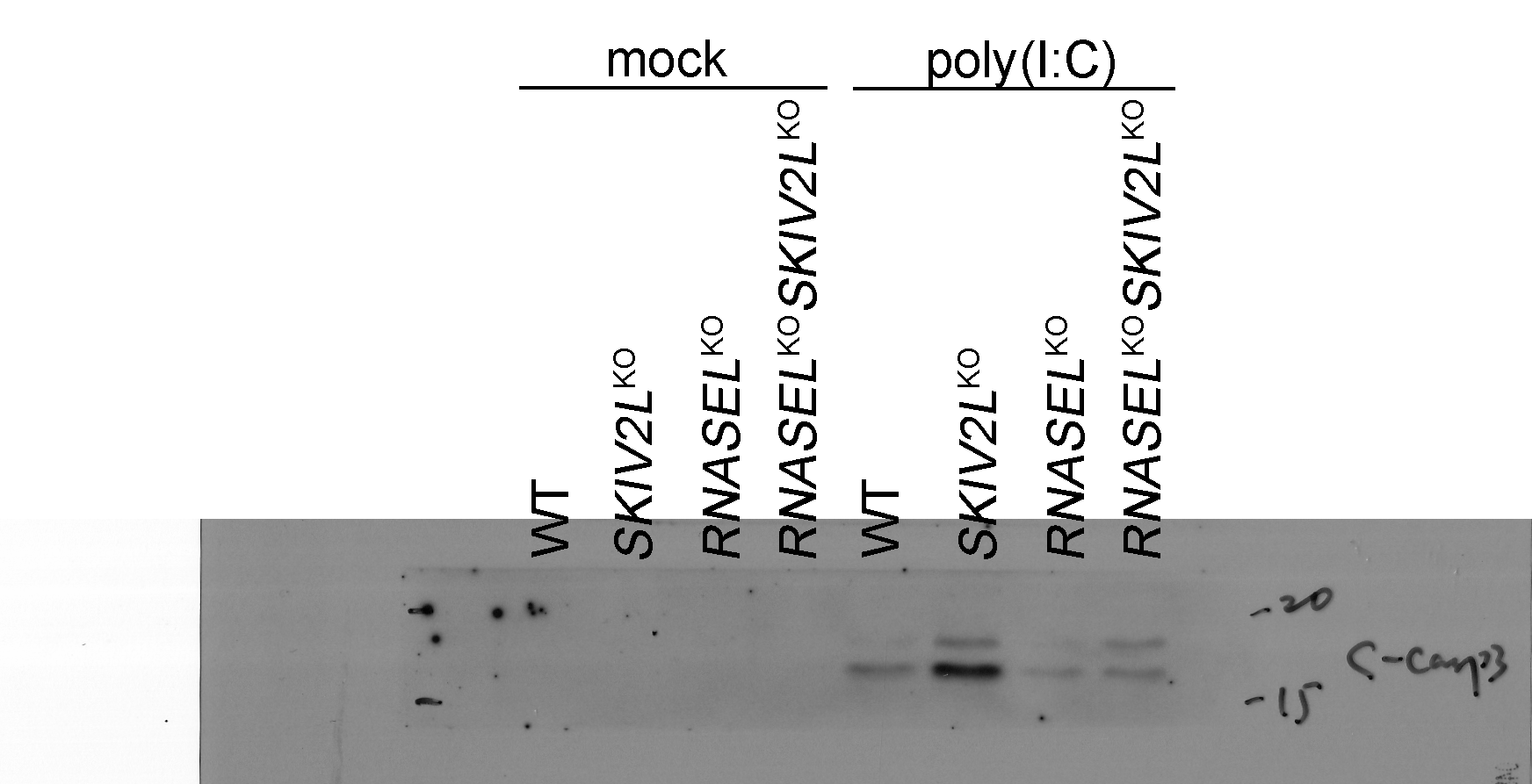

Supplement: Supplementary file 4 — Source data Fig. 3 [file 44318_2024_187_MOESM4_ESM.zip › Figure3/3A/c-Casp3.tif]

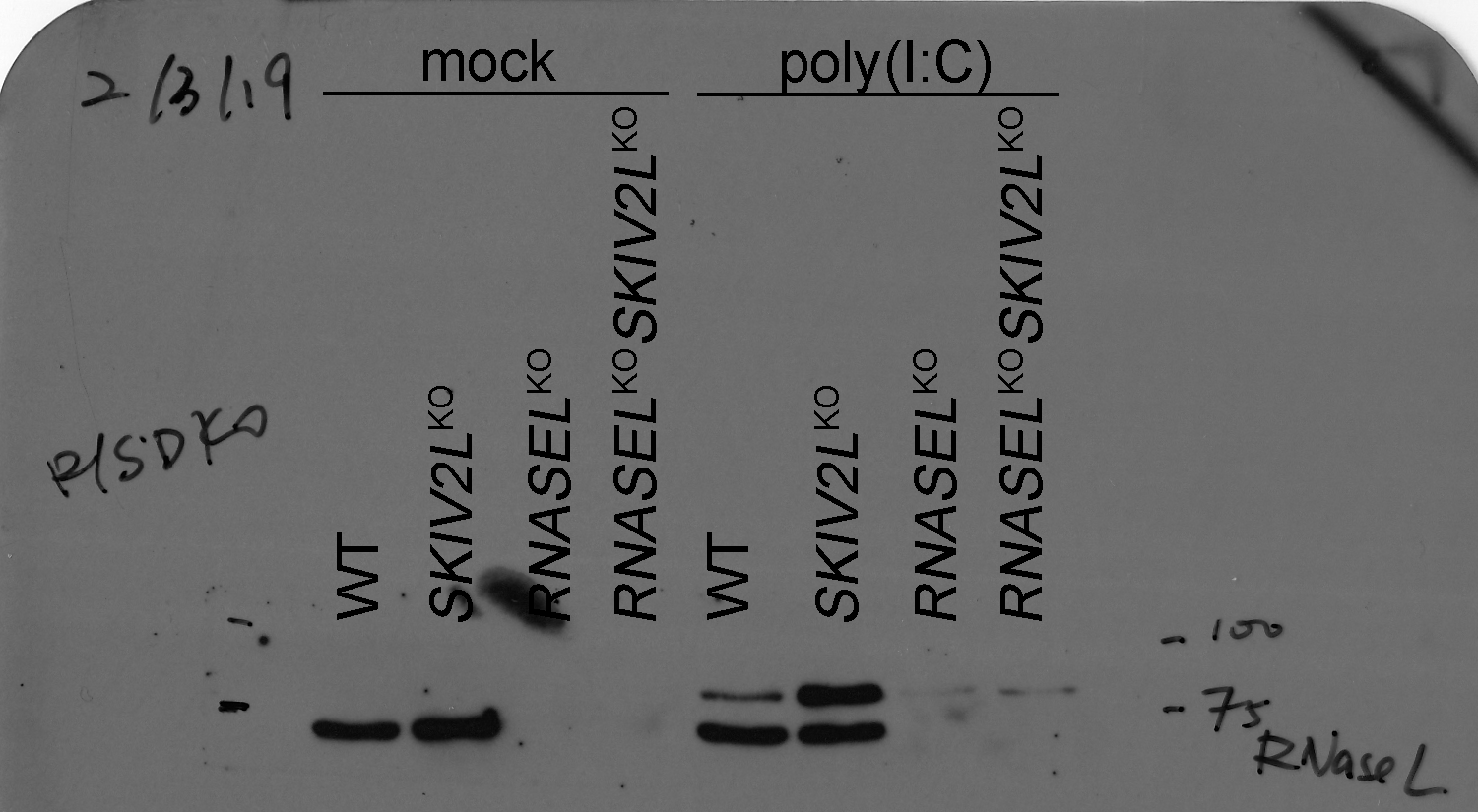

Supplement: Supplementary file 4 — Source data Fig. 3 [file 44318_2024_187_MOESM4_ESM.zip › Figure3/3A/RNase L.tif]

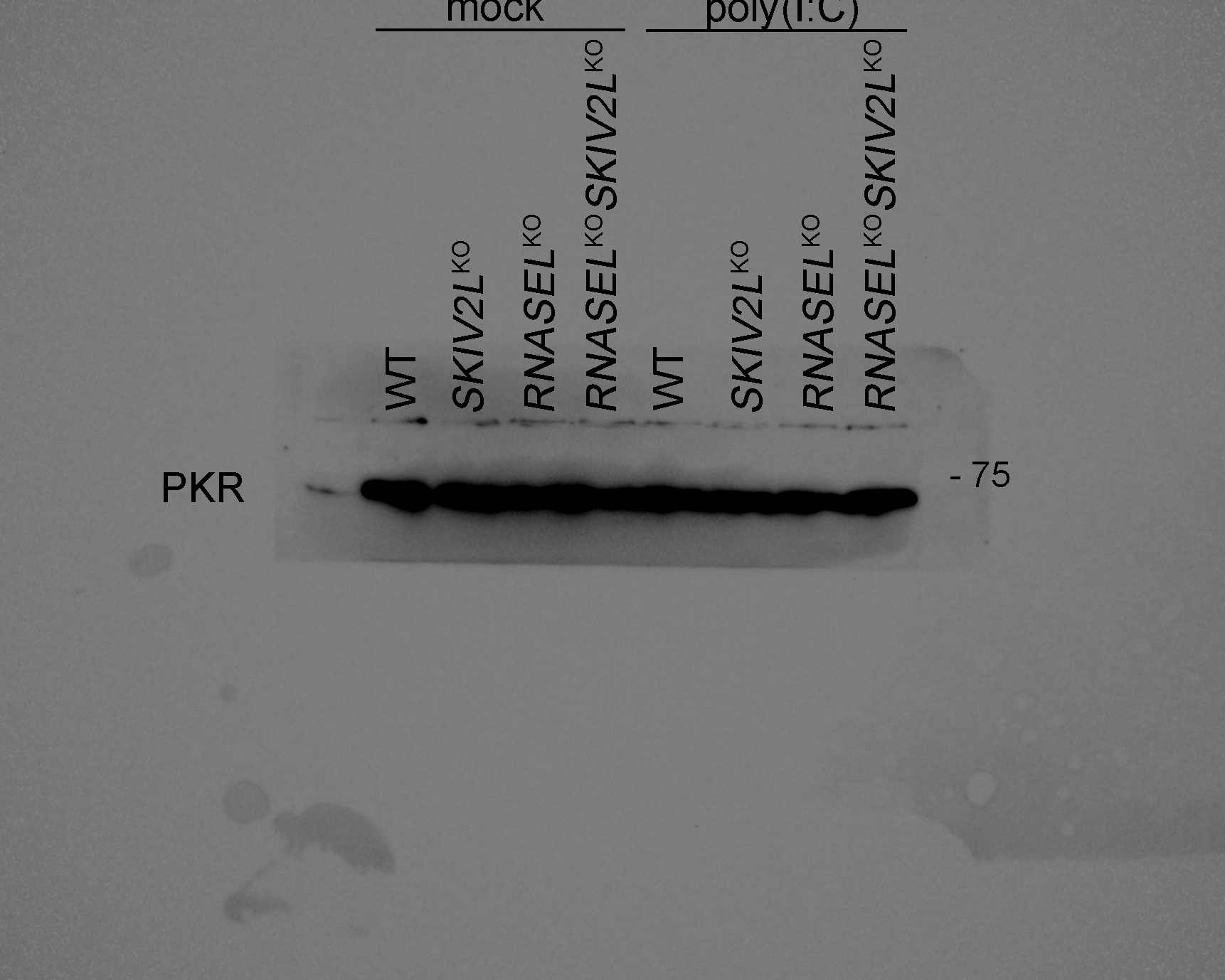

Supplement: Supplementary file 4 — Source data Fig. 3 [file 44318_2024_187_MOESM4_ESM.zip › Figure3/3A/PKR.tif]

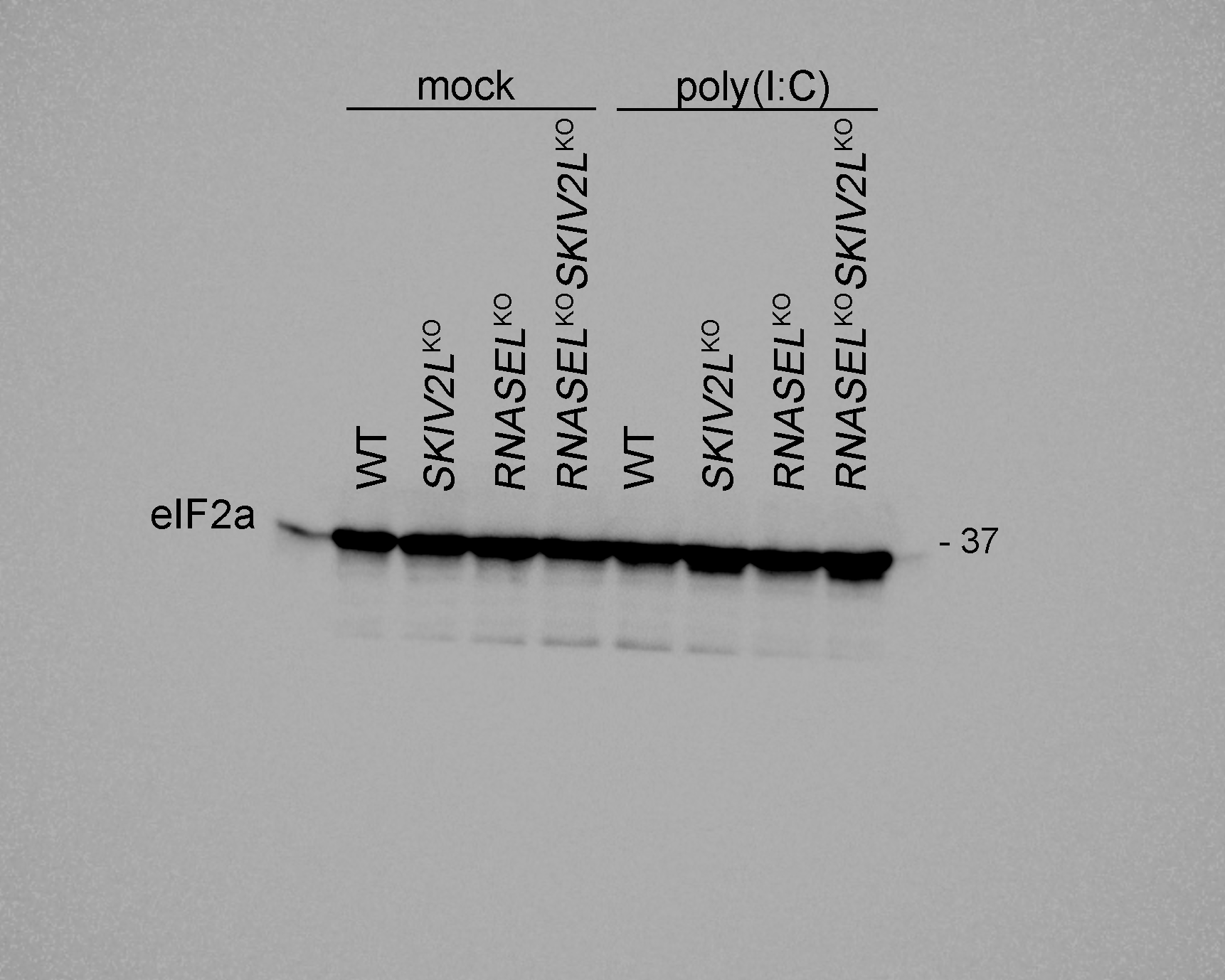

Supplement: Supplementary file 4 — Source data Fig. 3 [file 44318_2024_187_MOESM4_ESM.zip › Figure3/3A/eIF2a.tif]

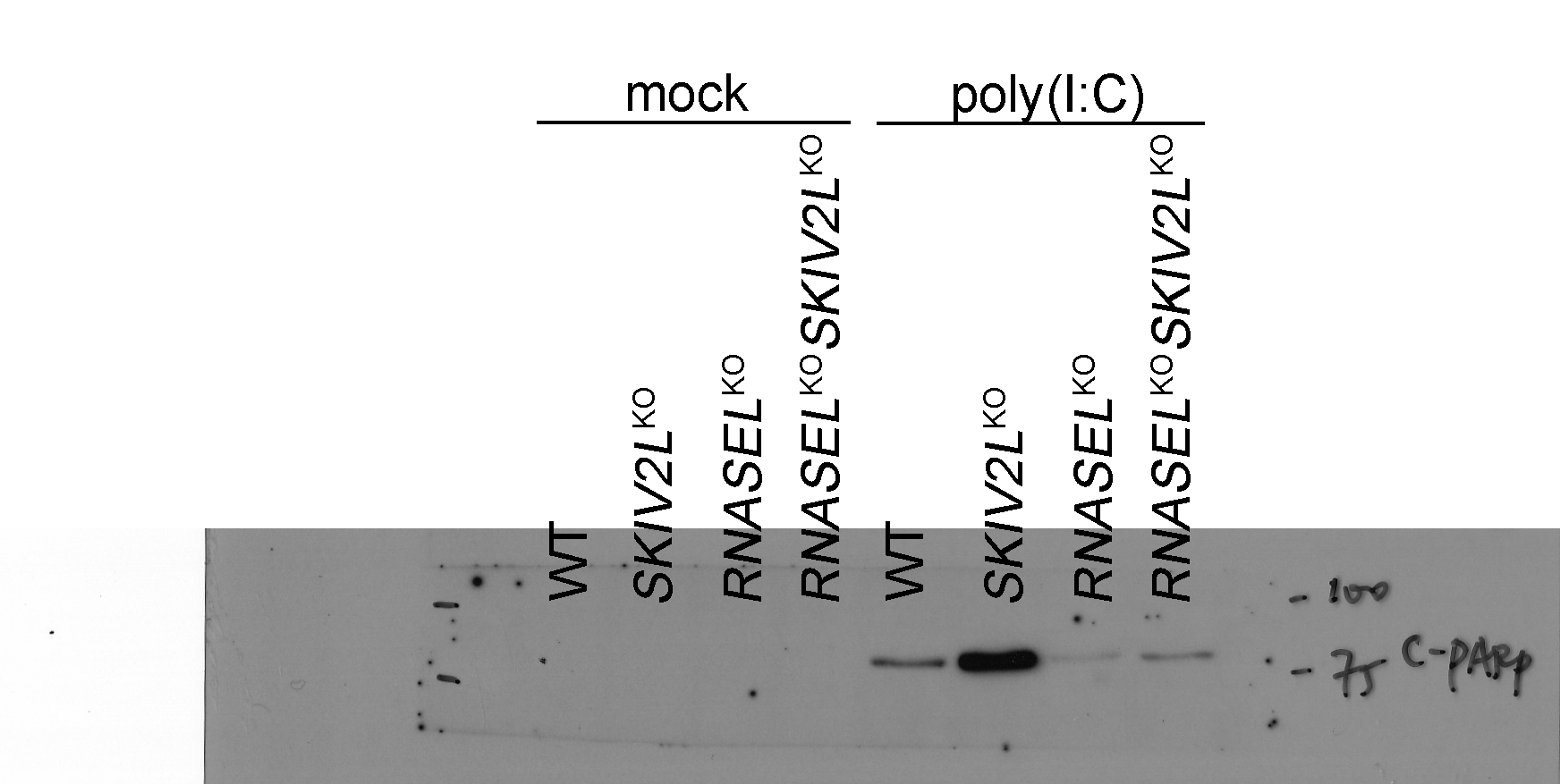

Supplement: Supplementary file 4 — Source data Fig. 3 [file 44318_2024_187_MOESM4_ESM.zip › Figure3/3A/c-PAPR.tif]

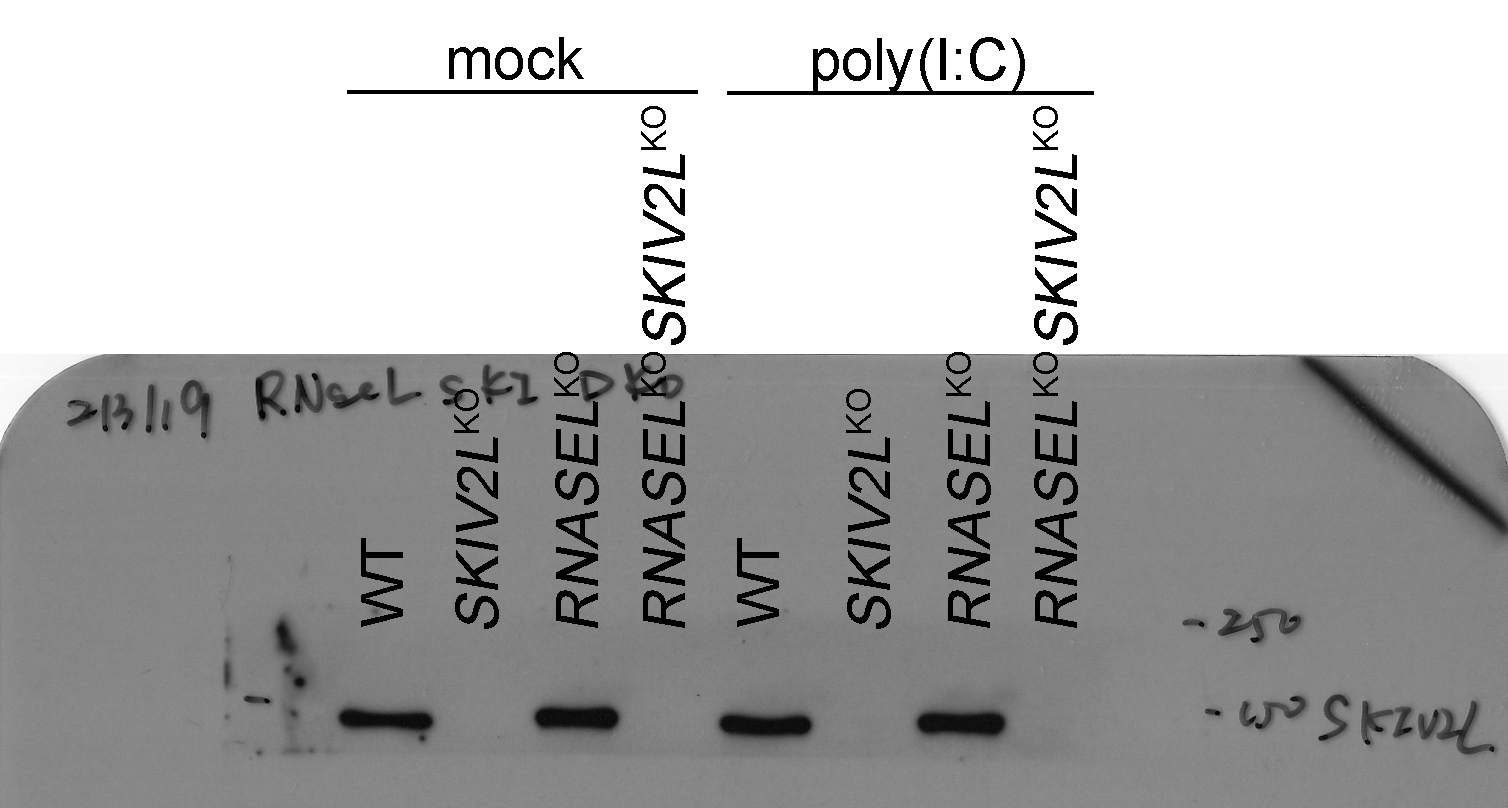

Supplement: Supplementary file 4 — Source data Fig. 3 [file 44318_2024_187_MOESM4_ESM.zip › Figure3/3A/SKIV2L.tif]

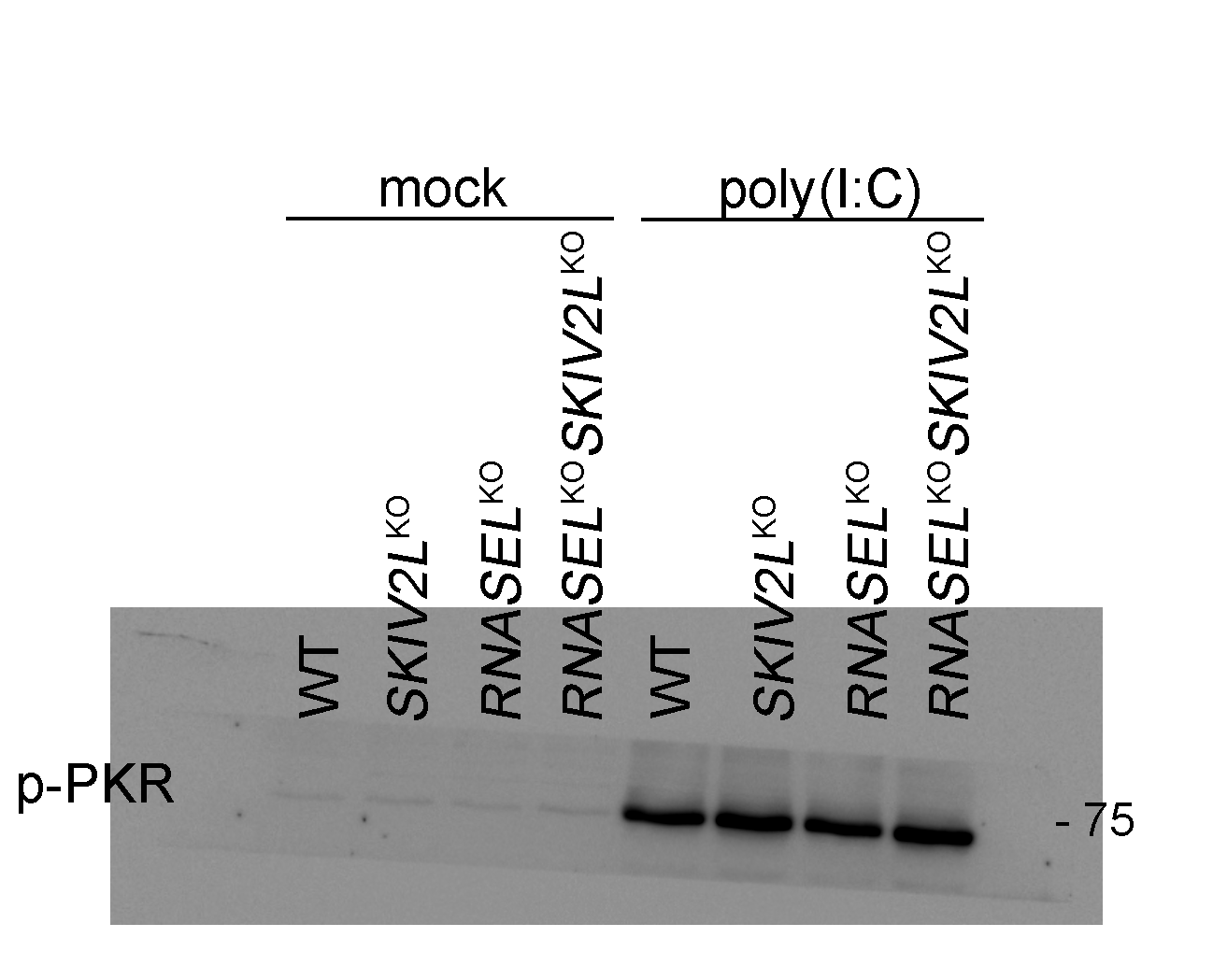

Supplement: Supplementary file 4 — Source data Fig. 3 [file 44318_2024_187_MOESM4_ESM.zip › Figure3/3A/p-PKR.tif]

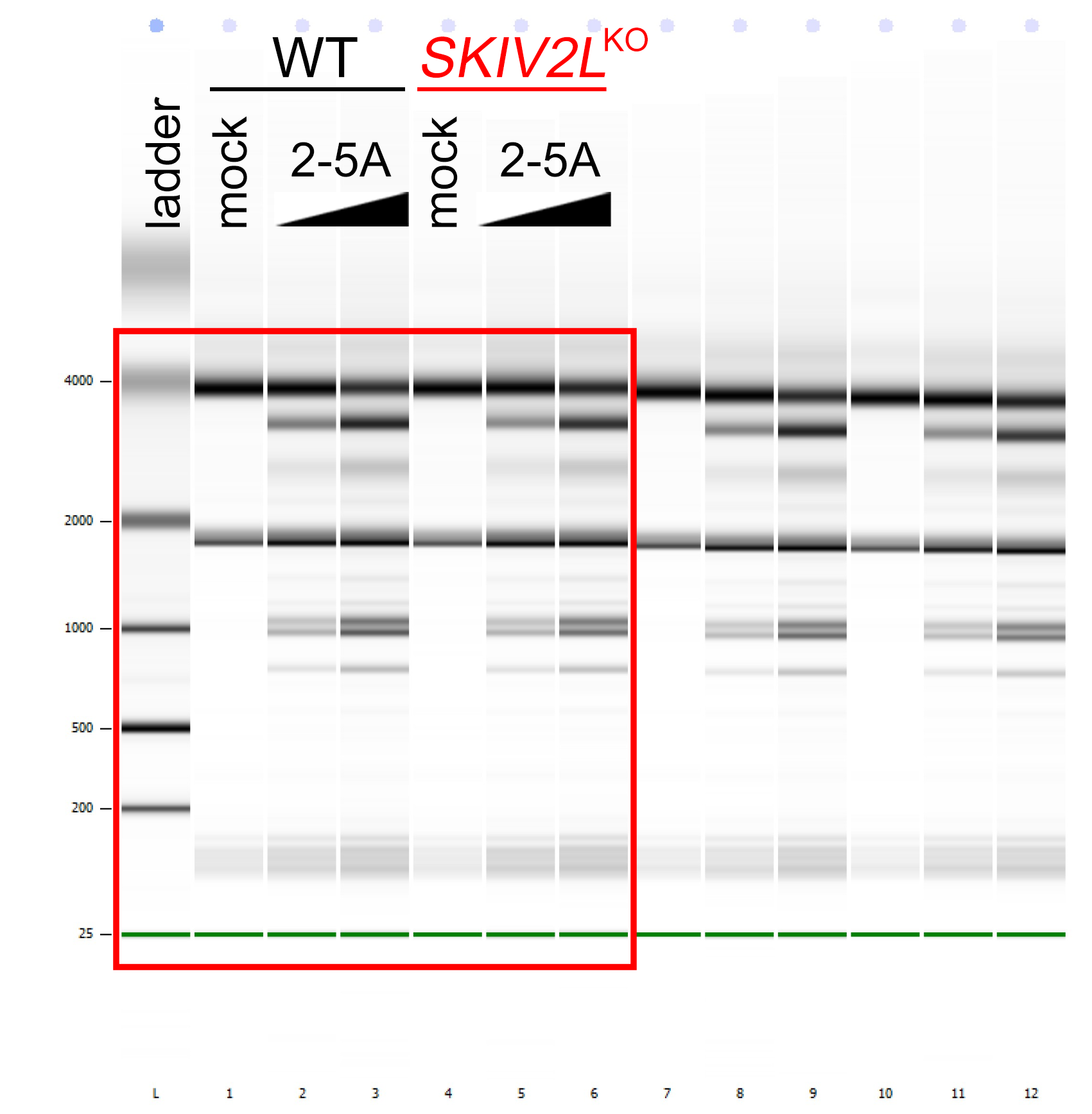

Supplement: Supplementary file 5 — Source data Fig. 4 [file 44318_2024_187_MOESM5_ESM.zip › Figure4/4E/Bioanalyzer.tif]

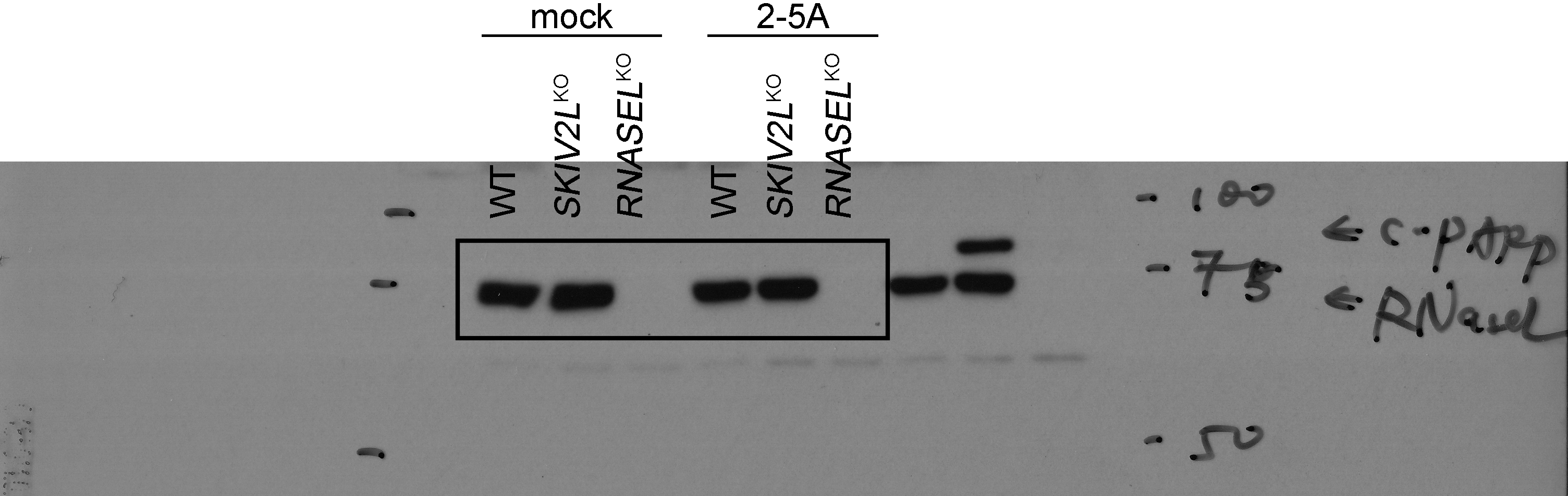

Supplement: Supplementary file 5 — Source data Fig. 4 [file 44318_2024_187_MOESM5_ESM.zip › Figure4/4B/RNaseL.tif]

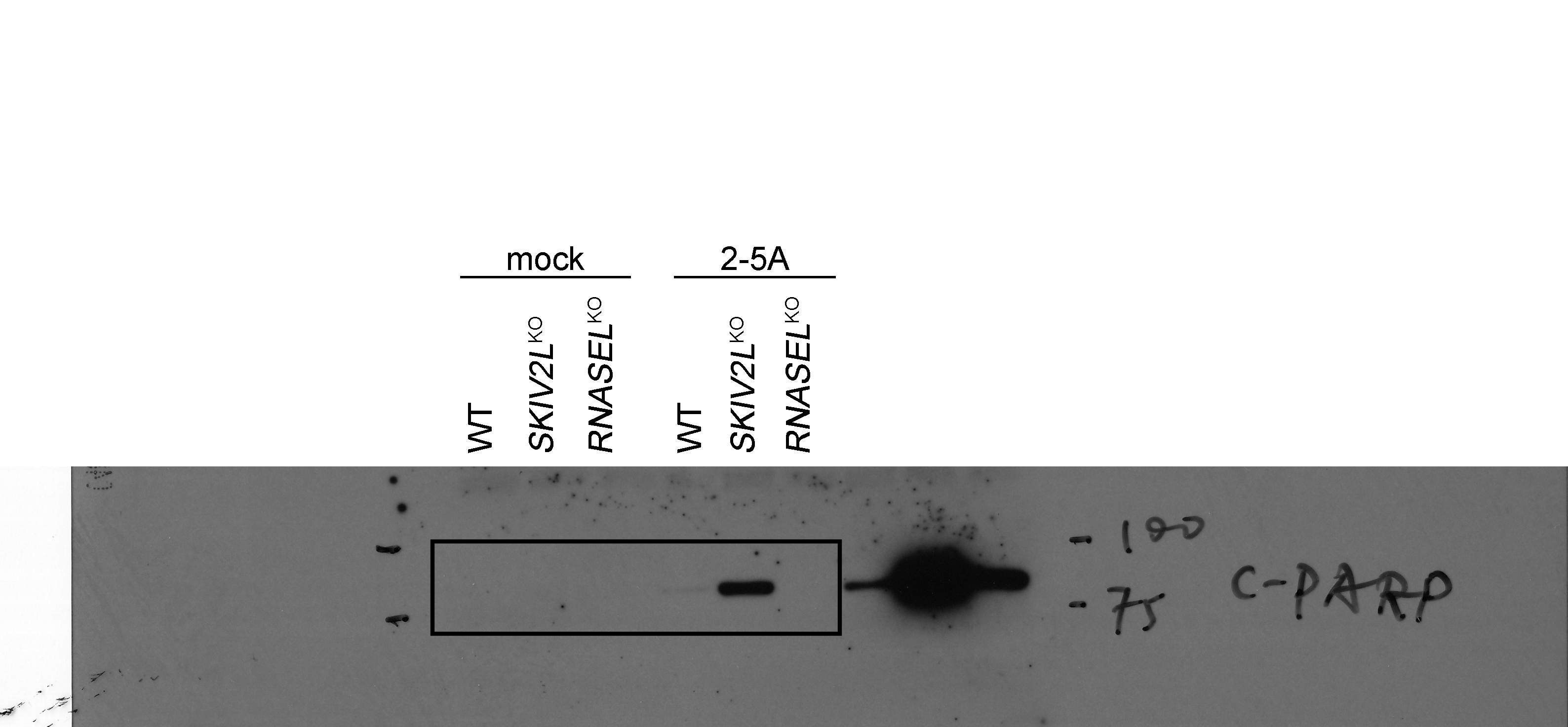

Supplement: Supplementary file 5 — Source data Fig. 4 [file 44318_2024_187_MOESM5_ESM.zip › Figure4/4B/c-PARP.tif]

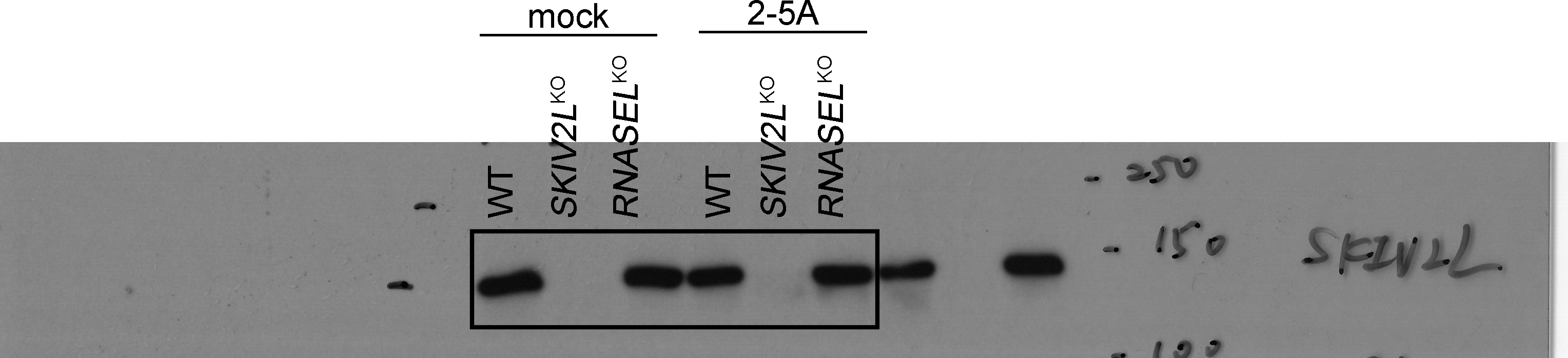

Supplement: Supplementary file 5 — Source data Fig. 4 [file 44318_2024_187_MOESM5_ESM.zip › Figure4/4B/SKIV2L.tif]

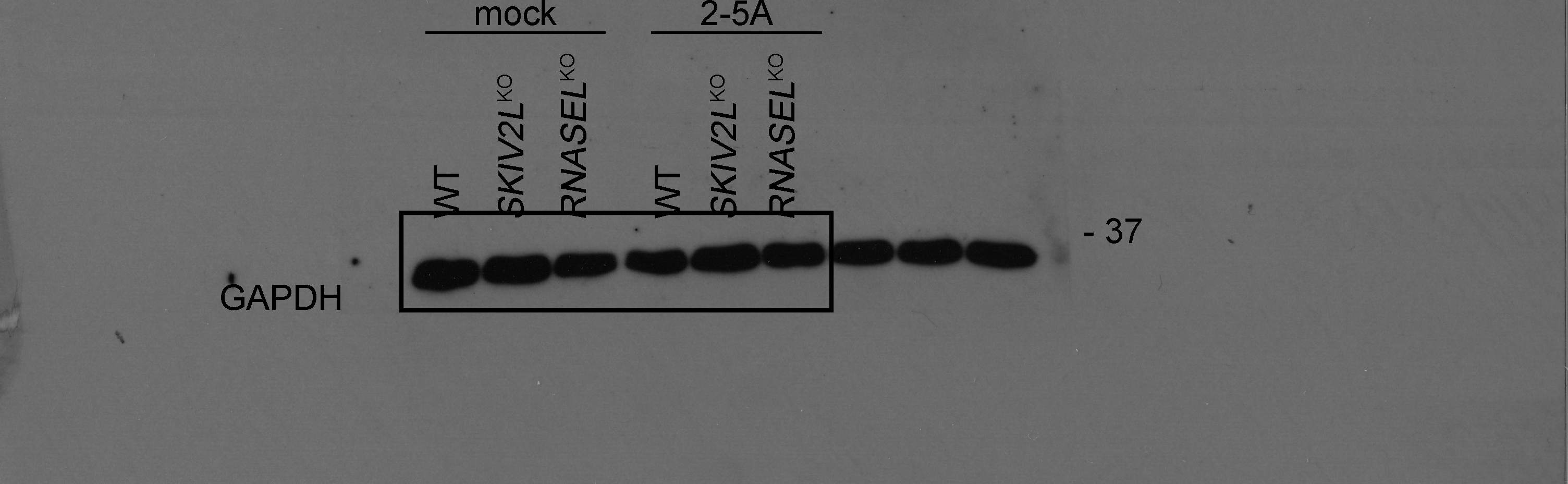

Supplement: Supplementary file 5 — Source data Fig. 4 [file 44318_2024_187_MOESM5_ESM.zip › Figure4/4B/GAPDH.tif]

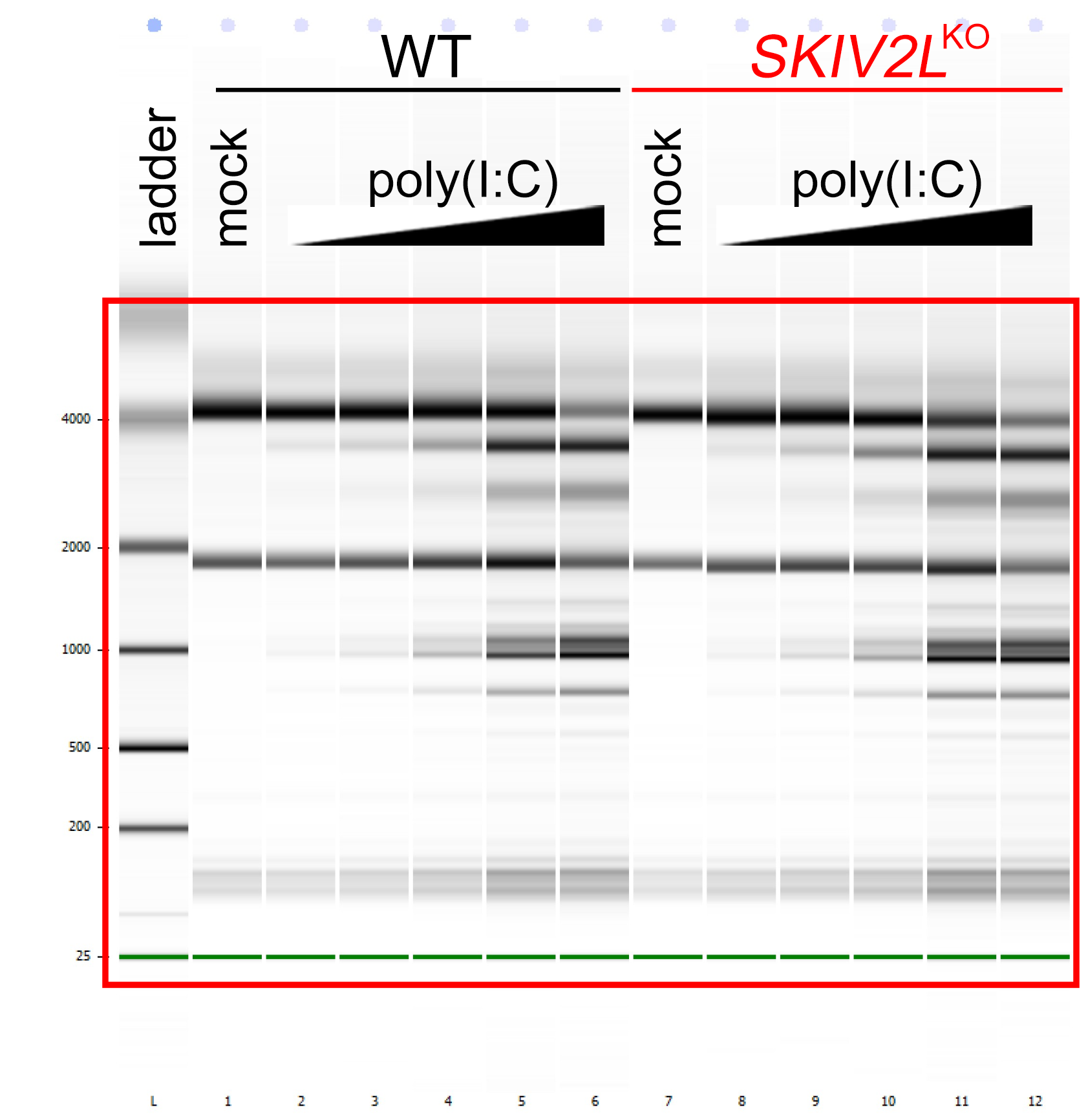

Supplement: Supplementary file 5 — Source data Fig. 4 [file 44318_2024_187_MOESM5_ESM.zip › Figure4/4D/Bioanalyzer.tif]

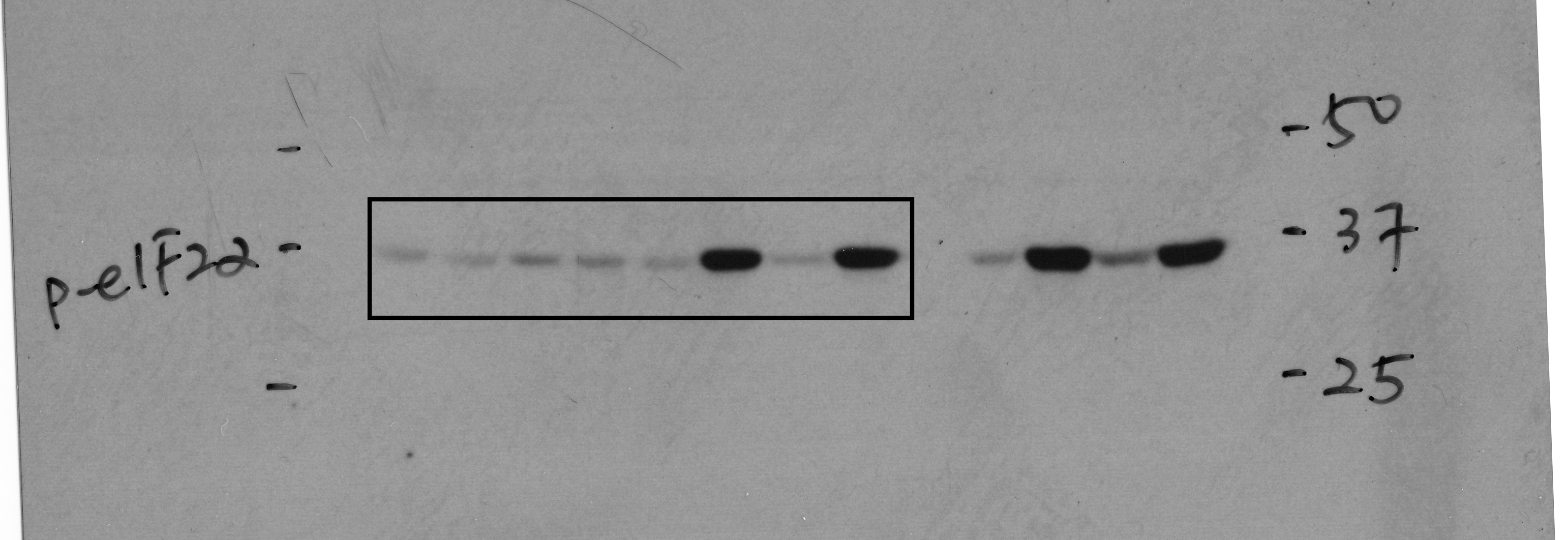

Supplement: Supplementary file 7 — Source data Fig. 6 [file 44318_2024_187_MOESM7_ESM.zip › Figure6/6H/p-eIF2a.tif]

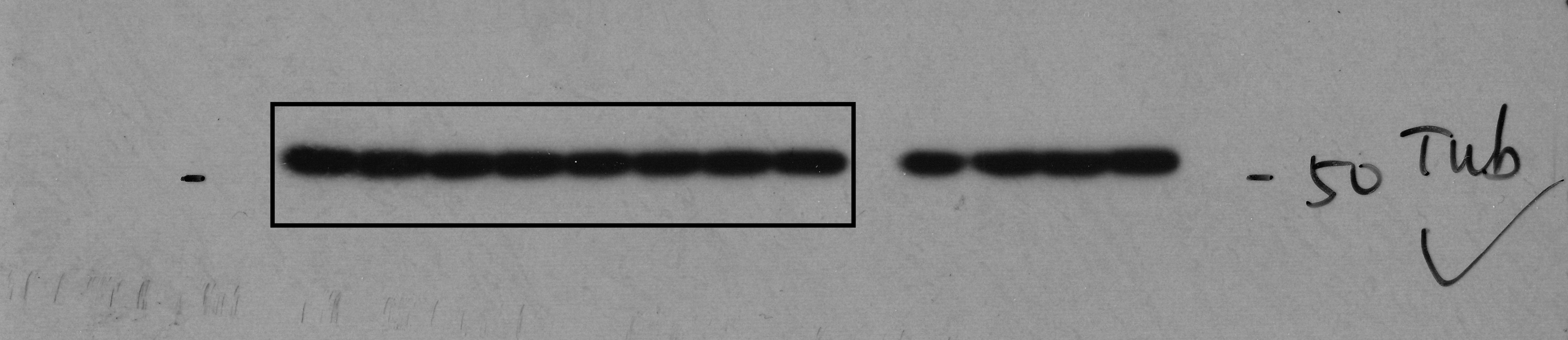

Supplement: Supplementary file 7 — Source data Fig. 6 [file 44318_2024_187_MOESM7_ESM.zip › Figure6/6H/Tubulin.tif]

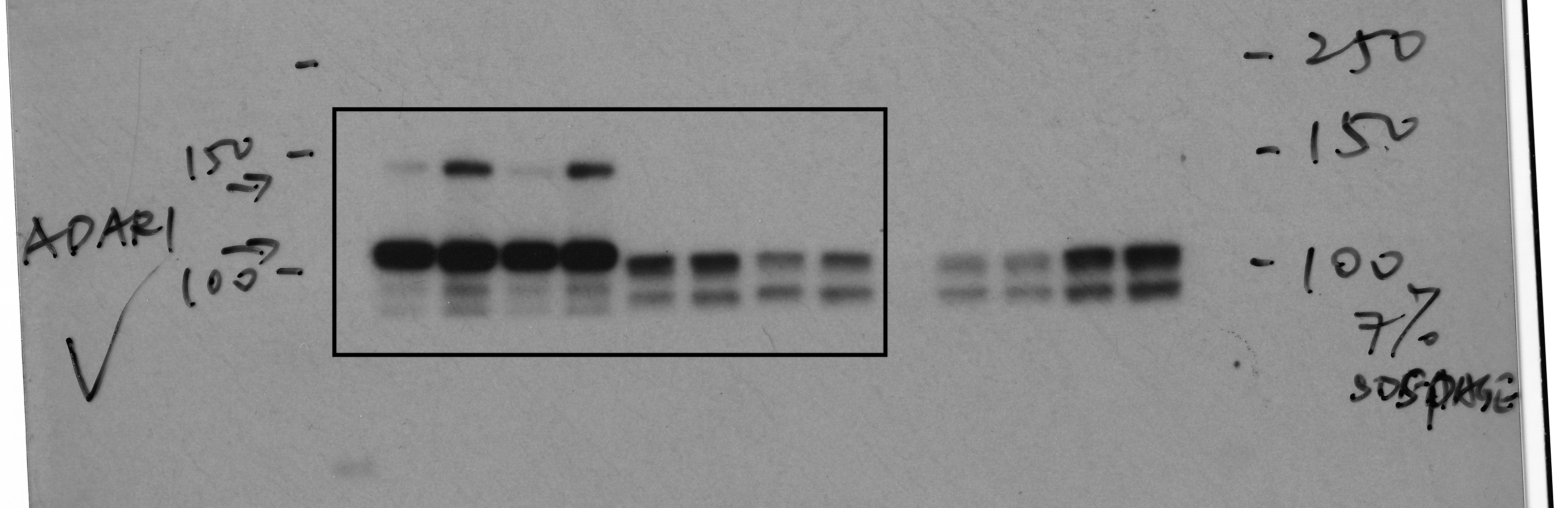

Supplement: Supplementary file 7 — Source data Fig. 6 [file 44318_2024_187_MOESM7_ESM.zip › Figure6/6H/ADAR1.tif]

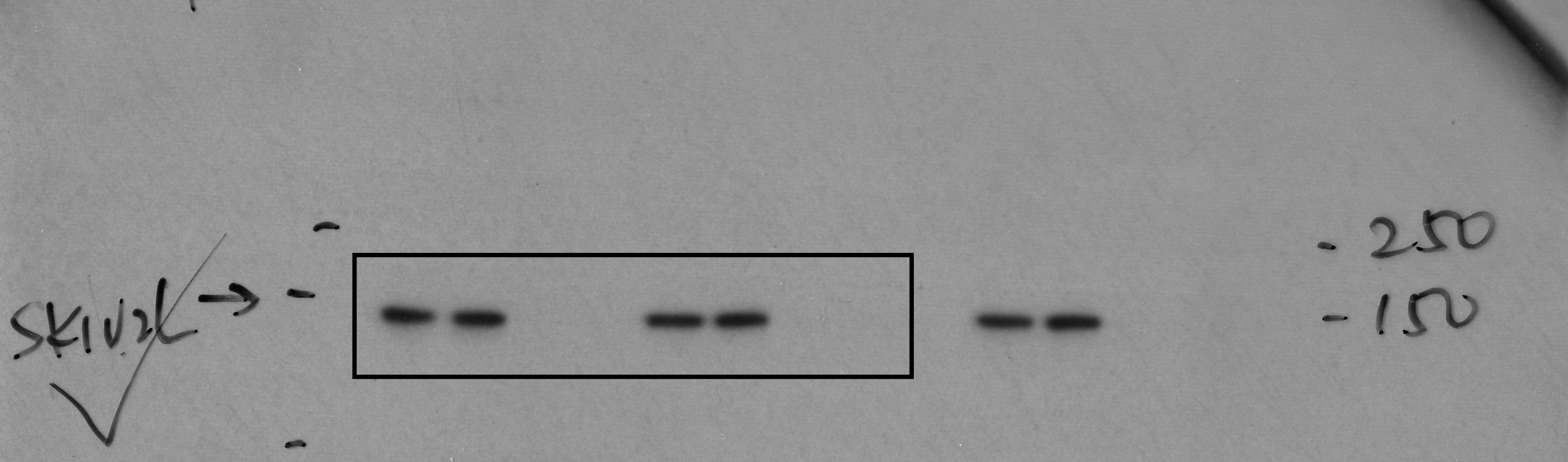

Supplement: Supplementary file 7 — Source data Fig. 6 [file 44318_2024_187_MOESM7_ESM.zip › Figure6/6H/SKIV2L.tif]

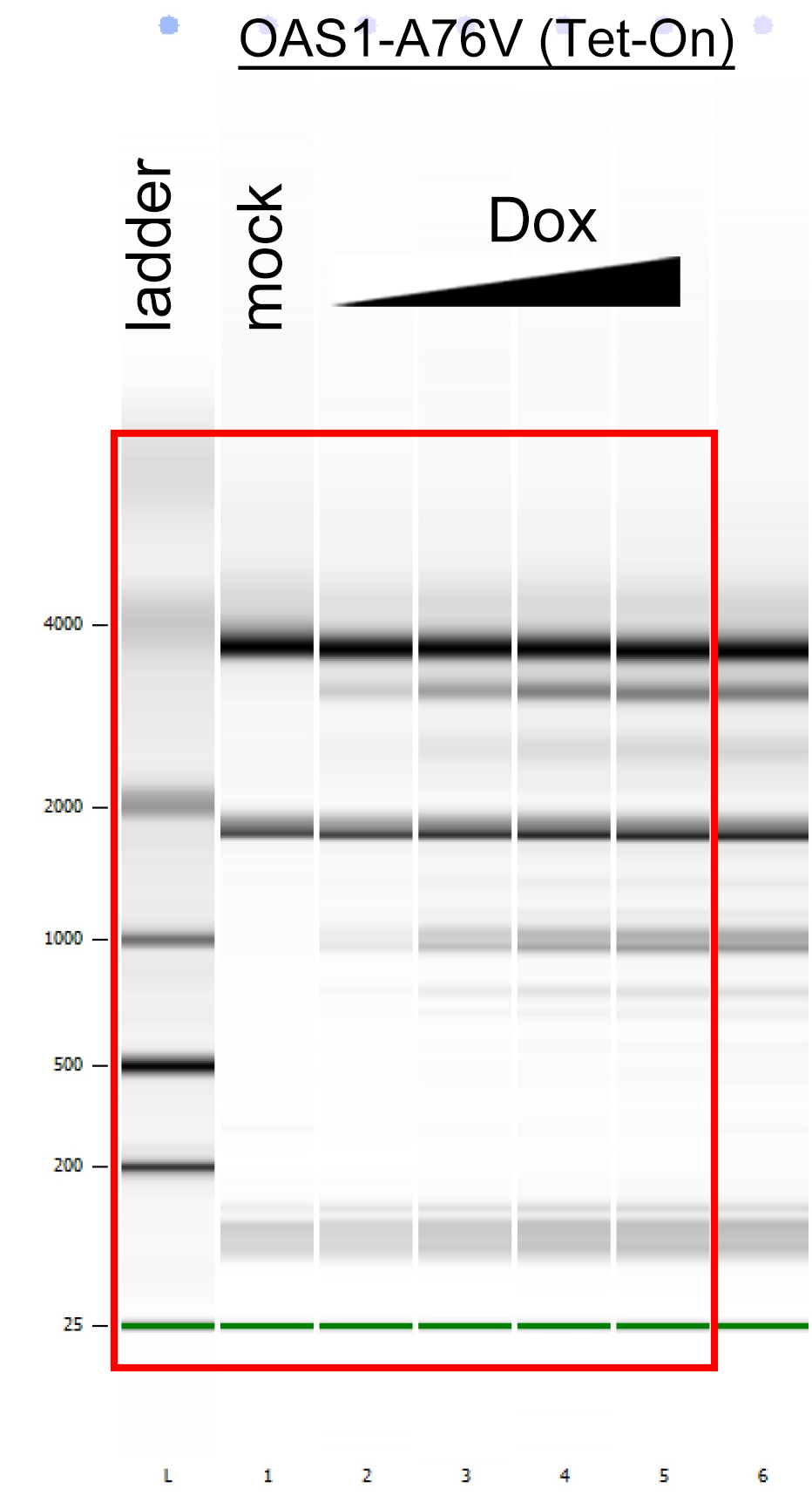

Supplement: Supplementary file 7 — Source data Fig. 6 [file 44318_2024_187_MOESM7_ESM.zip › Figure6/6B/Bioanalyzer.tif]

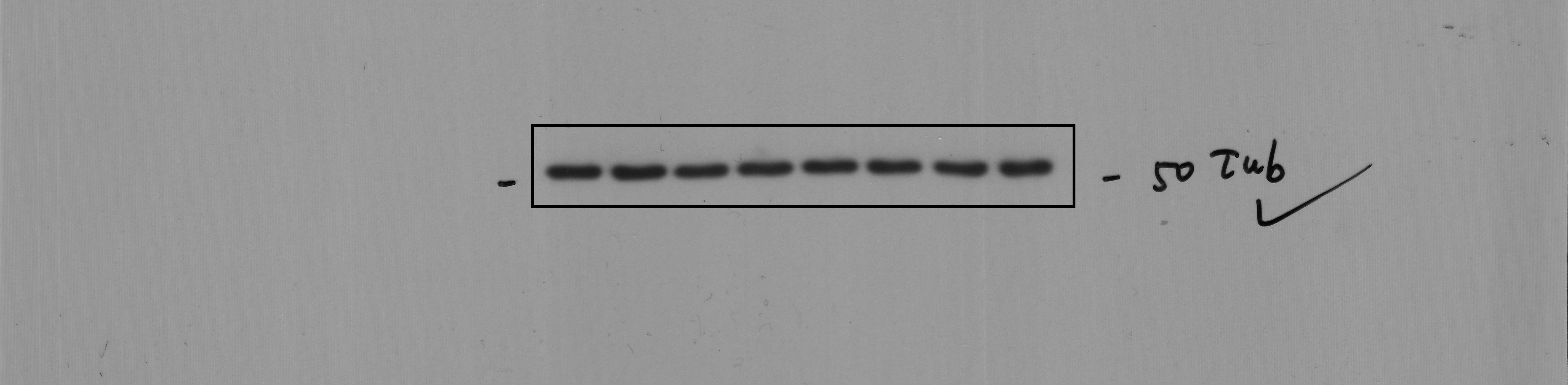

Supplement: Supplementary file 7 — Source data Fig. 6 [file 44318_2024_187_MOESM7_ESM.zip › Figure6/6D/Tubulin.tif]

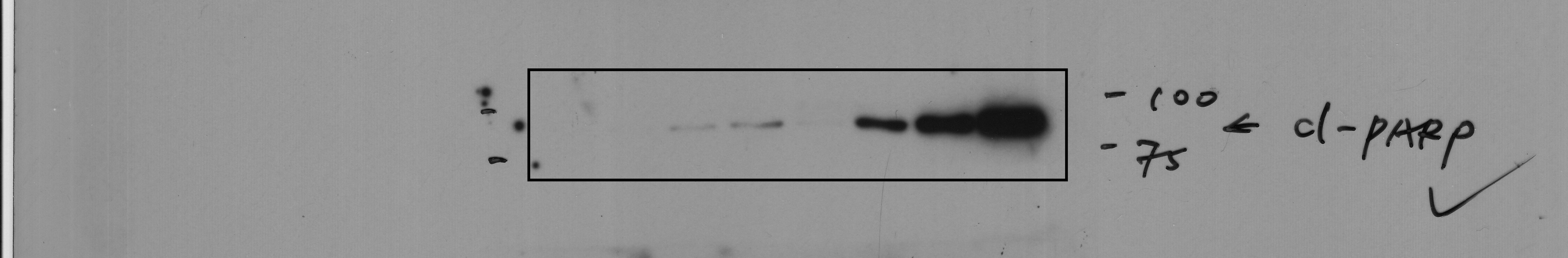

Supplement: Supplementary file 7 — Source data Fig. 6 [file 44318_2024_187_MOESM7_ESM.zip › Figure6/6D/cl PARP.tif]

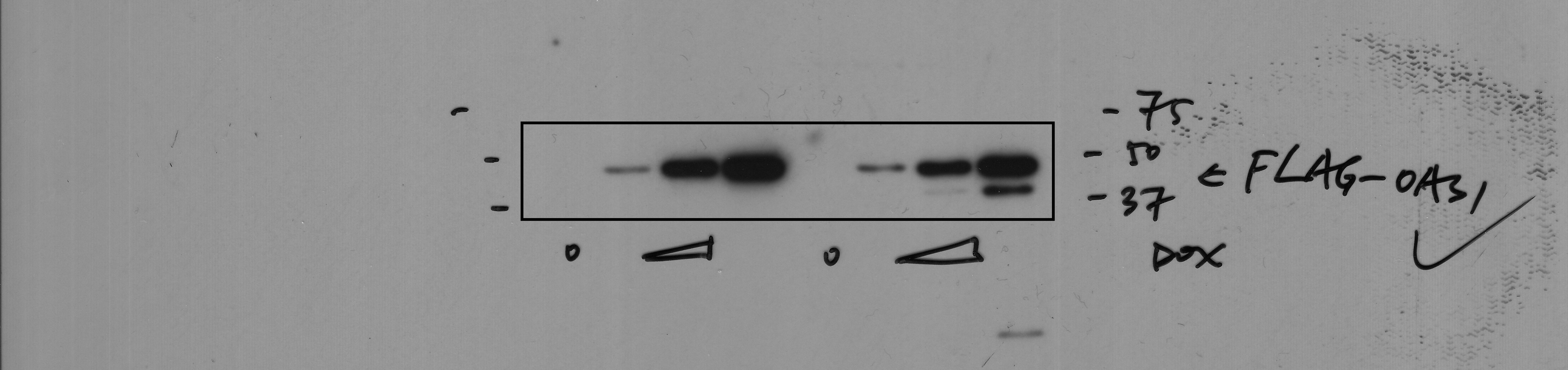

Supplement: Supplementary file 7 — Source data Fig. 6 [file 44318_2024_187_MOESM7_ESM.zip › Figure6/6D/FLAG-OAS1-A76V.tif]

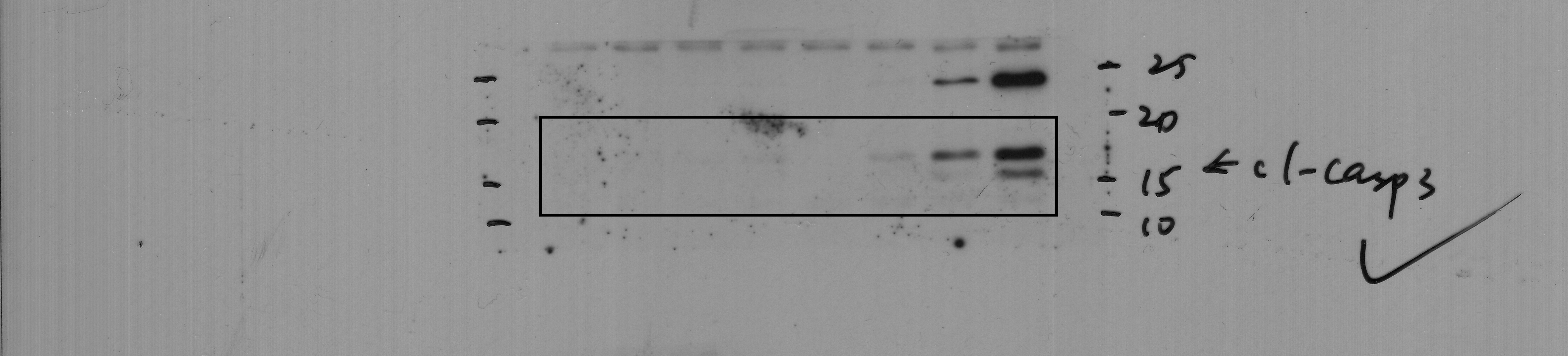

Supplement: Supplementary file 7 — Source data Fig. 6 [file 44318_2024_187_MOESM7_ESM.zip › Figure6/6D/cl-Casp3.tif]

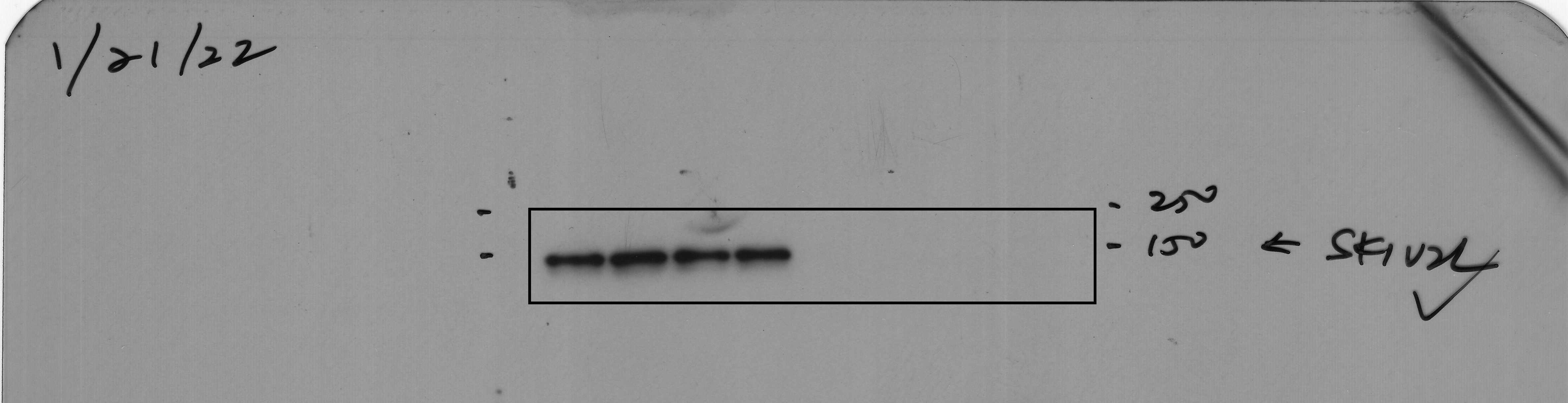

Supplement: Supplementary file 7 — Source data Fig. 6 [file 44318_2024_187_MOESM7_ESM.zip › Figure6/6D/SKIV2L.tif]

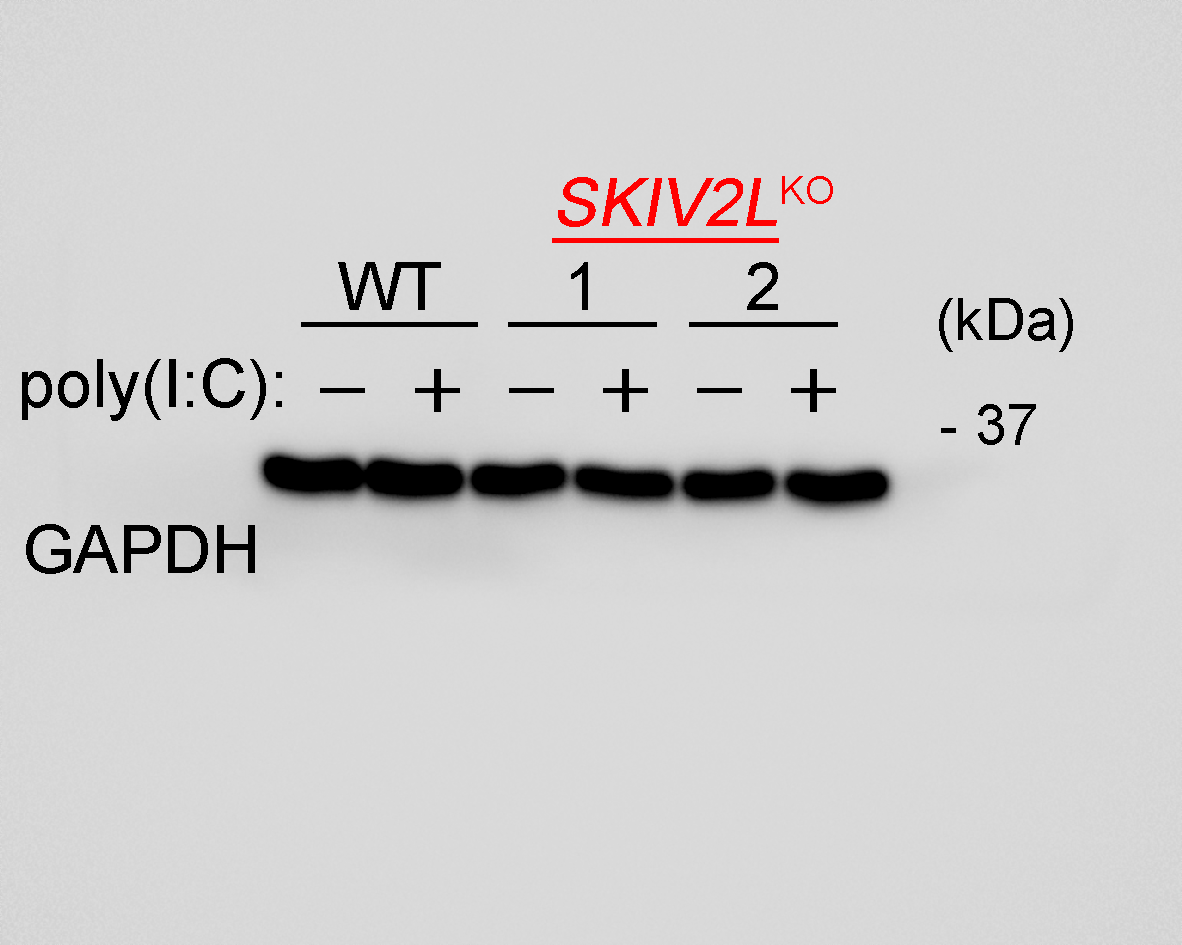

Supplement: Supplementary file 8 — EV Figure Source Data [file 44318_2024_187_MOESM8_ESM.zip › EV Figure1C/GAPDH.tif]

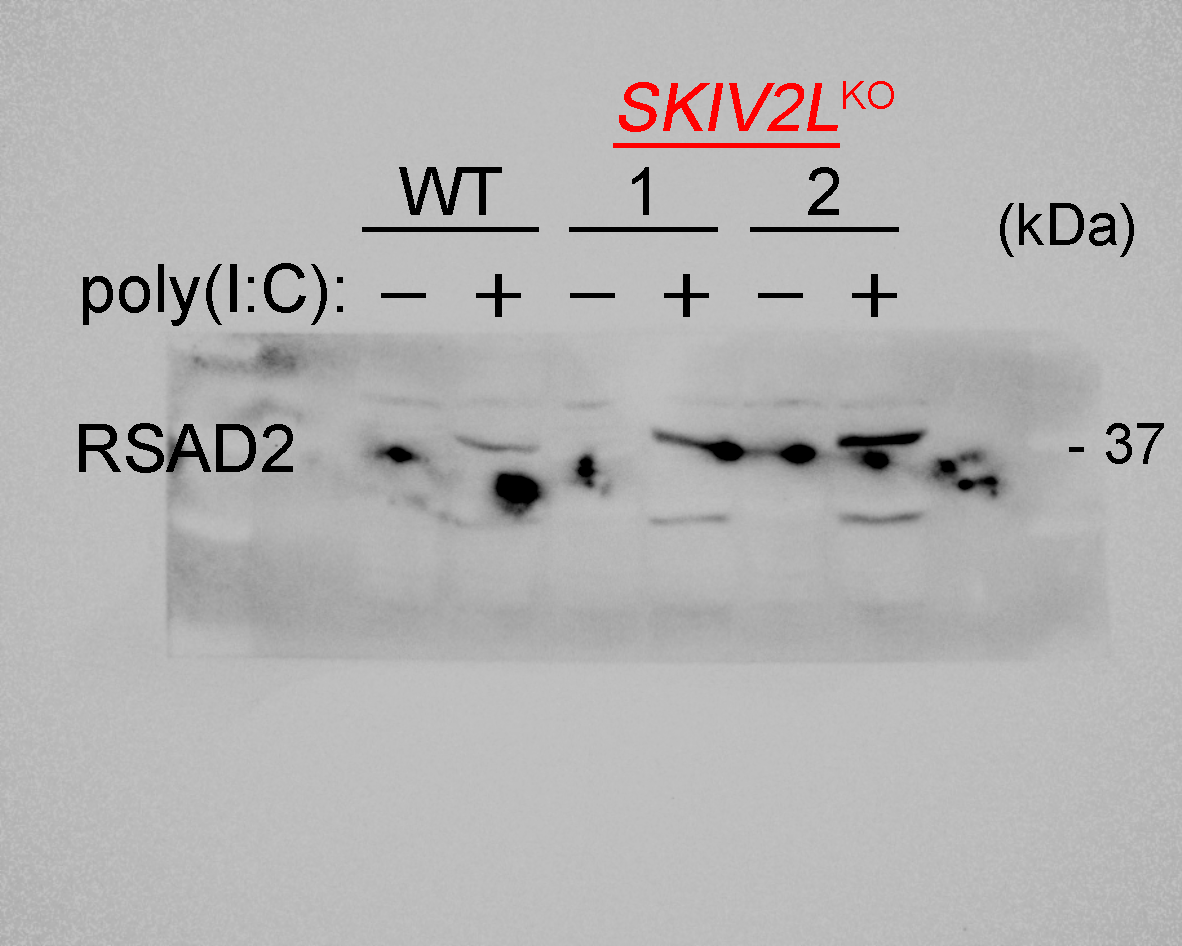

Supplement: Supplementary file 8 — EV Figure Source Data [file 44318_2024_187_MOESM8_ESM.zip › EV Figure1C/RSAD2.tif]

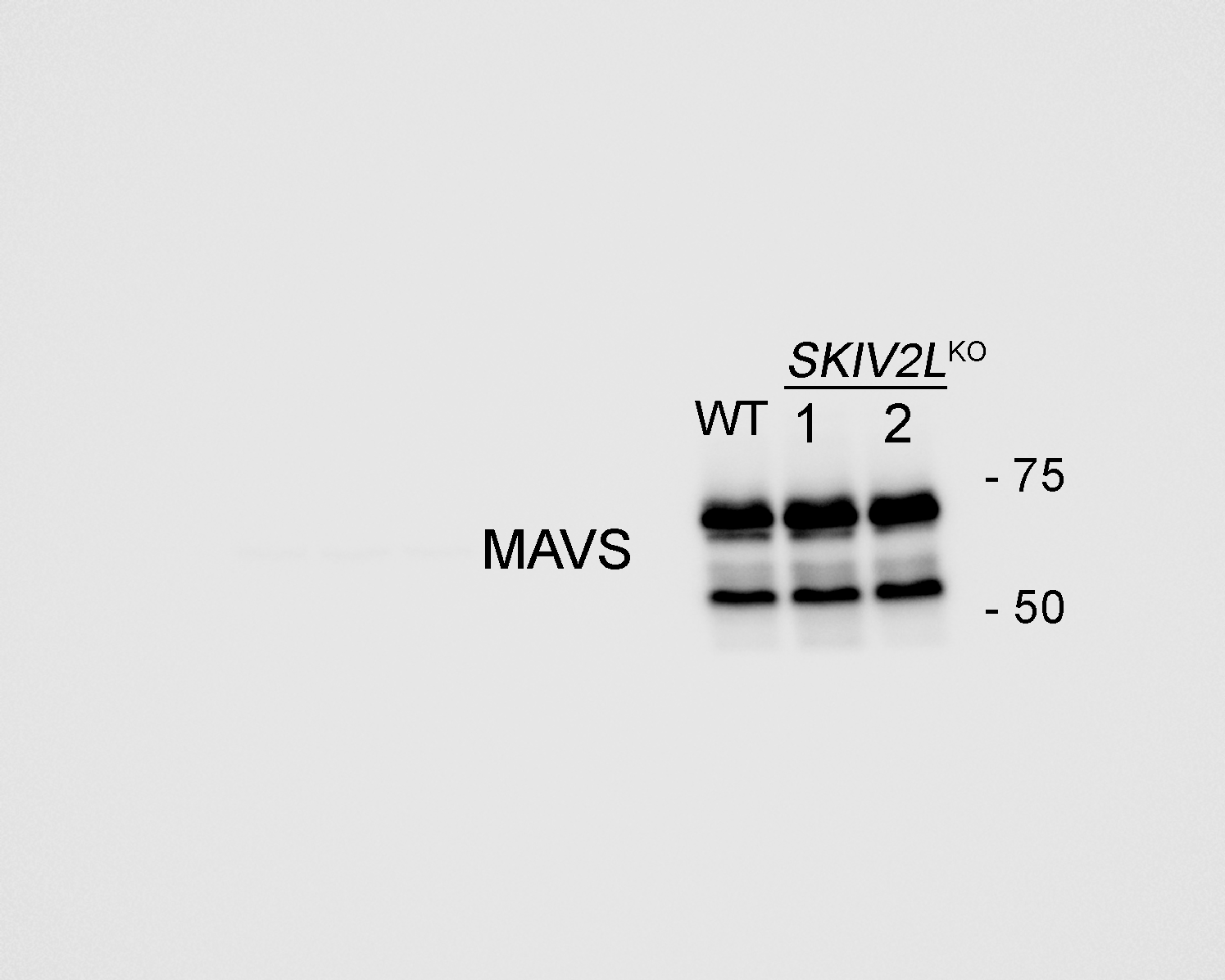

Supplement: Supplementary file 8 — EV Figure Source Data [file 44318_2024_187_MOESM8_ESM.zip › EV Figure1D/MAVS copy.tif]

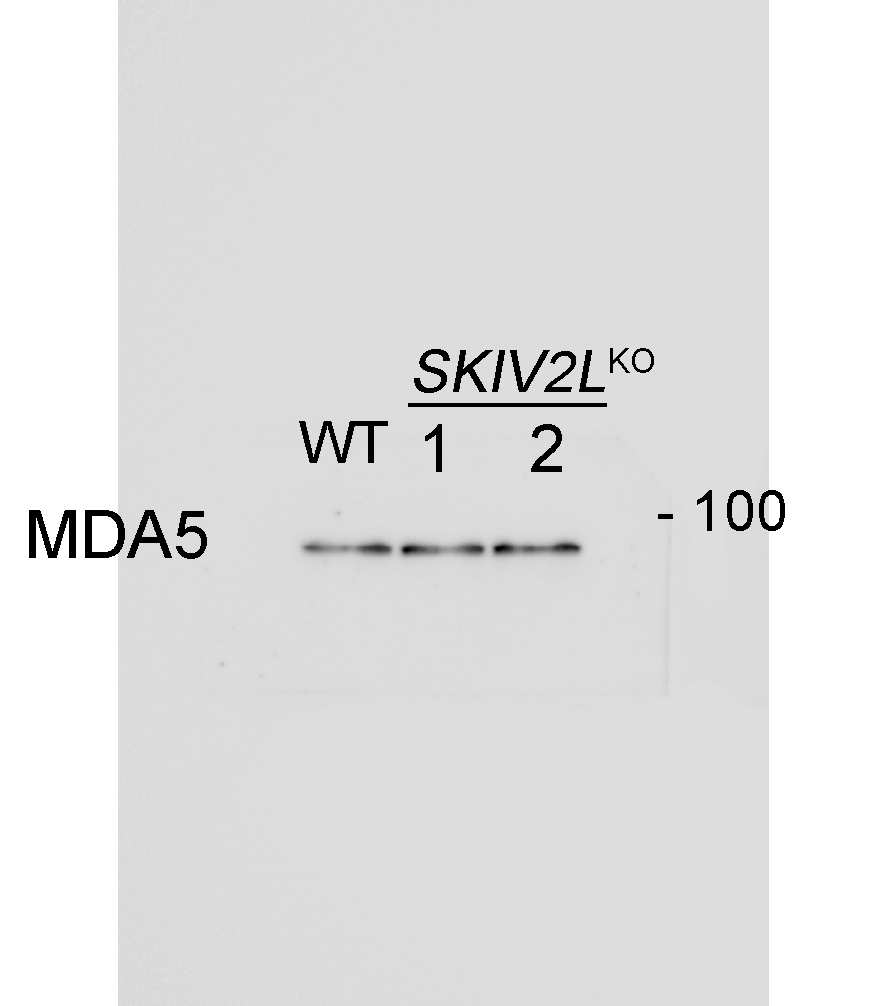

Supplement: Supplementary file 8 — EV Figure Source Data [file 44318_2024_187_MOESM8_ESM.zip › EV Figure1D/MDA5 copy.tif]

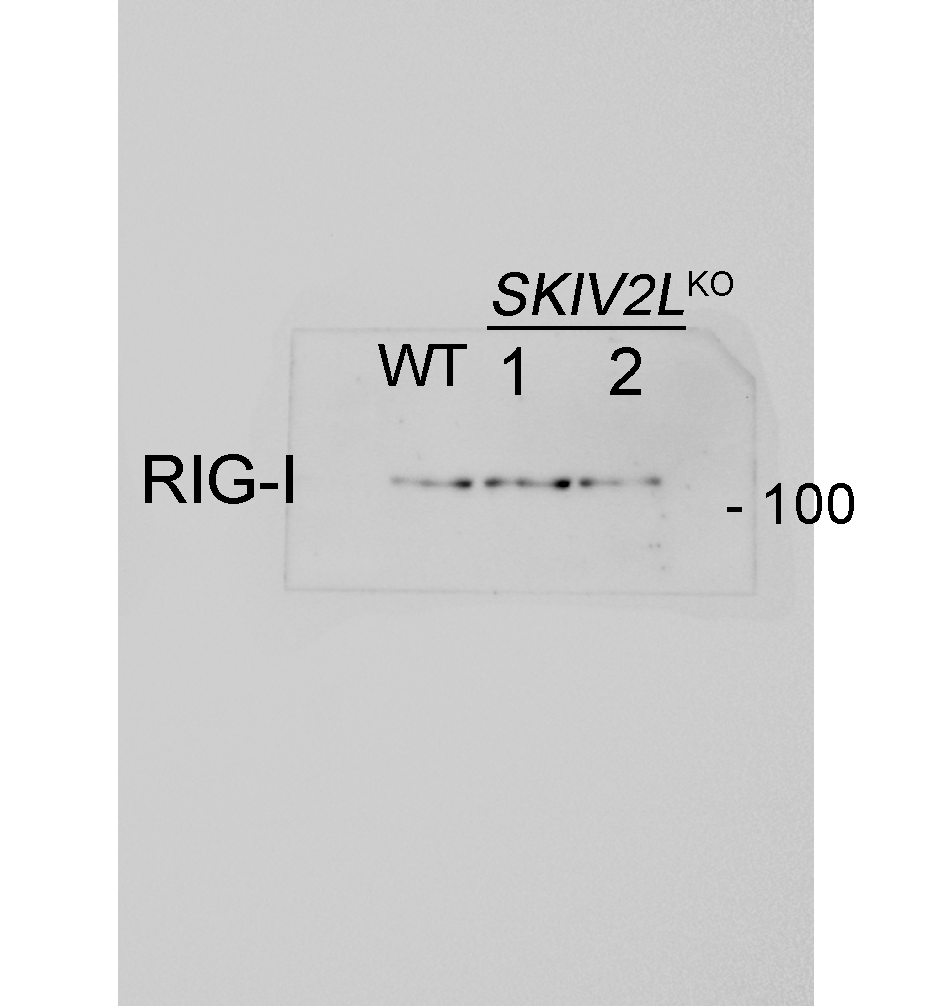

Supplement: Supplementary file 8 — EV Figure Source Data [file 44318_2024_187_MOESM8_ESM.zip › EV Figure1D/RIG-I copy.tif]

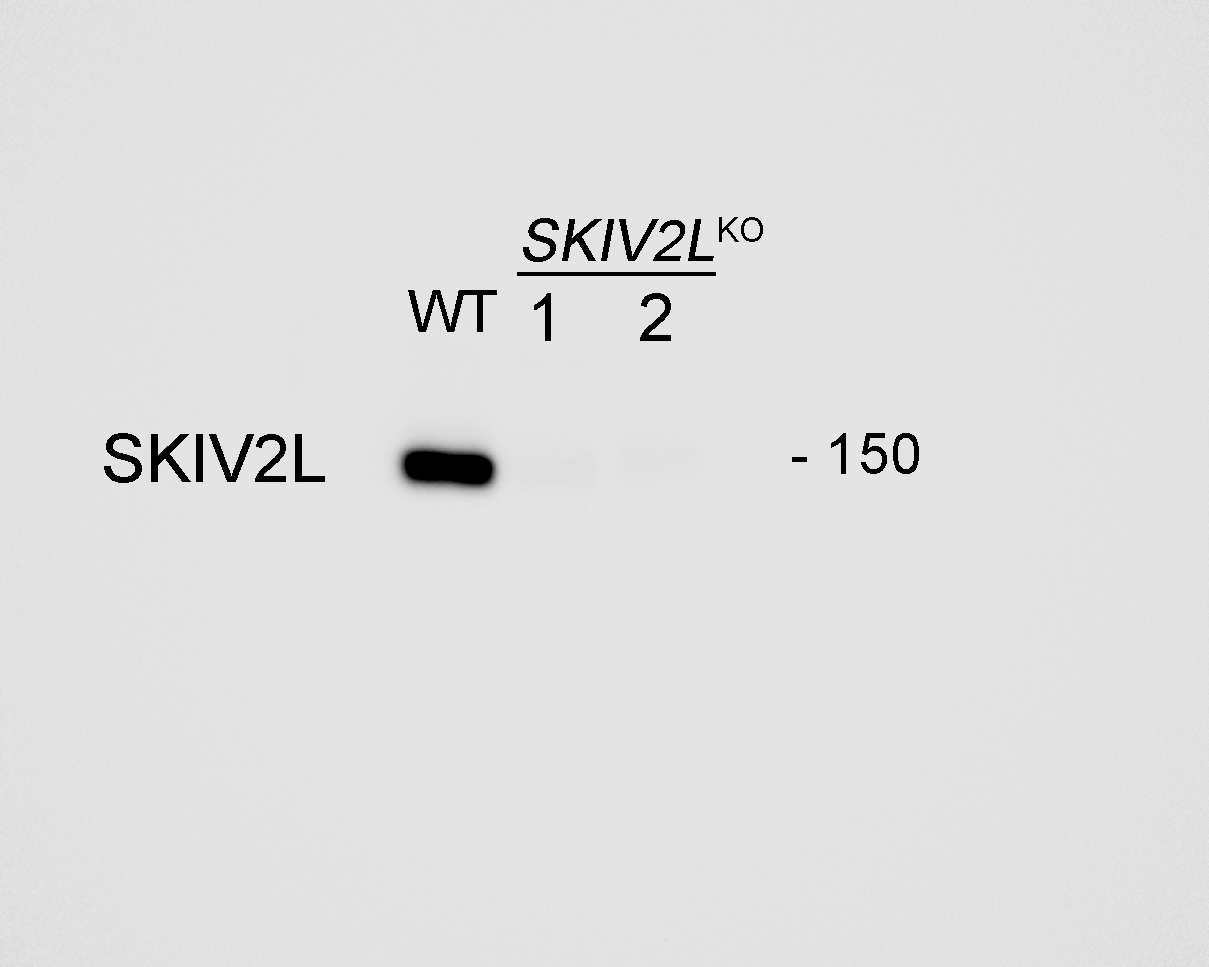

Supplement: Supplementary file 8 — EV Figure Source Data [file 44318_2024_187_MOESM8_ESM.zip › EV Figure1D/SKIV2L copy.tif]

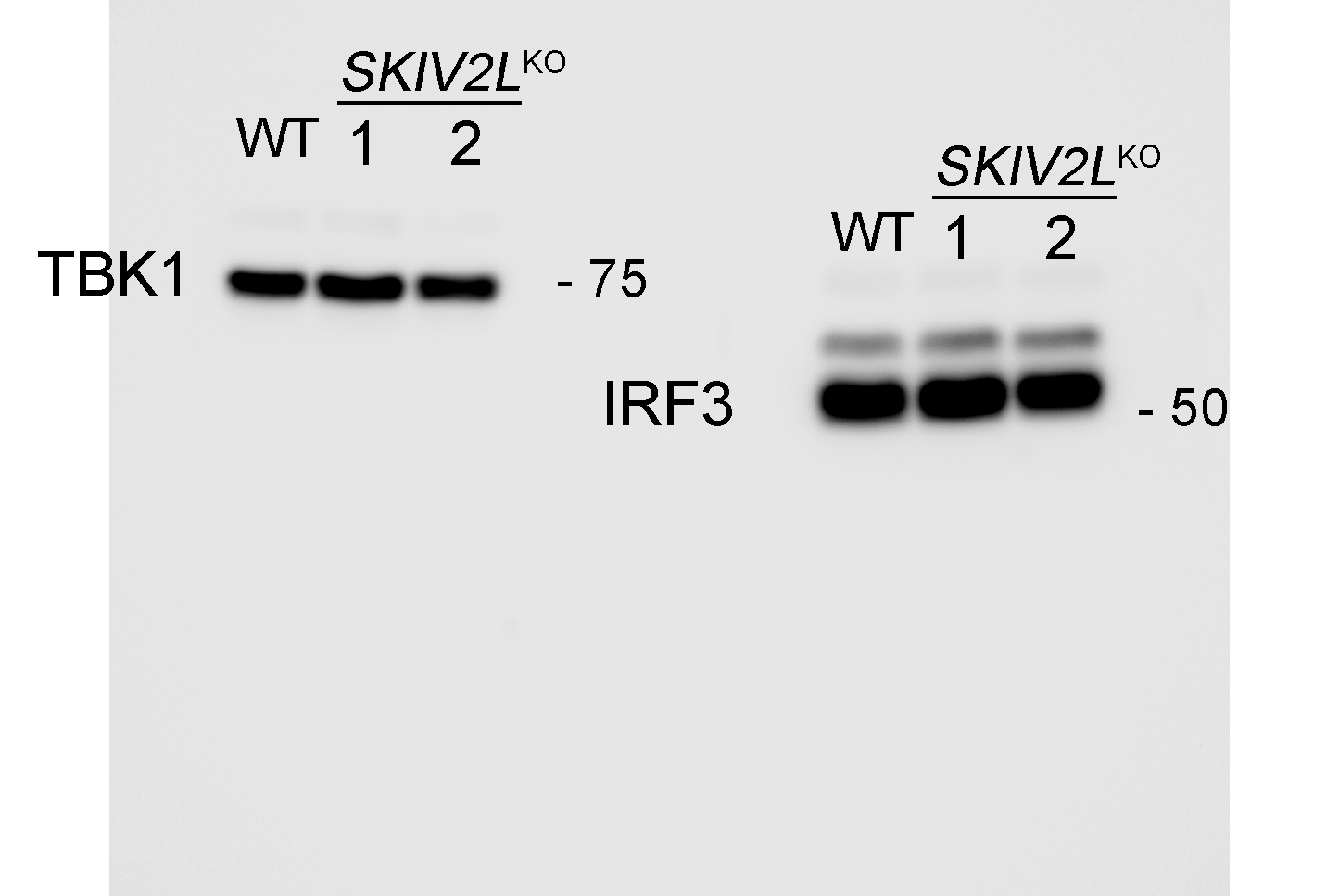

Supplement: Supplementary file 8 — EV Figure Source Data [file 44318_2024_187_MOESM8_ESM.zip › EV Figure1D/TBK1 IRF3 copy.tif]

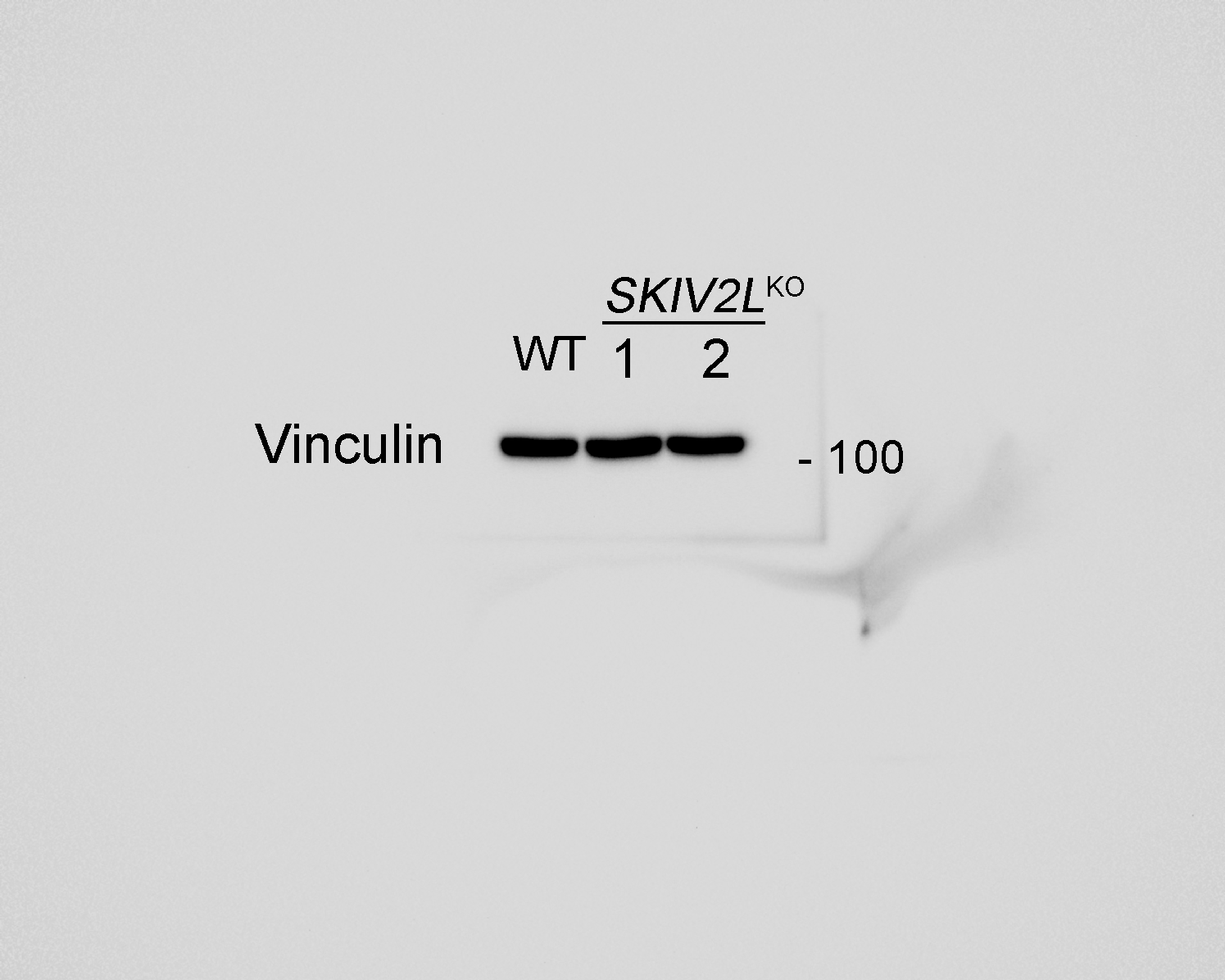

Supplement: Supplementary file 8 — EV Figure Source Data [file 44318_2024_187_MOESM8_ESM.zip › EV Figure1D/vinculin copy.tif]

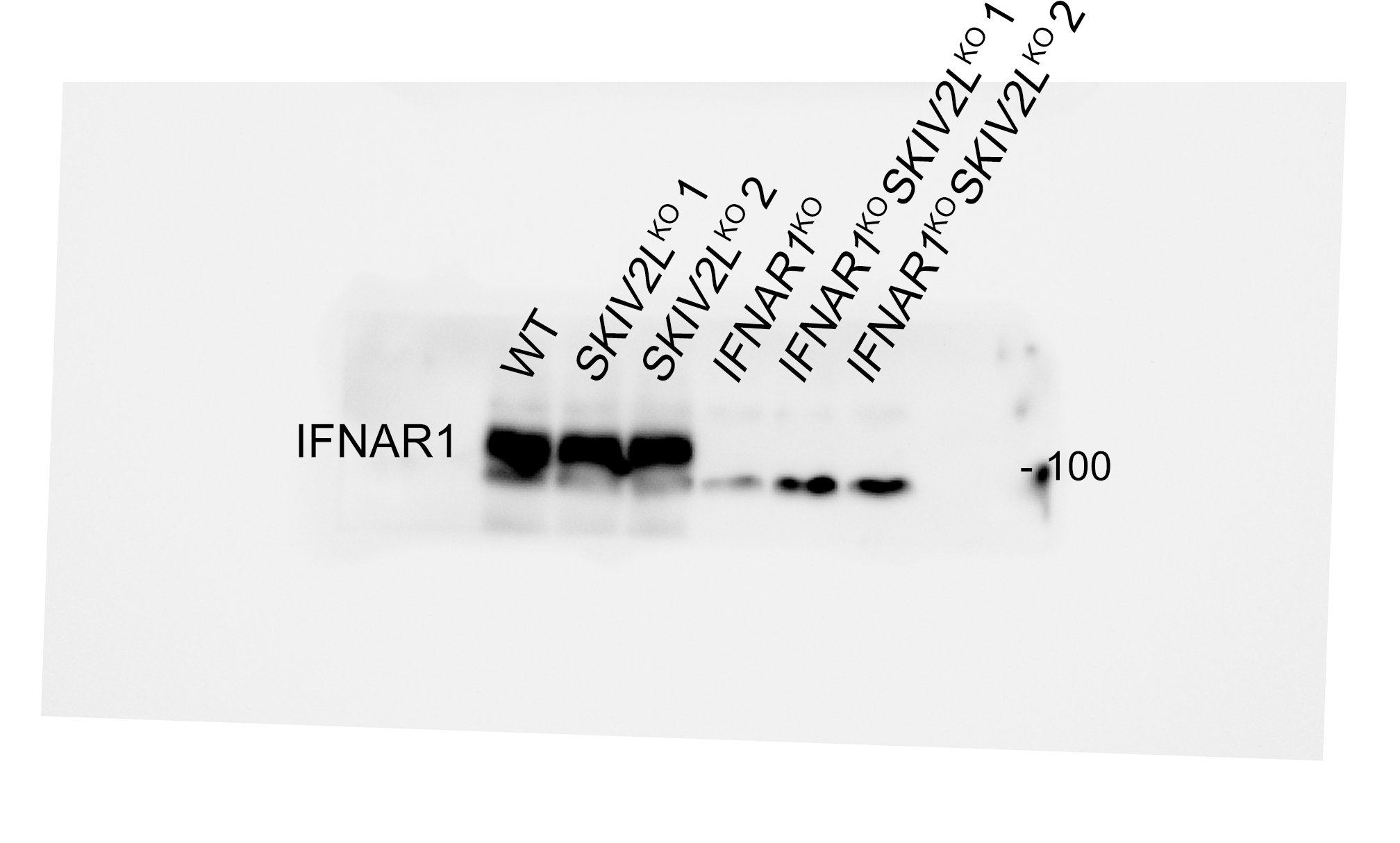

Supplement: Supplementary file 8 — EV Figure Source Data [file 44318_2024_187_MOESM8_ESM.zip › EV Figure1G/IFNAR1.tif]

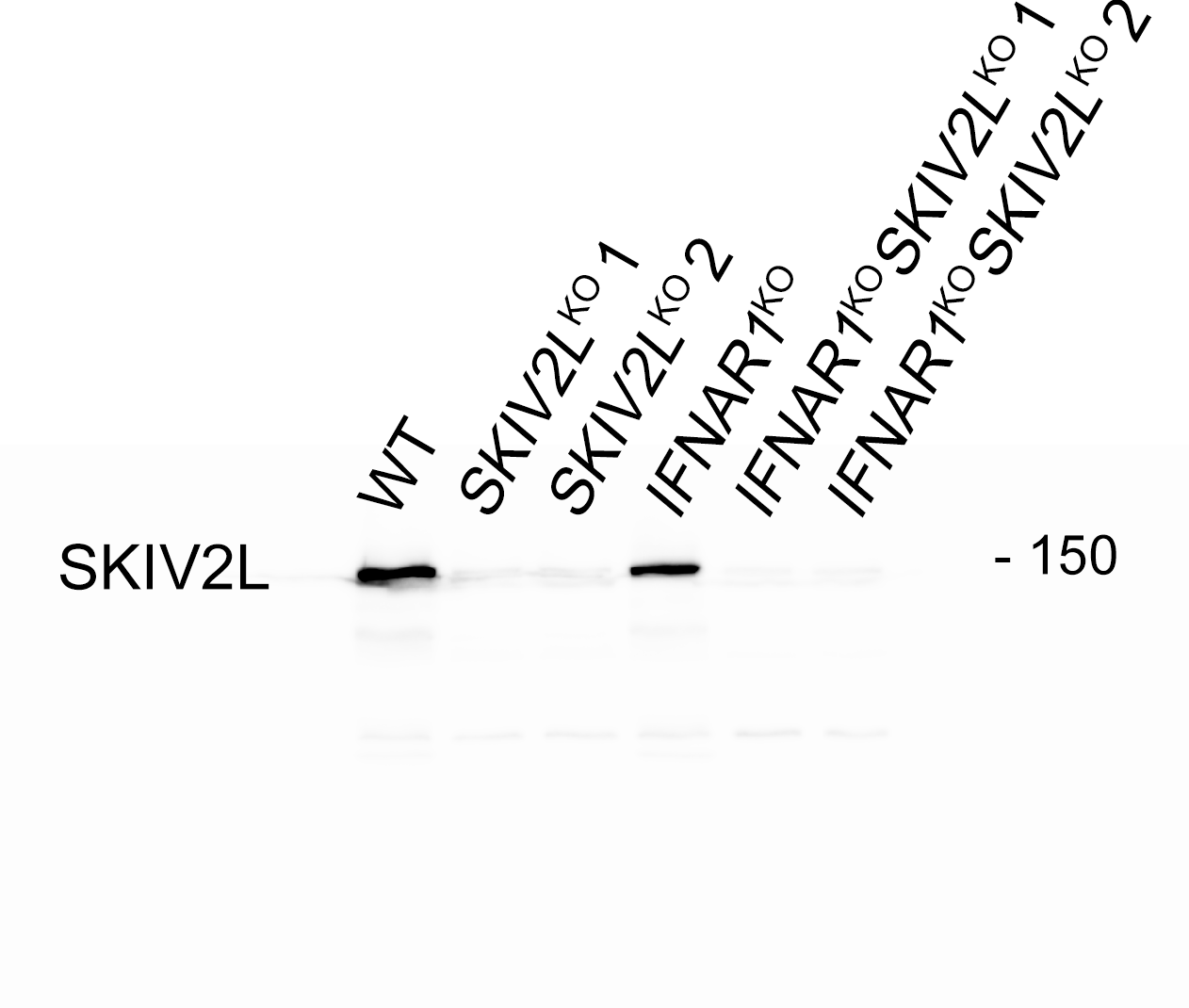

Supplement: Supplementary file 8 — EV Figure Source Data [file 44318_2024_187_MOESM8_ESM.zip › EV Figure1G/SKIV2L.tif]

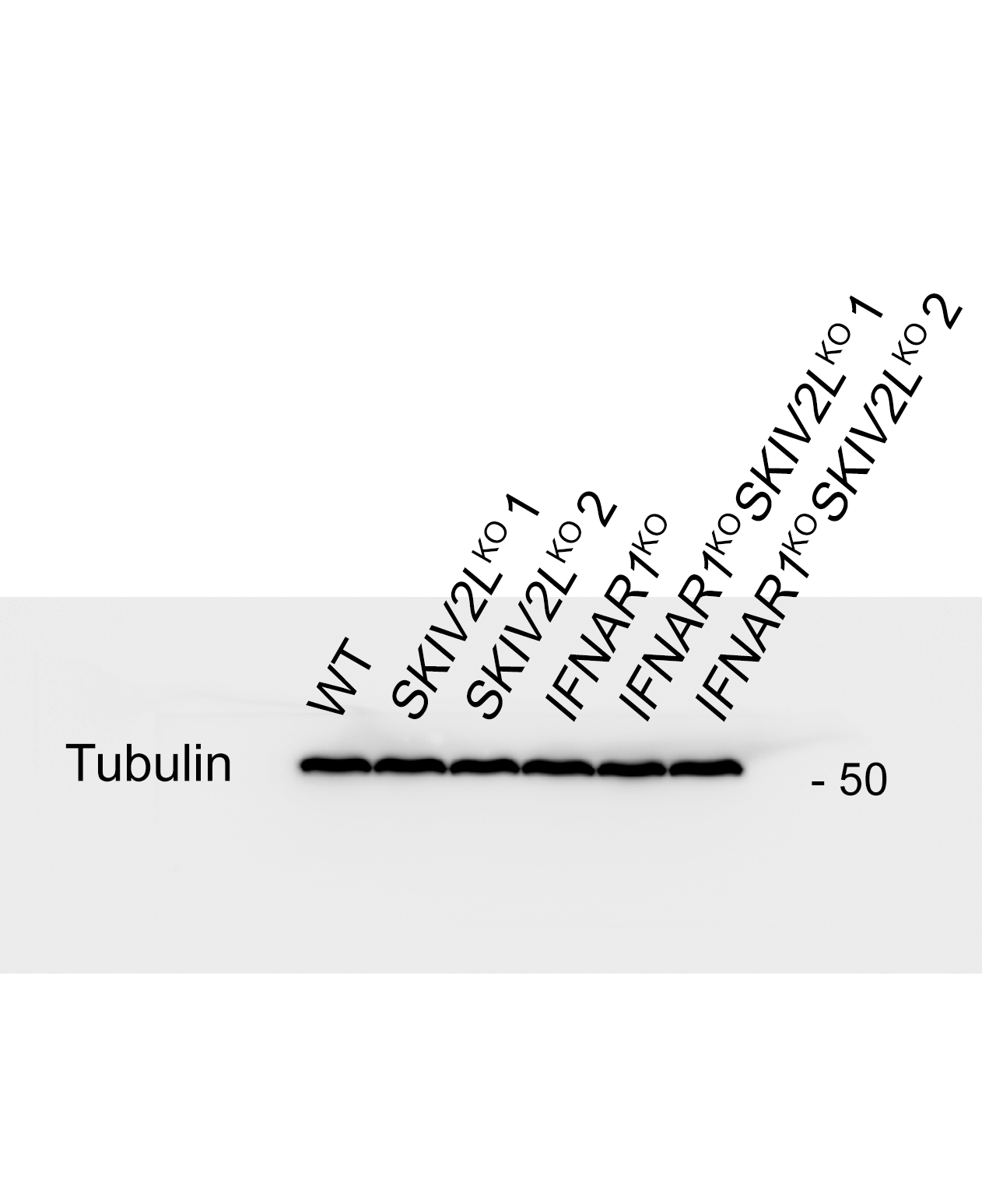

Supplement: Supplementary file 8 — EV Figure Source Data [file 44318_2024_187_MOESM8_ESM.zip › EV Figure1G/Tubulin.tif]

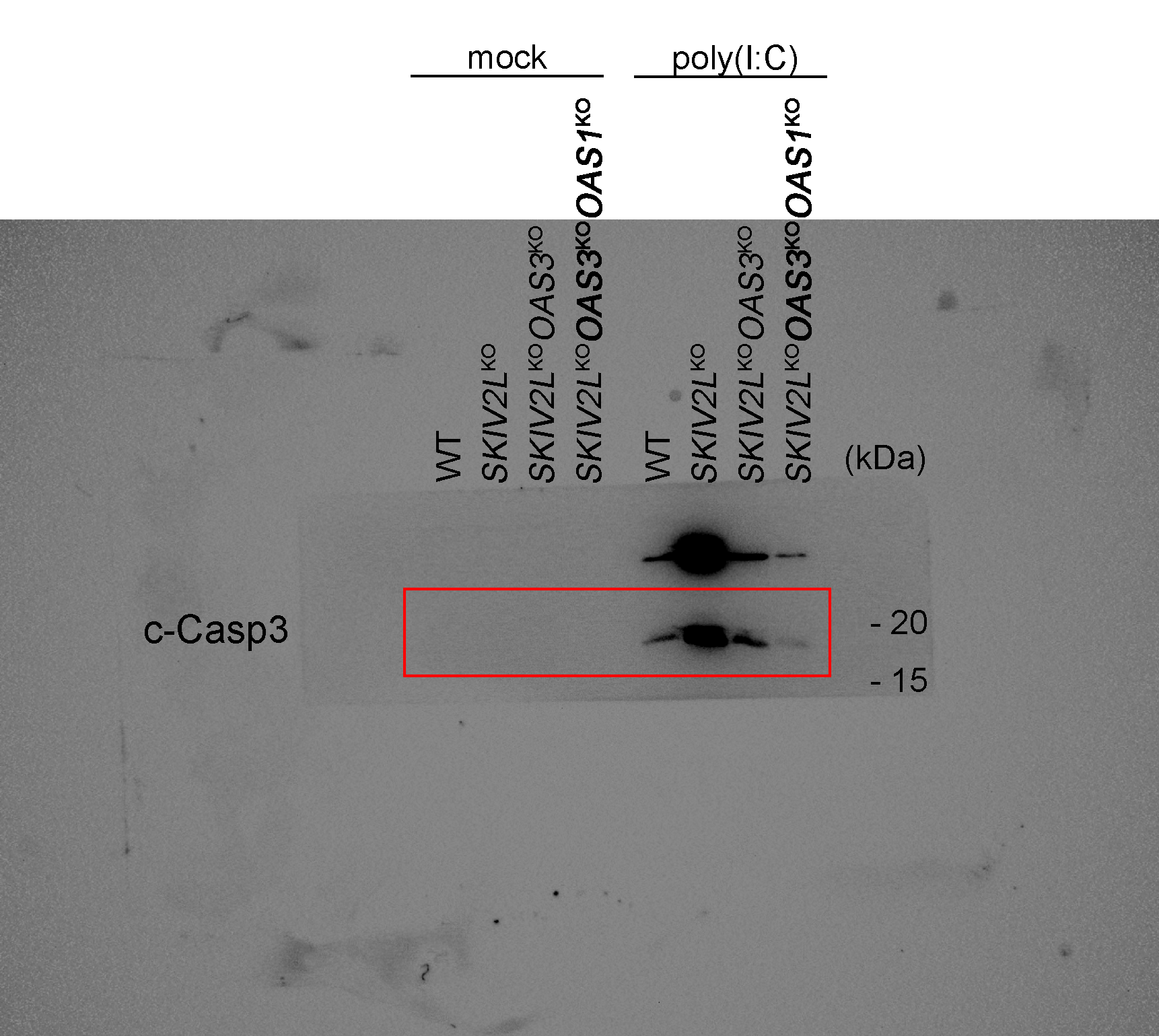

Supplement: Supplementary file 8 — EV Figure Source Data [file 44318_2024_187_MOESM8_ESM.zip › EV Figure2A/c-Casp3.tif]

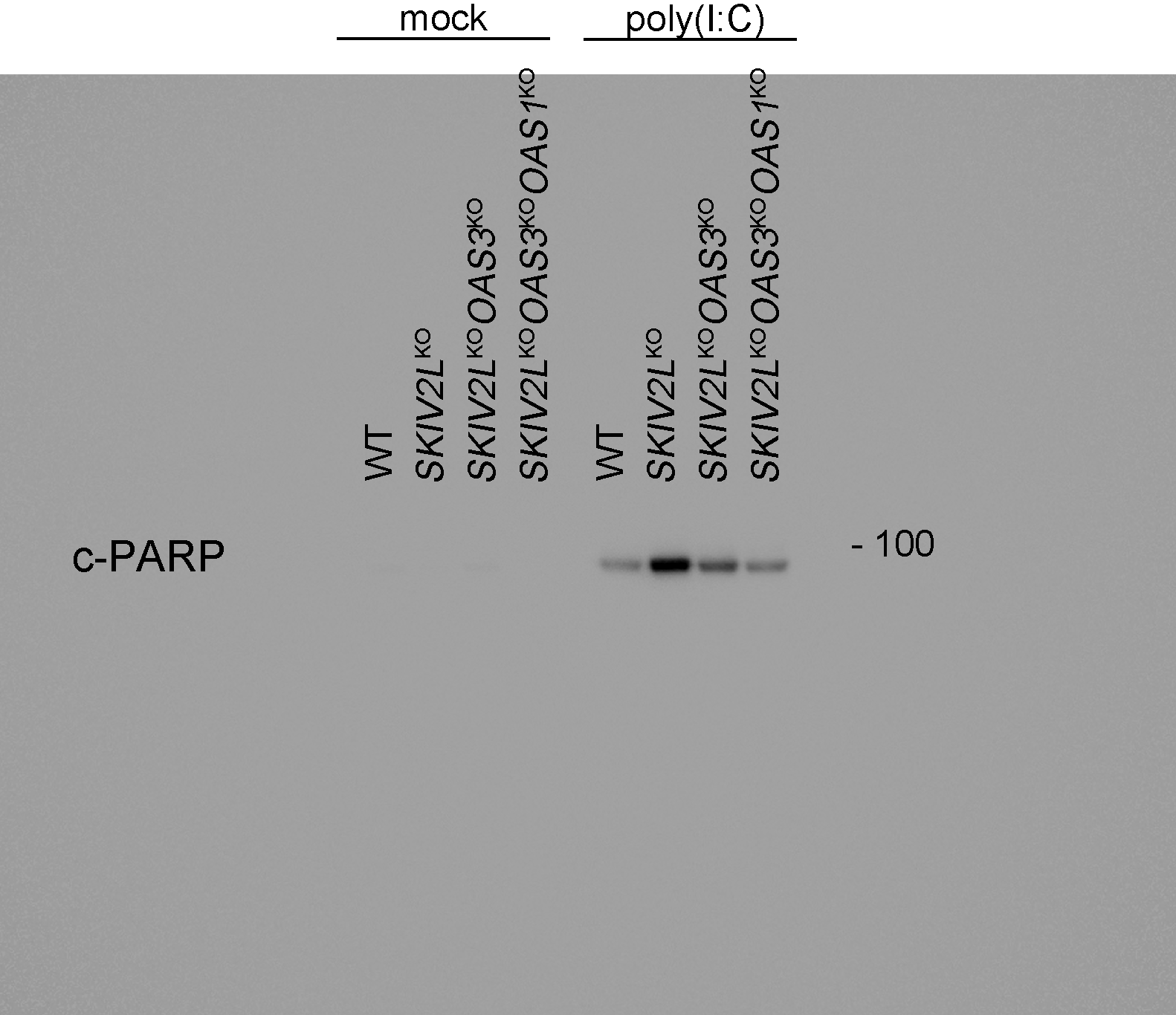

Supplement: Supplementary file 8 — EV Figure Source Data [file 44318_2024_187_MOESM8_ESM.zip › EV Figure2A/c-PARP.tif]

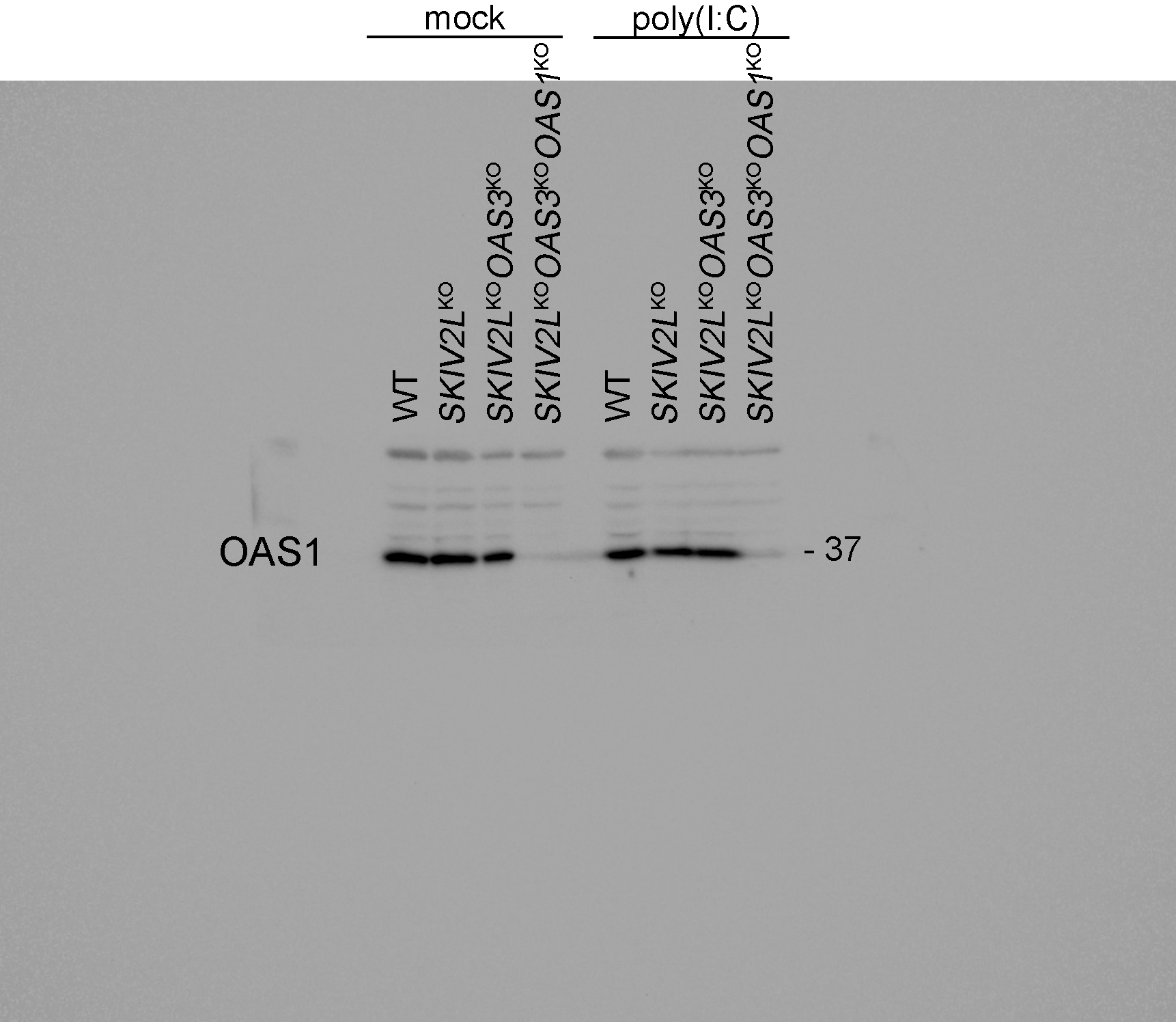

Supplement: Supplementary file 8 — EV Figure Source Data [file 44318_2024_187_MOESM8_ESM.zip › EV Figure2A/OAS1.tif]

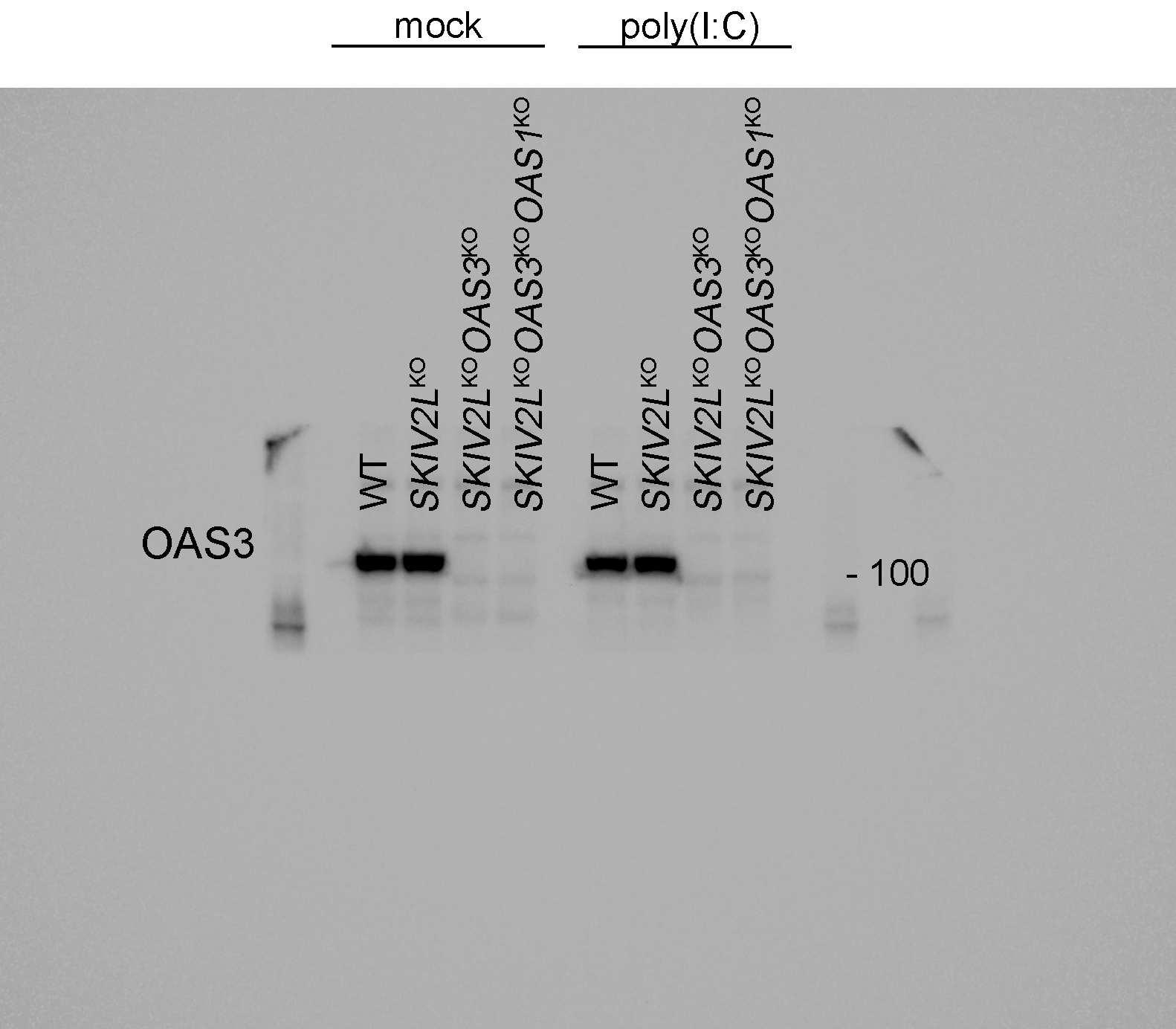

Supplement: Supplementary file 8 — EV Figure Source Data [file 44318_2024_187_MOESM8_ESM.zip › EV Figure2A/OAS3.tif]

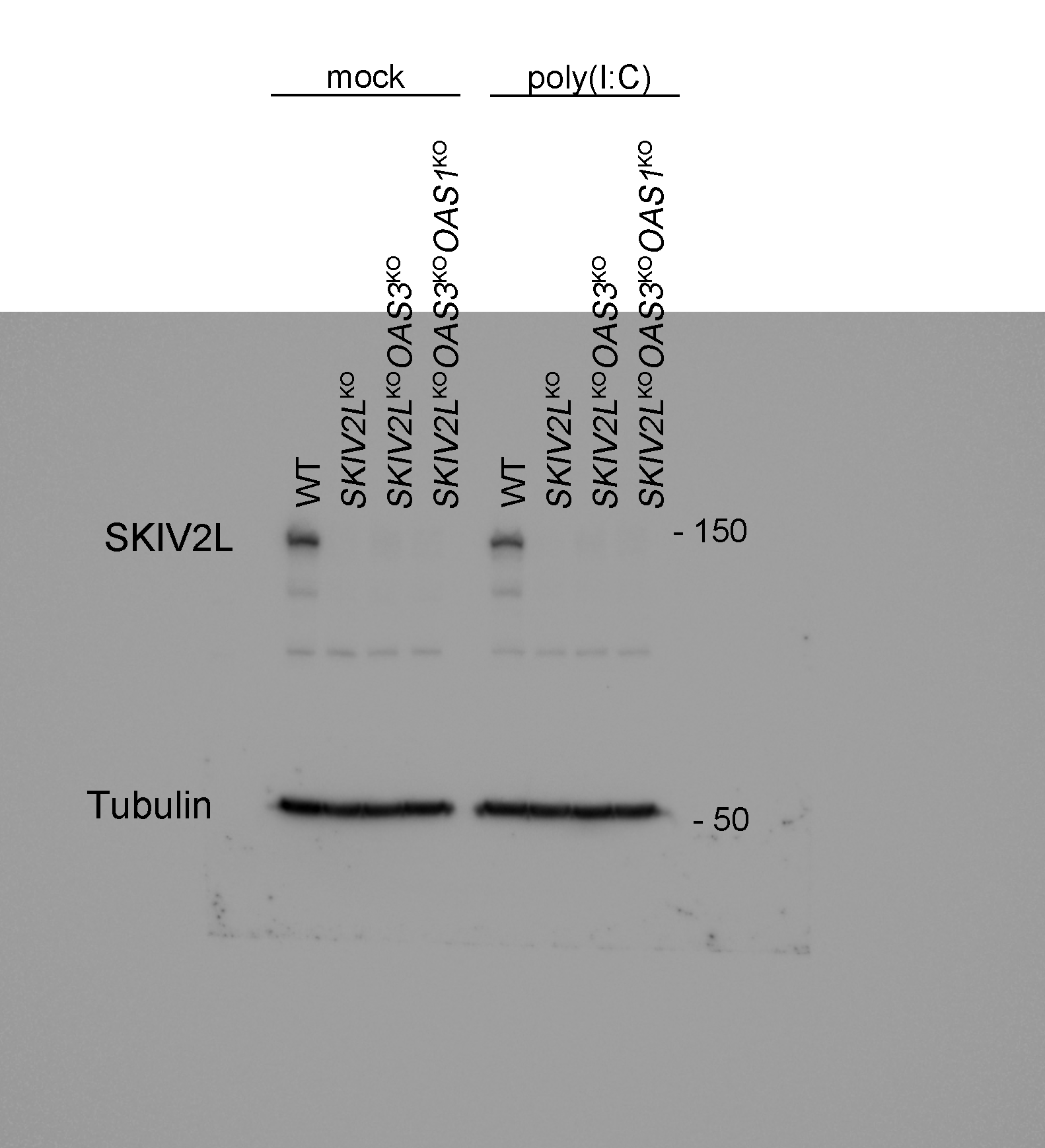

Supplement: Supplementary file 8 — EV Figure Source Data [file 44318_2024_187_MOESM8_ESM.zip › EV Figure2A/SKIV2L Tubulin.tif]

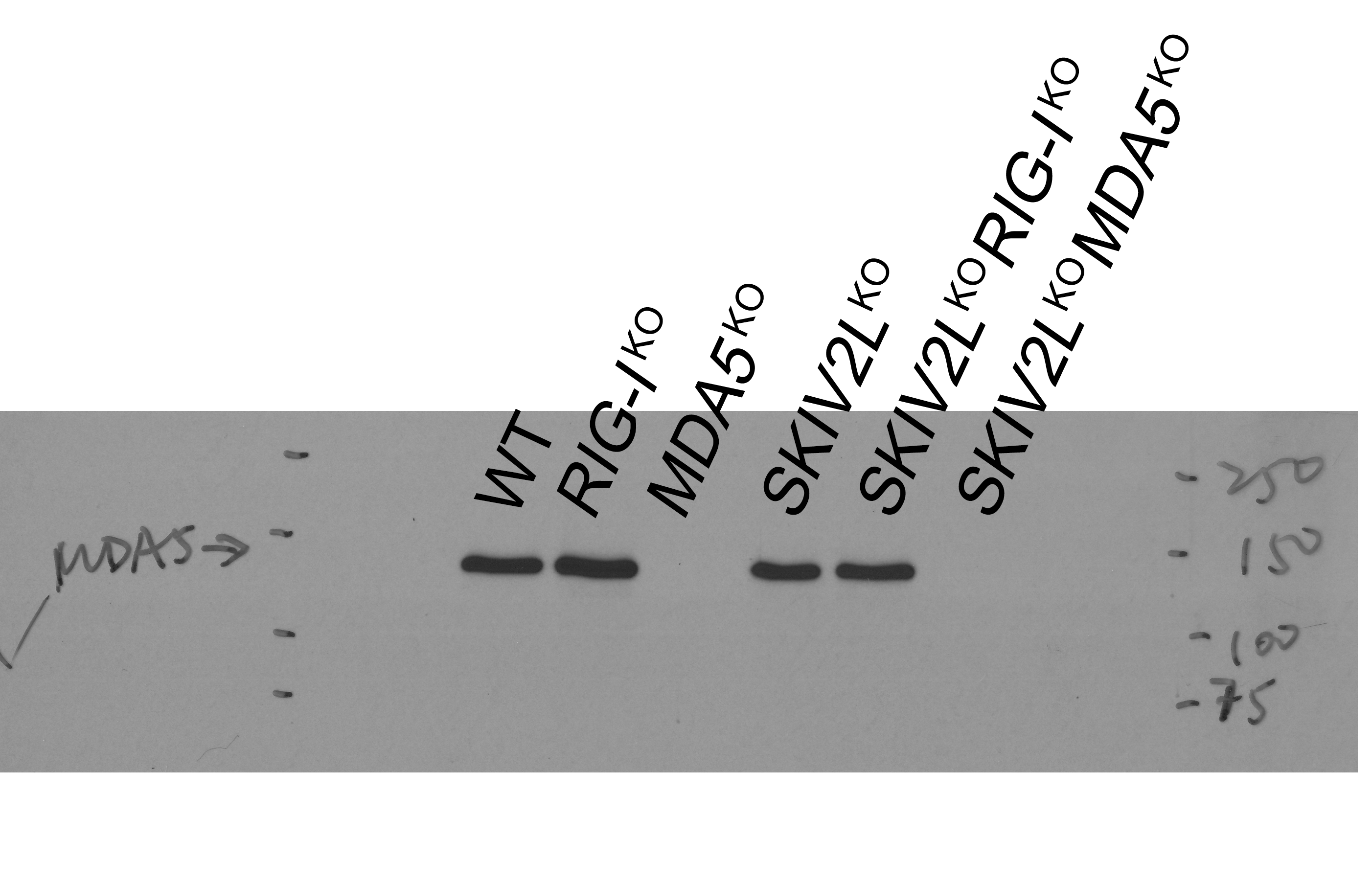

Supplement: Supplementary file 8 — EV Figure Source Data [file 44318_2024_187_MOESM8_ESM.zip › EV Figure2B/MDA5.tif]
