# Supplementary figures and images for: RNA helicase SKIV2L limits antiviral defense and autoinflammation elicited by the OAS-RNase L pathway (part 2 of 2)
Source: EMBO J. 2024 Aug 7;43(18):3876–94. doi: 10.1038/s44318-024-00187-1 (PMC11405415; doi:10.1038/s44318-024-00187-1)

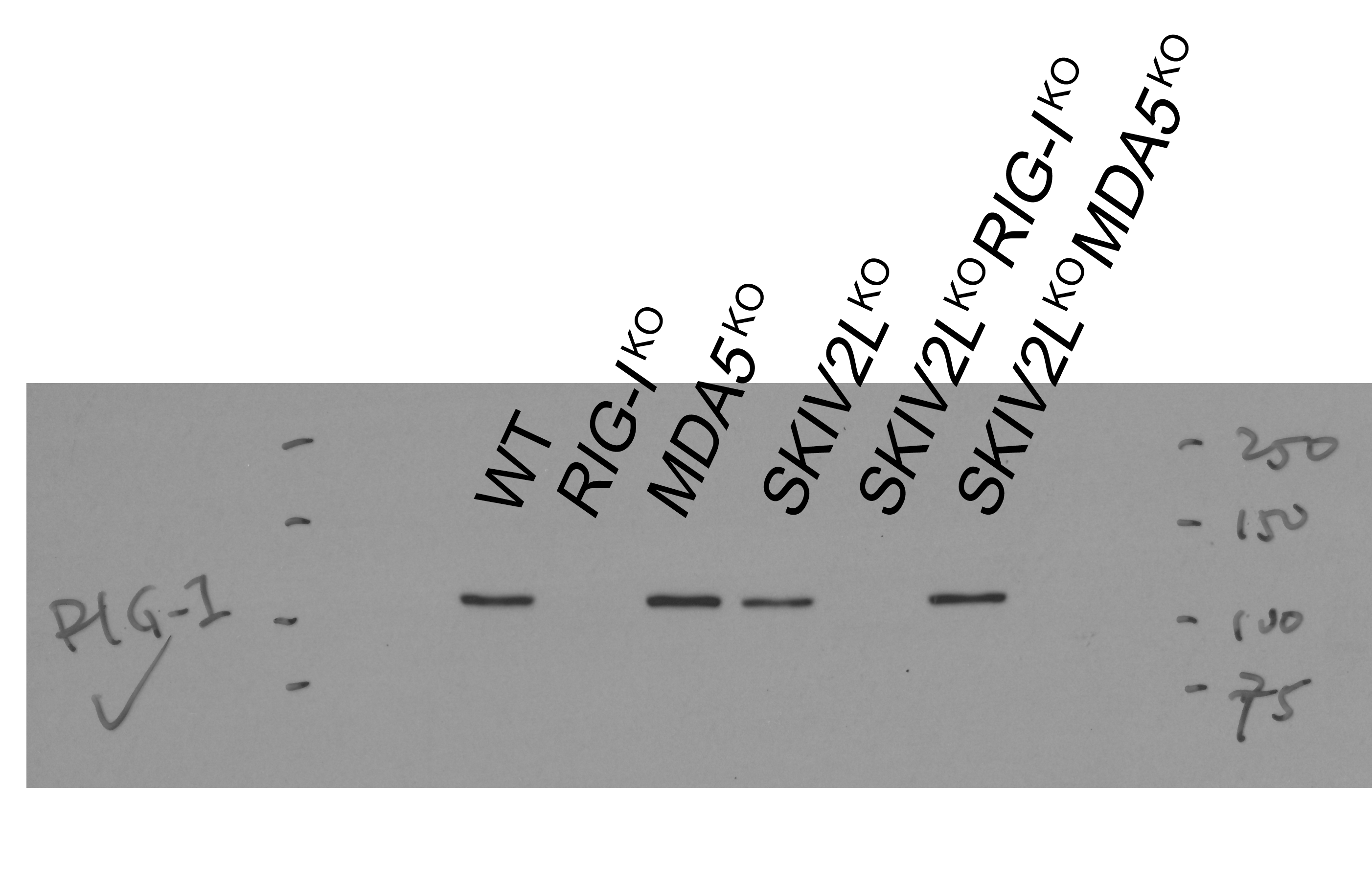

Supplement: Supplementary file 8 — EV Figure Source Data [file 44318_2024_187_MOESM8_ESM.zip › EV Figure2B/RIG-I.tif]

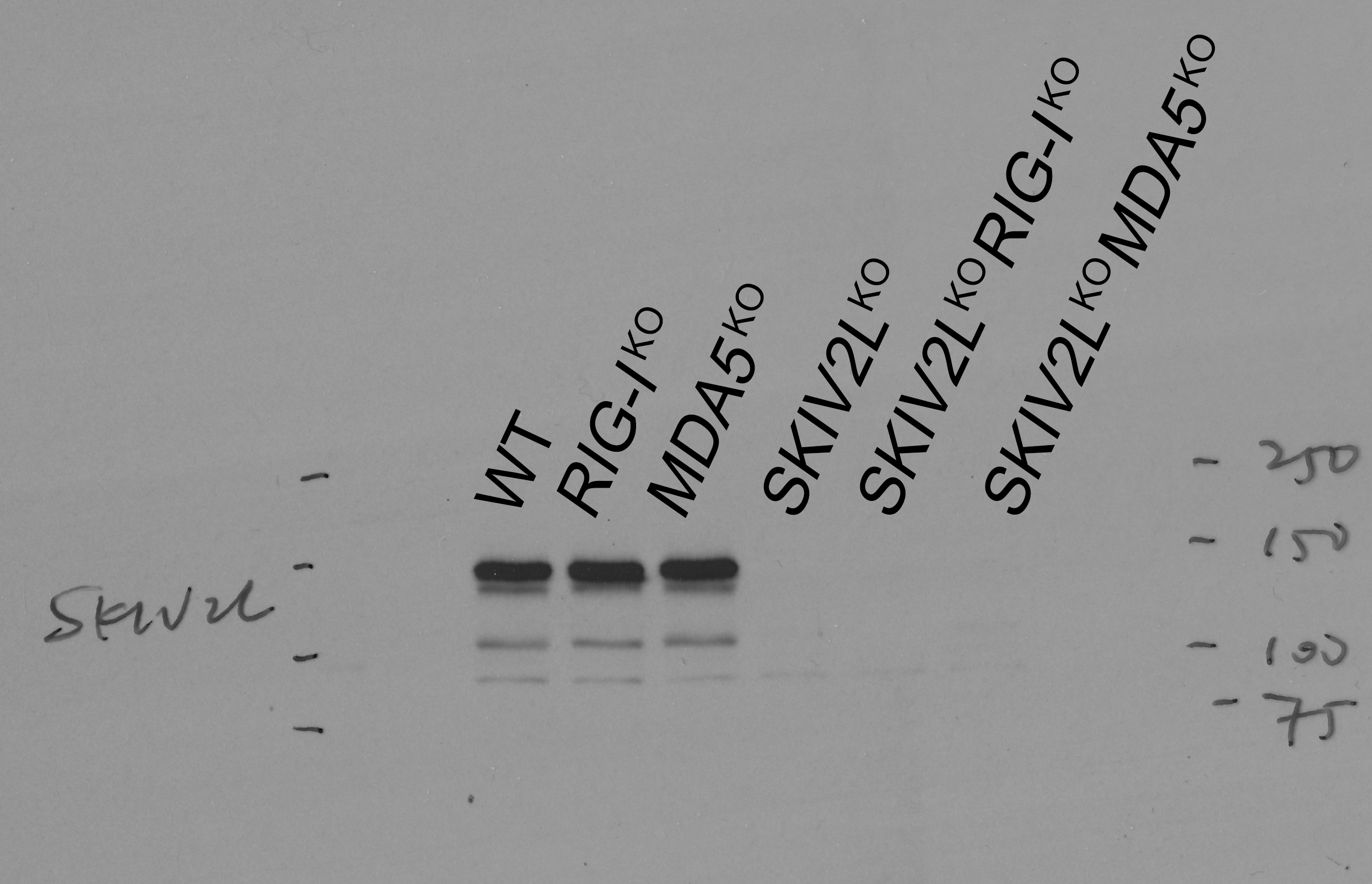

Supplement: Supplementary file 8 — EV Figure Source Data [file 44318_2024_187_MOESM8_ESM.zip › EV Figure2B/SKIV2L.tif]

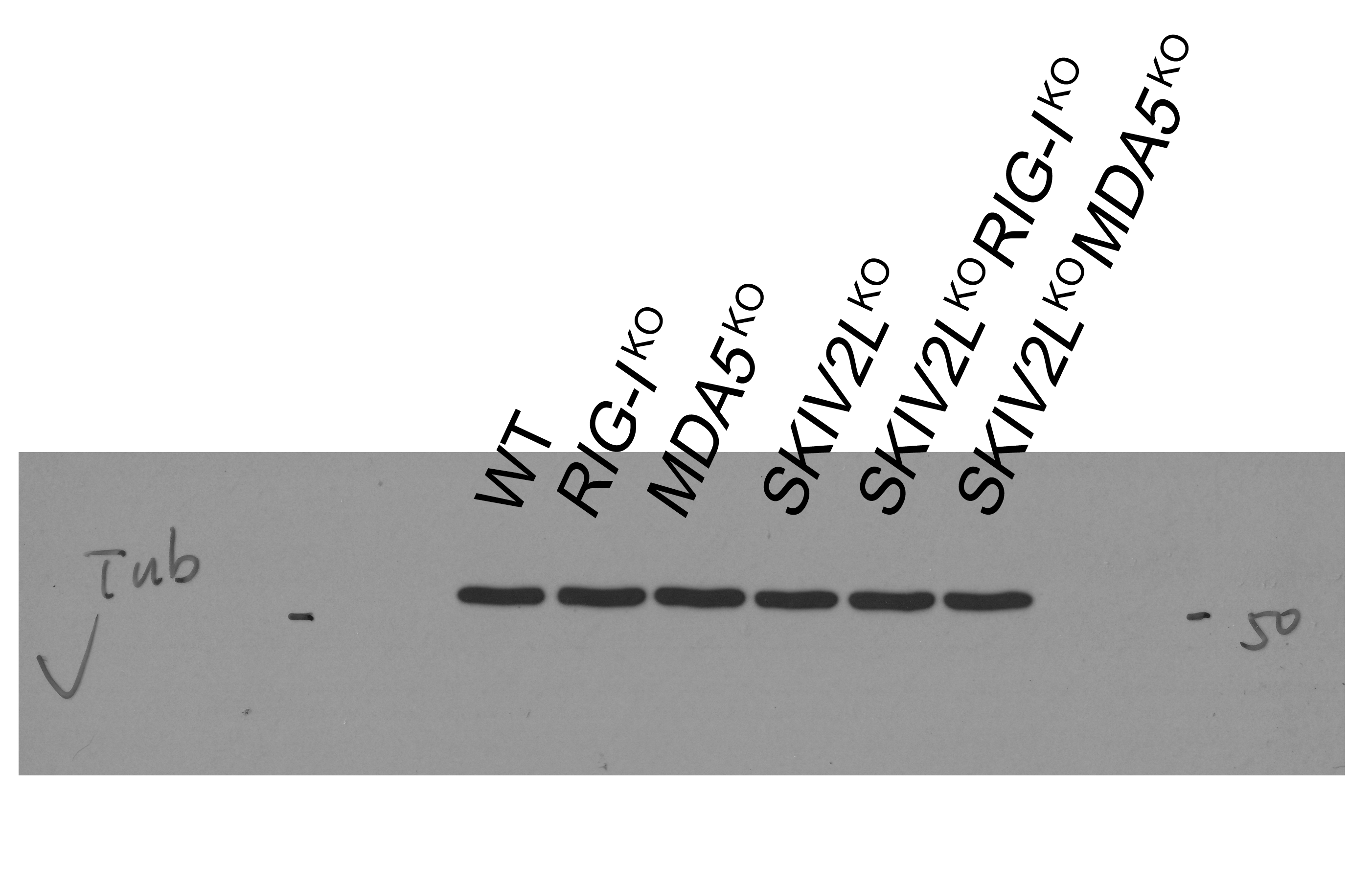

Supplement: Supplementary file 8 — EV Figure Source Data [file 44318_2024_187_MOESM8_ESM.zip › EV Figure2B/Tubulin.tif]

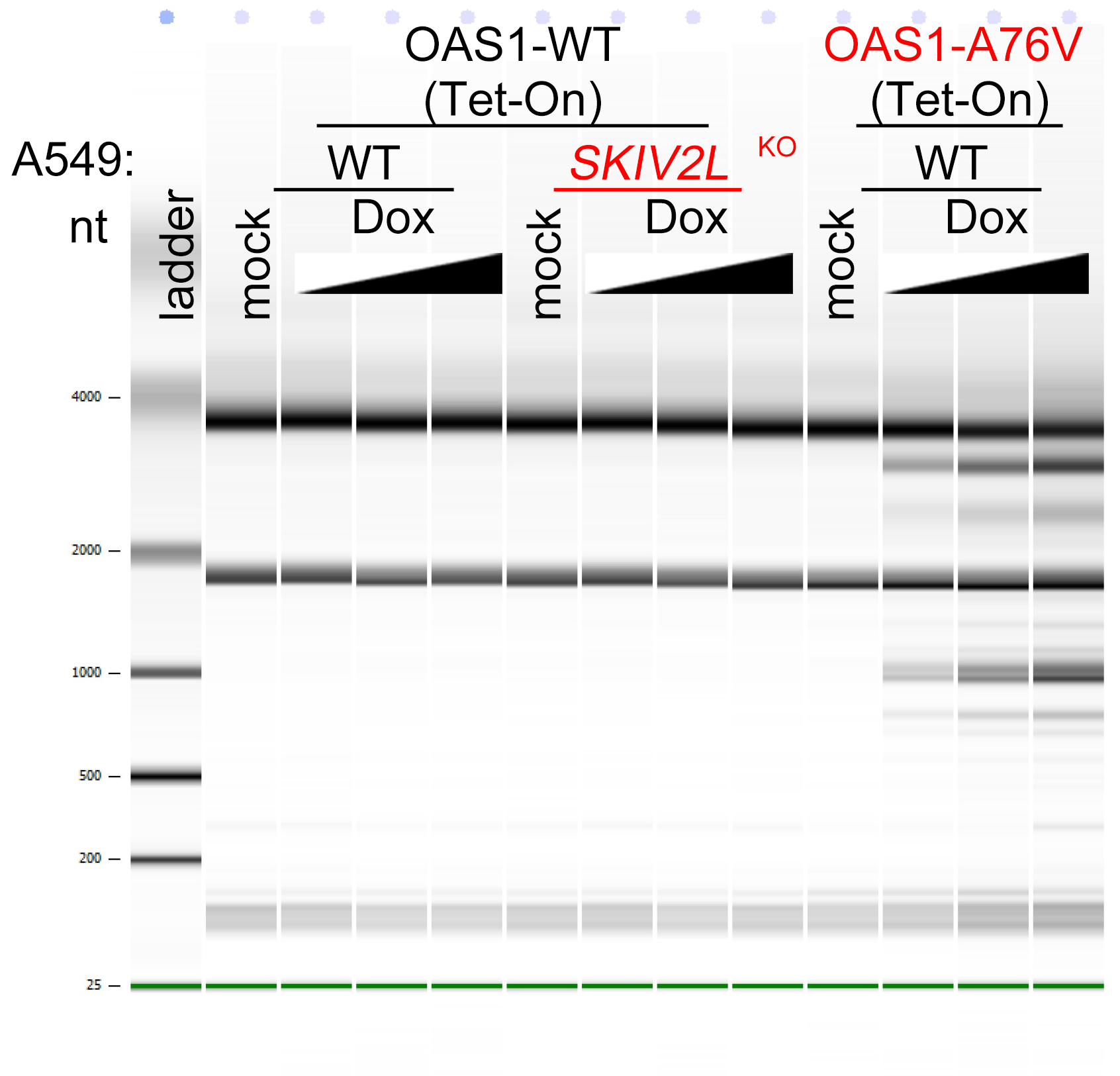

Supplement: Supplementary file 8 — EV Figure Source Data [file 44318_2024_187_MOESM8_ESM.zip › EV Figure3A/Bioanalyzer.tif]

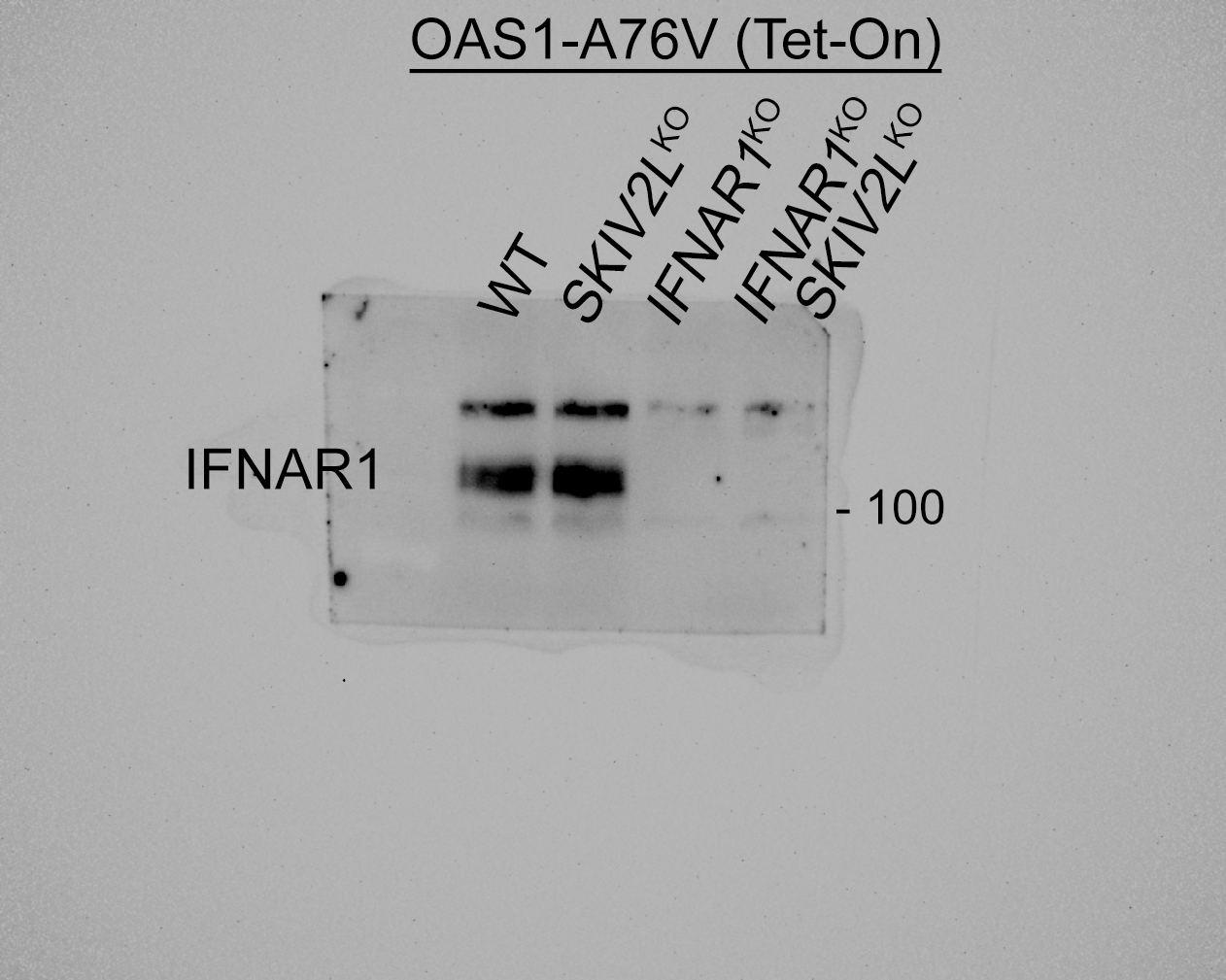

Supplement: Supplementary file 8 — EV Figure Source Data [file 44318_2024_187_MOESM8_ESM.zip › EV Figure3C/IFNAR1.tif]

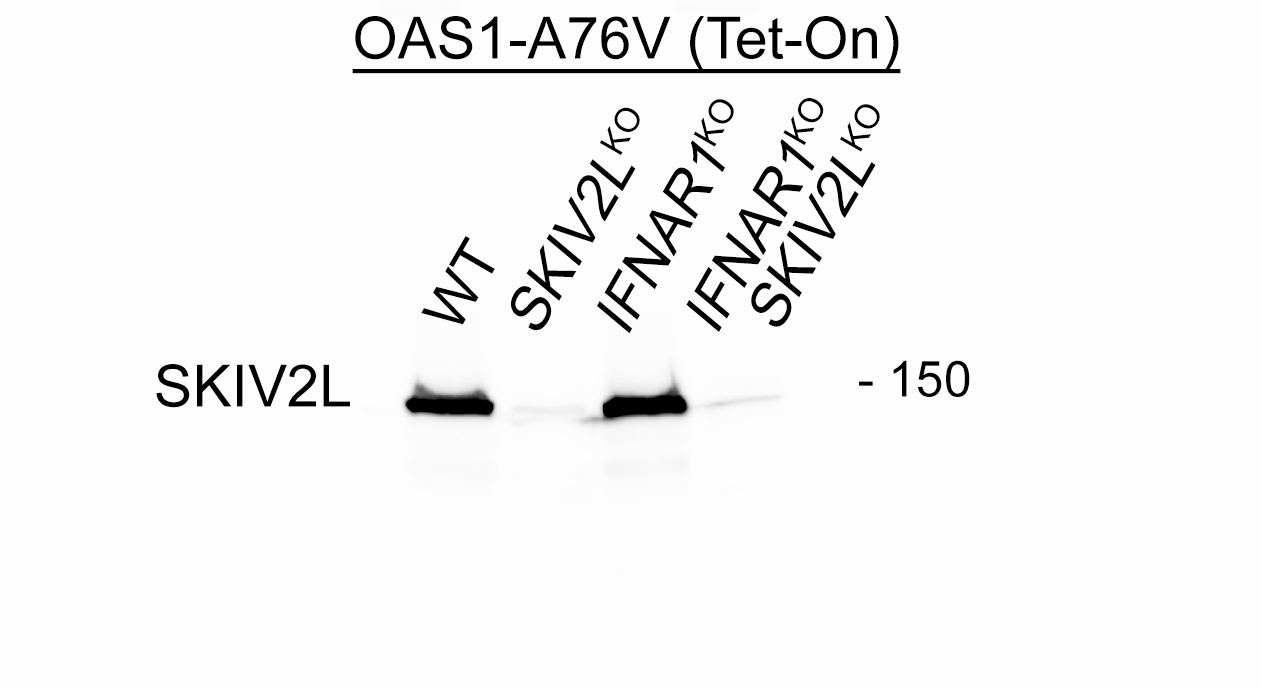

Supplement: Supplementary file 8 — EV Figure Source Data [file 44318_2024_187_MOESM8_ESM.zip › EV Figure3C/SKIV2L.tif]

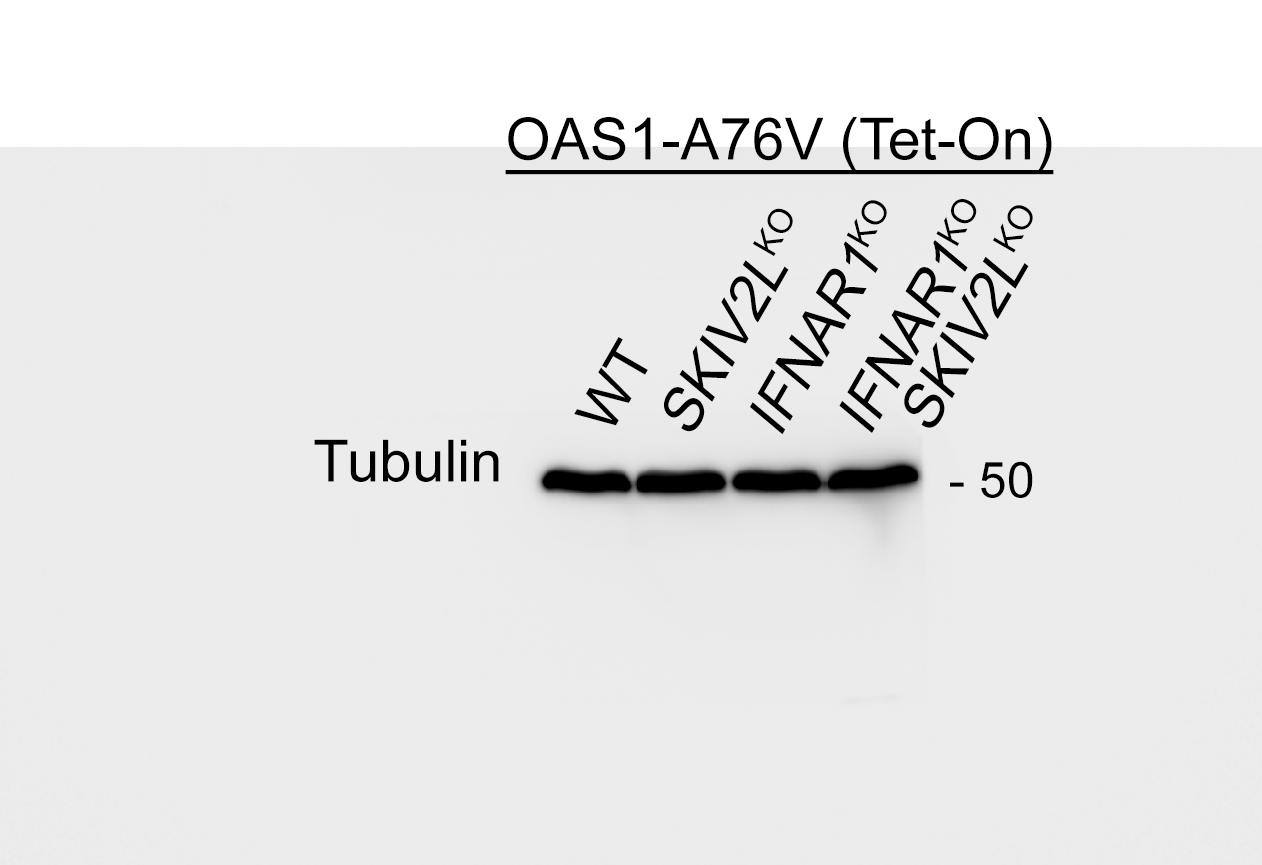

Supplement: Supplementary file 8 — EV Figure Source Data [file 44318_2024_187_MOESM8_ESM.zip › EV Figure3C/Tubulin.tif]
