# Supplementary material for: RNA helicase SKIV2L limits antiviral defense and autoinflammation elicited by the OAS-RNase L pathway
Source: EMBO J. 2024 Aug 7;43(18):3876–94. doi: 10.1038/s44318-024-00187-1 (PMC11405415; doi:10.1038/s44318-024-00187-1)
Supplement: Supplementary file 9 — Expanded View Figures [file 44318_2024_187_MOESM9_ESM.pdf]

Expanded View Figures

Figure EV1. *SKIV2L* deficiency enhances dsRNA-induced type I IFN response.

(**A**) RT-qPCR analysis of *IFNB1* mRNA in WT and two independent lines of $SKIV2L^{KO}$ cells treated with high molecular weight (HMW) or low molecular weight (LMW) poly(I:C) (1.0 μg/ml) for 4 h. Data are shown as mean ± SEM of three independent experiments. Two-sided Student's *t* test; $^{***}P < 0.001$. *P* values = 0.000008 (HMW, WT vs $SKIV2L^{KO}$ 1), 0.000123 (LMW, WT vs $SKIV2L^{KO}$ 1), 0.000965 (HMW, WT vs $SKIV2L^{KO}$ 2), 0.000293 (LMW, WT vs $SKIV2L^{KO}$ 2). (**B**) RT-qPCR analysis of *IFNB1* mRNA in WT and $SKIV2L^{KO}$ cells treated with 3p-hpRNA at indicated concentration for 4 h. Data are shown as mean ± SEM of four independent experiments. Two-sided Student's *t* test; $^{**}P < 0.01$, $^{***}P < 0.001$. *P* values = 0.000007 (150 ng/ml), 0.000038 (500 ng/ml). (**C**) Western blot analysis of RSAD2 proteins in WT and two independent lines of $SKIV2L^{KO}$ A549 cells after poly(I:C) (0.1 μg/ml) treatment for 8 h. (**D**) Western blot analysis of proteins of RNA sensing pathway in WT and two independent lines of $SKIV2L^{KO}$ A549 cells. (**E**) RT-qPCR analysis of genes of RNA sensing pathway in WT and two independent lines of $SKIV2L^{KO}$ A549 cells. Data are shown as mean ± SEM of three independent experiments. (**F**) RT-qPCR analysis of ISG expression in WT and two independent lines of $SKIV2L^{KO}$ A549 cells. Data are shown as mean ± SEM of three independent experiments. (**G**) Western blot analysis of IFNAR1 and SKIV2L proteins in WT and indicated knockout cells. (**H**) RT-qPCR analysis of *IFNB1* mRNA in WT and indicated knockout cells after poly(I:C) (1.0 μg/ml) treatment for 4 h or SINV infection (MOI 0.3) for 24 h. Data are shown as mean ± SEM of four independent experiments. Two-sided Student's *t* test; $^{***}P < 0.001$. *P* values = 0.000003 (poly(I:C), $IFNAR1^{KO}$ vs $IFNAR1^{KO}SKIV2L^{KO}$ 1), 0.000283 (poly(I:C), $IFNAR1^{KO}$ vs $IFNAR1^{KO}SKIV2L^{KO}$ 2), 0.000366 (SINV, $IFNAR1^{KO}$ vs $IFNAR1^{KO}SKIV2L^{KO}$ 1), 0.000002 (SINV, $IFNAR1^{KO}$ vs $IFNAR1^{KO}SKIV2L^{KO}$ 2). (**I**) RT-qPCR analysis of *IFNB1* and ISGs mRNA in WT and two independent lines of $SKIV2L^{KO}$ A549 cells after recombinant IFN-b treatment (20 ng/ml for 24 h). Data are shown as mean ± SEM of three independent experiments. Source data are available online for this figure.

 © The Author(s)

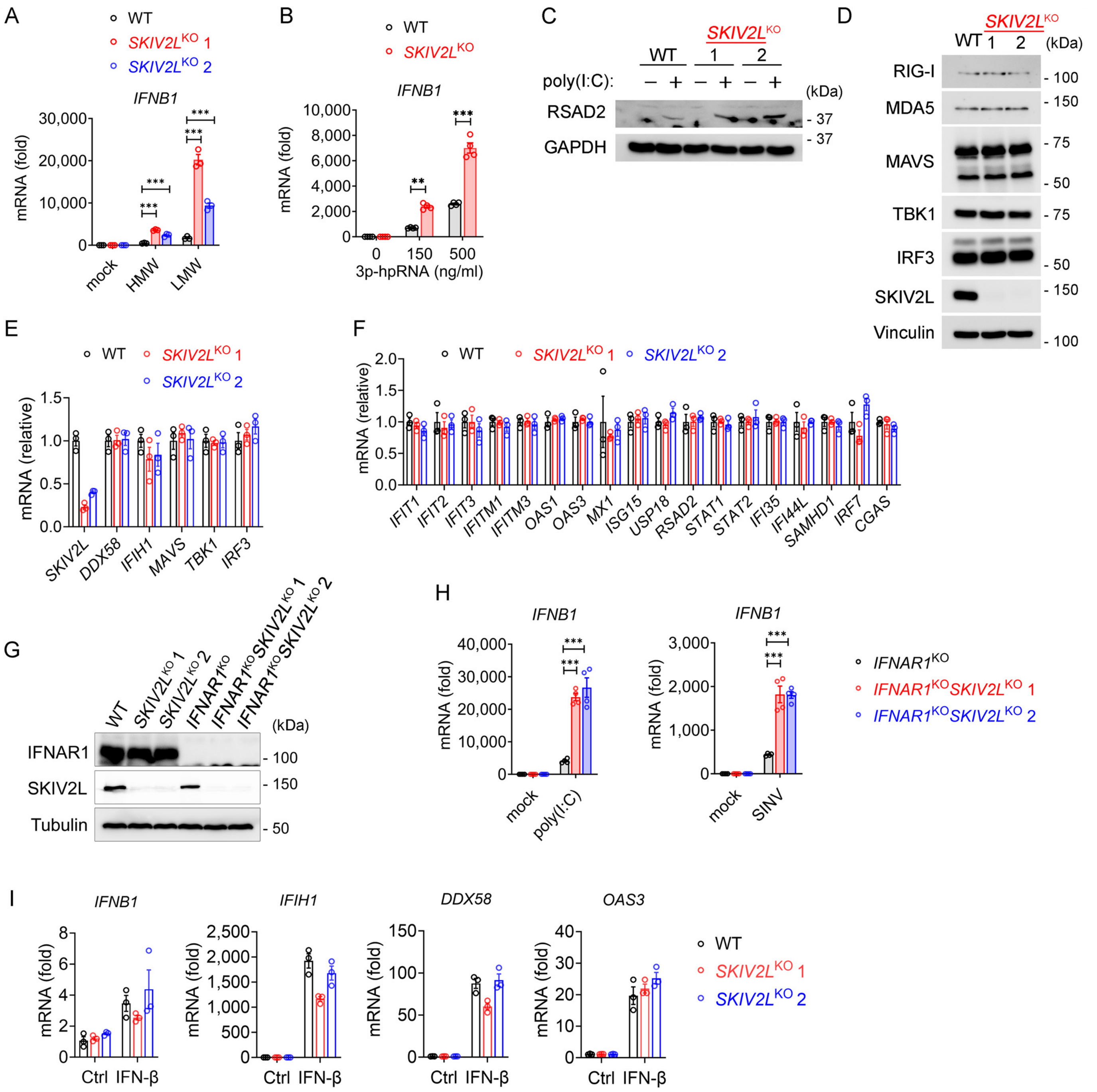
A
WT
SKIV2L KO 1
SKIV2L KO 2
IFNB1
mRNA (fold)
mock
HMW
LMW
B
3p-hpRNA (ng/ml)
C
poly(I:C):
RSAD2
GAPDH
(kDa)
D
RIG-I
MDA5
MAVS
TBK1
IRF3
SKIV2L
Vinculin
E
mRNA (relative)
SKIV2L
DDX58
IFIH1
MAVS
TBK1
IRF3
F
IFIT1
IFIT2
IFIT3
IFITM1
IFITM3
OAS1
OAS3
MX1
ISG15
USP18
RSAD2
STAT1
STAT2
IFI35
IFI44L
SAMHD1
IRF7
CGAS
G
IFNAR1
SKIV2L
Tubulin
H
IFNAR1 KO
IFNAR1 KO SKIV2L KO 1
IFNAR1 KO SKIV2L KO 2
poly(I:C)
SINV
I
IFIH1
DDX58
OAS3
Ctrl
IFN-β

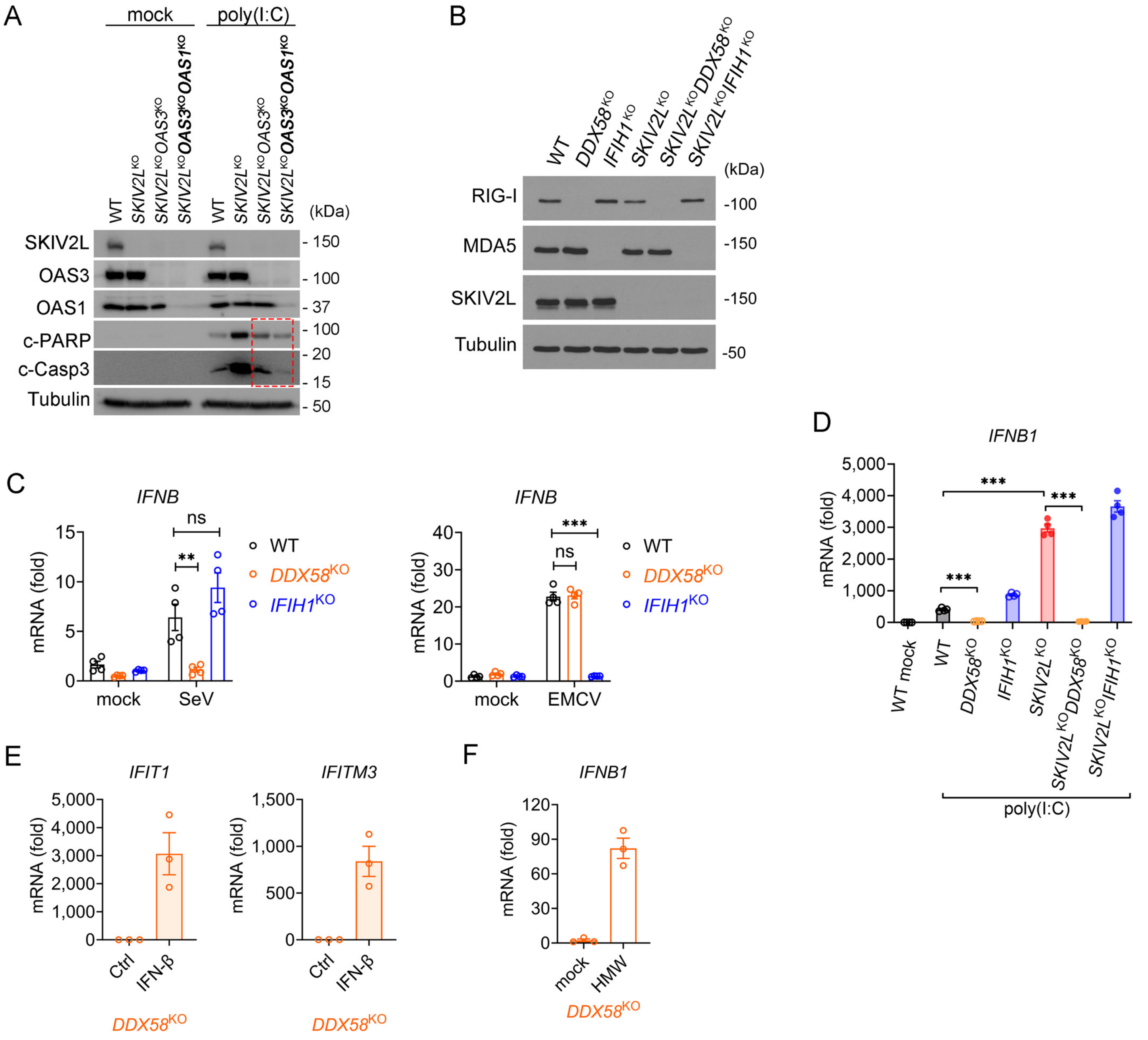

Figure EV2. SKIV2L negatively regulates the OAS-RNase L pathway.

(**A**) Western blot analysis of apoptosis in WT, *SKIV2L*^KO, *SKIV2L*^KO*OAS3*^KO, *SKIV2L*^KO*OAS3*^KO*OAS1*^KO A549 cells after poly(I:C) (1.0 μg/ml) treatment for 4 h. (**B**) Western blot analysis of RIG-I and MDA5 in single or double gene knockout cells (as indicated on top) treated with IFN-β (20 ng/ml) for 24 h. (**C**) RT-qPCR analysis of *IFNB1* mRNA in WT, *DDX58*^KO and *IFIH1*^KO after SeV infection for 24 h or EMCV infection for 6 h. Data are shown as mean ± SEM of three or four independent experiments. Two-sided Student's *t* test; $^{**}P < 0.01$, $^{***}P < 0.001$; ns, not significant. *P* values = 0.007922 (Sev, WT vs *DDX58*^KO), 0.182148 (Sev, WT vs *IFIH1*^KO), 0.853967 (EMCV, WT vs *DDX58*^KO), 0.000002 (EMCV, WT vs *IFIH1*^KO). (**D**) RT-qPCR analysis of *IFNB1* mRNA in WT and indicated gene knockout cells after poly(I:C) treatment (0.3 μg/ml) for 4 h. Fold change of *IFNB1* mRNA compared to mock-treated WT cells is shown. Data are shown as mean ± SEM of four independent experiments. Two-sided Student's *t* test; $^{***}P < 0.001$. *P* values = 0.000007 (WT vs *DDX58*^KO), <0.000001 (WT vs *SKIV2L*^KO), <0.000001 (*SKIV2L*^KO vs *SKIV2L*^KO*DDX58*^KO). (**E**, **F**) *DDX58*^KO cells were treated with HMW poly(I:C) or IFN-β. Expression of ISG and IFNB1 were analyzed using RT-qPCR. Data are shown as mean ± SEM of three independent experiments. Source data are available online for this figure.

 © The Author(s)

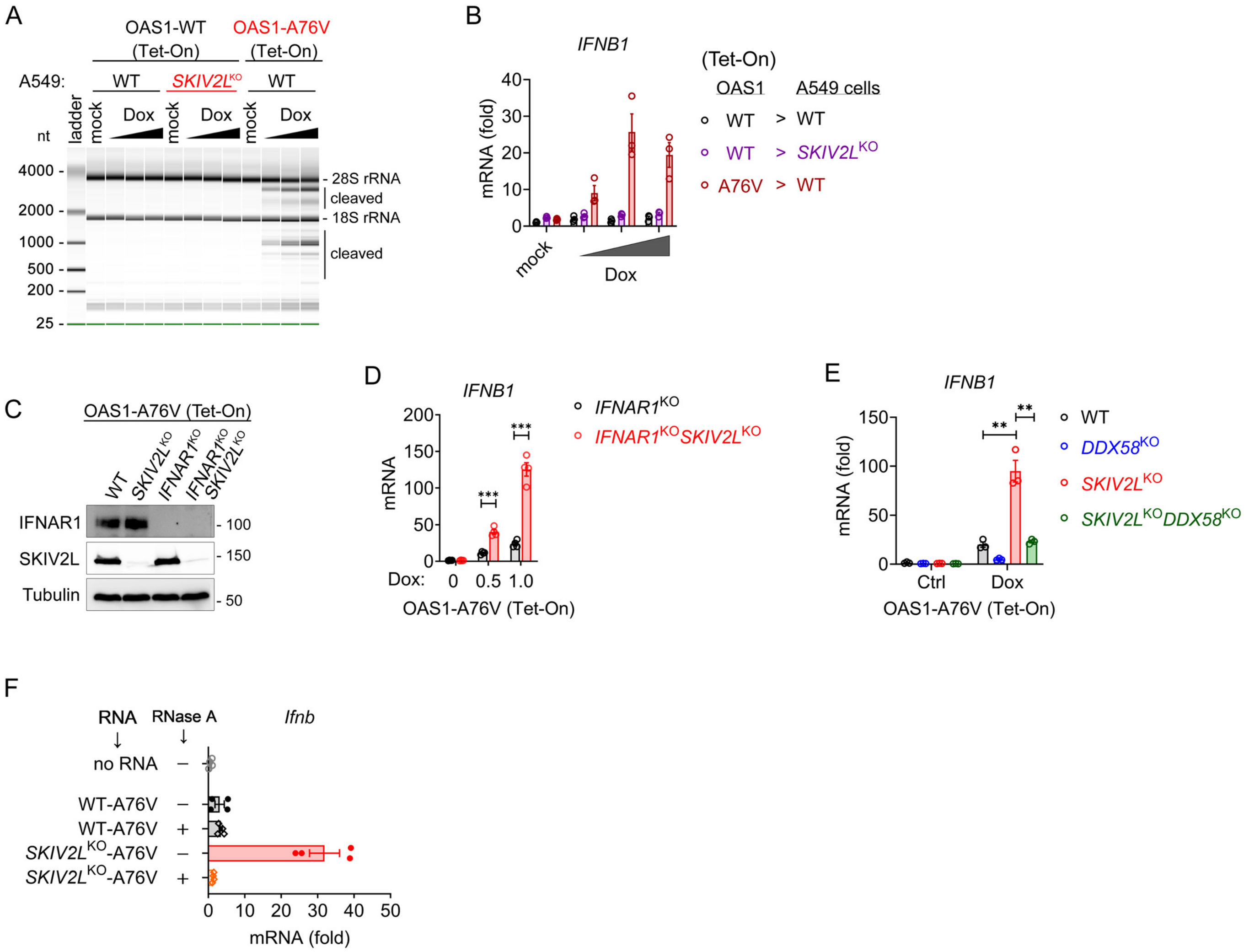

Figure EV3. SKIV2L restricts OAS1-mediated autoinflammation.

(**A**) rRNA cleavage analysis of total RNA in WT or *SKIV2L*^{KO} cells after induction of WT or A76V mutant OAS1 with doxycycline (1.0 µg/ml) for 24 h. Data are representative of at least three independent experiments. (**B**) RT-qPCR analysis of *IFNB1* mRNA in WT or *SKIV2L*^{KO} cells after induction of WT or A76V mutant OAS1 with doxycycline (1.0 µg/ml) for 24 h. Data are shown as mean ± SEM of three independent experiments. (**C**) Western blot analysis of IFNAR1 and SKIV2L proteins in WT and indicated knockout cells stably expressing inducible A76V mutant OAS1. (**D**) RT-qPCR analysis of *IFNB1* mRNA in in WT and indicated knockout cells after induction of A76V mutant OAS1 with doxycycline (0.5, 1.0 µg/ml) for 24 h. Data are shown as mean ± SEM of three independent experiments. Two-sided Student's *t* test; ***$P < 0.001$. *P* values = 0.000086 (Dox 0.5), 0.000036 (Dox 1.0). (**E**) RT-qPCR analysis of *IFNB1* mRNA in WT, *SKIV2L*^{KO}, *DDX58*^{KO}, *SKIV2L*^{KO} *DDX58*^{KO} cells after induction of OAS1 A76V mutant with doxycycline (1.0 µg/ml) for 24 h. Data are mean ± SEM of three independent experiments. Two-sided Student's *t* test; **$P < 0.01$. *P* values = 0.002764 (WT vs *SKIV2L*^{KO}), 0.002985 (*DDX58*^{KO} vs *SKIV2L*^{KO} *DDX58*^{KO}). (**F**) MEFs were transfected with RNA (1.0 µg/ml) isolated from WT and *SKIV2L*^{KO} A549 cells after induction of OAS1 A76V mutant (dox 1.0 µg/ml for 24 h) with or without RNase A treatment. Expression of *Ifnb* was measured by RT-qPCR. Data are mean ± SEM of four independent experiments. Source data are available online for this figure.

© The Author(s)
